# Whole-exome sequencing identifies common and rare variant metabolic QTLs in a Middle Eastern population

**DOI:** 10.1038/s41467-017-01972-9

**Published:** 2018-01-23

**Authors:** Noha A. Yousri, Khalid A. Fakhro, Amal Robay, Juan L. Rodriguez-Flores, Robert P. Mohney, Hassina Zeriri, Tala Odeh, Sara Abdul Kader, Eman K. Aldous, Gaurav Thareja, Manish Kumar, Alya Al-Shakaki, Omar M. Chidiac, Yasmin A. Mohamoud, Jason G. Mezey, Joel A. Malek, Ronald G. Crystal, Karsten Suhre

**Affiliations:** 1 Genetic Medicine, Weill Cornell Medicine-Qatar, PO Box 24144, Doha, Qatar; 2 Computer and Systems Engineering, Alexandria University, Alexandria, Egypt; 3 Sidra Medical Research Center, Department of Human Genetics, PO Box 26999, Doha, Qatar; 4 Genetic Medicine, Weill Cornell Medicine, New York, NY 10065 USA; 5 Metabolon Inc, Durham, NC 27560 USA; 6 Physiology and Biophysics, Weill Cornell Medicine-Qatar, PO Box 24144, Doha, Qatar; 7 Genomics Core, Weill Cornell Medicine-Qatar, PO Box 24144, Doha, Qatar

## Abstract

Metabolomics-genome-wide association studies (mGWAS) have uncovered many metabolic quantitative trait loci (mQTLs) influencing human metabolic individuality, though predominantly in European cohorts. By combining whole-exome sequencing with a high-resolution metabolomics profiling for a highly consanguineous Middle Eastern population, we discover 21 common variant and 12 functional rare variant mQTLs, of which 45% are novel altogether. We fine-map 10 common variant mQTLs to new metabolite ratio associations, and 11 common variant mQTLs to putative protein-altering variants. This is the first work to report common and rare variant mQTLs linked to diseases and/or pharmacological targets in a consanguineous Arab cohort, with wide implications for precision medicine in the Middle East.

## Abstract (web summary)

Blood metabolites are influenced by a combination of genetic and environmental factors. Here, Yousri and colleagues perform a whole-exome sequencing study in combination with a metabolomics analysis to identify metabolic quantitative trait loci in a Middle Eastern population.

## Introduction

Metabolites represent functional intermediates to the end phenotype, can be conserved over several years’ time frame, and can uniquely identify individuals^1^. Several studies have shown that they are influenced by a combination of genetics and environment, the latter comprising both life style exposures and microbial interactions^2^. Recent technological improvements have enabled the accurate detection of thousands of metabolites (collectively, the metabolome), adding highly informative downstream read-outs supporting genetic and transcriptomic signatures in the study of personalized medicine^2^. With the abundance of such omics data, it will become possible to infer causal relationship between constitutive genetic variants and metabolite levels to accurately predict the likelihood of developing pathophysiologic signatures, as a normal individual progresses into a disease state.

To date, there have been several large-scale genome-wide association studies for metabolic traits (mGWAS)^3–10^ the largest (with broad non-targeted metabolomics) of which interrogated 7000 individuals of European ancestry and discovered 145 significant metabolomics quantitative trait loci (mQTLs)^11^. While studies to date have uncovered hundreds of mQTLs, they have also faced certain limitations. First, they relied on imputed genotyping array data for the discovery of common variant mQTLs, where high-quality SNPs are in non-coding regions. More recently, next-generation sequencing (NGS) technologies have become more affordable and begun to identify protein-coding variants largely affecting metabolite levels, yet on a small scale of individuals or metabolites^12–14^ and very recently on a larger scale^15^ (published while finalizing this manuscript). Second, metabolite detection platforms continue to rapidly improve, and deeper resolution can be gained today than previously possible. Third, as with lack of diversity in most GWAS studies^16^, most mGWAS to date have focused predominantly on populations of European descent^7,8,11,17^, and recently, Asian and African descent^18^, yet little or no efforts have been described in Middle Eastern populations.

We present the first large-scale metabolomics exome-wide association study of a highly consanguineous Middle Eastern population, by combining 1,303 metabolites (from the most recent Metabolon DiscoveryHD4 platform) with deep whole-exome sequencing data of 614 Qataris for discovery, and imputed array data of another 382 Qataris for replication analysis. We integrate this data to discover loci affecting metabolites and metabolite ratios in this population, while fine-mapping loci to putative functional variants at or near sentinel SNPs (a sentinel SNP or sentinel metabolite refers to a lead SNP or a lead metabolite). Moreover, by leveraging elevated consanguinity in this population, we also discover rare variant loci associated with metabolite levels, underscoring the metabolic individuality of subjects from this ethnic population.

## Results

### Subject selection and genotyping

**Fig. 1:**
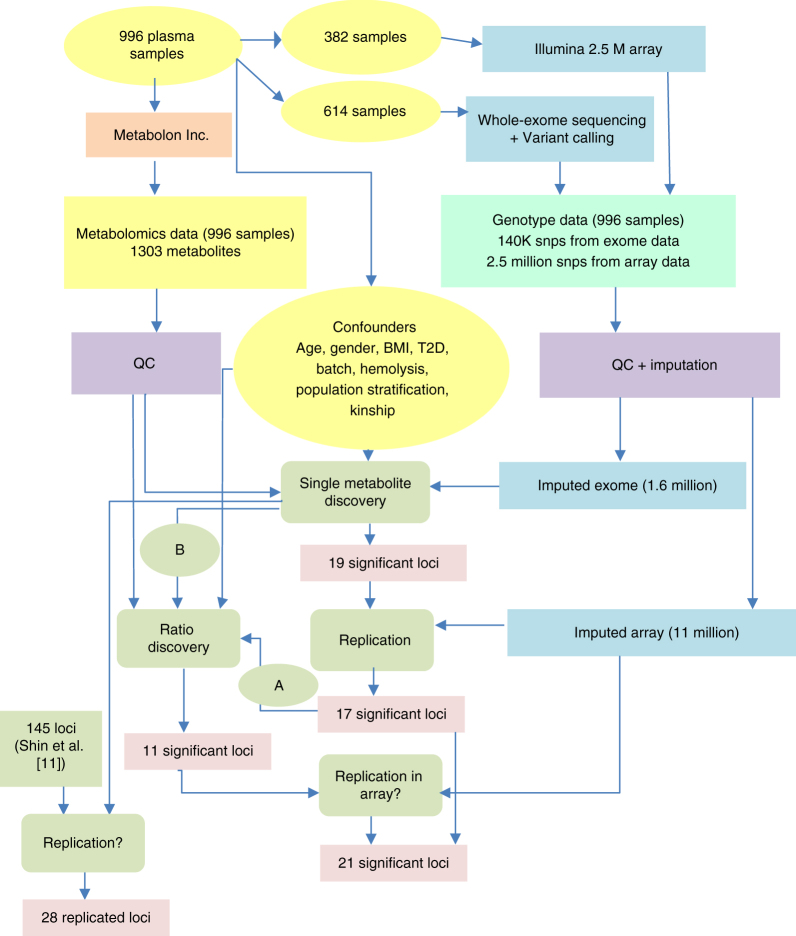
Schematic view of the study design for the common variant analysis. 'A' indicates the first method for ratio computation, where we  computed the associations between SNPs (within 100 Kb of the sentinel SNP from single-metabolite association analysis) and the ratio of the sentinel metabolite to all remaining 825 metabolites. 'B' indicates the second method for ratio computation, where for all SNPs for which two metabolites had been nominally associated in the discovery phase (*p* ≤ 10^−4^), but in opposite directions (opposite beta signs), we computed the association of the given SNP to the ratio of that pair of metabolites

A total of 996 Qatari subjects were selected for this study (Table 1), of whom 614 were whole-exome sequenced (WES) for the discovery step, and 382 were array genotyped for replication. For the purpose of replication, both data sets were imputed using a reference set of 108 deeply covered, phased Qatari genomes. A total of 1,650,892 imputed exome variants were available for the analysis after Quality Control (see Methods). All samples were analyzed on Metabolon’s DiscoveryHD4 platform, where a total of 1303 metabolites were detected (Supplementary Data 1). After applying strict QC, 826 metabolites (including 249 unknowns) remained for the association analysis (Fig. 1 gives a schematic representation of the study design).

**Table 1:** Demographics (sample characteristics)

Demographic category	Attribute
Gender (% females)	45%
Age (mean ± s.d.)	50.1 ± 12.6
T2D (% with diabetes)	56%
BMI (mean ± s.d.)	32 ± 6.6
Population information: #subjects	Q1:442 Q2:339 Q3:70, admixed: 54, not assigned: 91
Genotyping source: #subjects	WES: 614, array genotyped: 382

*WES* whole-exome sequencing

Q1, Q2, and Q3 refer to Bedouin, Persian, and African ancestries, respectively, that present subpopulations of the people of Qatar^36^

### Pre-discovery exploration of replication in Caucasians

As an investigation step prior to discovery, we attempted to replicate 145 previously known loci^11^ in the discovery cohort. We found exact or proxy SNPs (SNPs in LD) for 101 of 145 loci in our dataset, of which sentinel metabolites in 80 loci were matched to metabolites in our cohort. For 13 loci, we replicated associations between the reported SNP and the reported metabolite at a Bonferroni *p*-value (*p* ≤ 0.05/80 = 6.25 × 10^−4^), and for another 15 loci, we replicated the association at the same/proxy SNP but with another metabolite (*p* < 0.05/(101 × 826) = 5.9 × 10^−7^) (Supplementary Data 2). In total, we replicated 28 loci (19.3% of 145 loci, 35% of 80 replicable loci)—including 11 of the 20 most significant loci^11^.

### 21 common variant loci influence metabolites and ratios

To discover all metabolites associated with exome variants in 614 Qataris, 1.6 million imputed exome variants (272,061 SNPs after LD pruning) were tested for association with 826 metabolites (see Supplementary Fig. 1 for distribution of kinship-based heritability estimates for each super pathway). This step uncovered 3127 significant associations (Bonferroni *p* ≤ 2.2 × 10^−10^) (Supplementary Data 7), with an average inflation factor of 0.98 (range: 0.83–1.05). Those associations collapsed into 19 independent loci (see Methods), which we attempted to replicate (based on the sentinel SNP and the sentinel metabolite) in the imputed array data set (*n* = 382) and found that 17 of them replicated (*p* ≤ 0.05/19 = 0.0026) and one nominally replicated at *p* ≤ 0.05 (Table 2, Supplementary Datas 3–5).

**Table 2:** 21 Unique locus-metabolite pairs, indicating 7 newly identified and novel loci, 10 loci fine mapped with new metabolite ratio associations, and 11 protein coding variants

**Locus**	**rsID**	**Metabolite/Ratio**	***p*** **-value (pgain** ^**a**^ **)**	**Beta**	**Annotation** ^**b**^	**r2 (%)** ^**c**^	**Ref/NL/NAS** ^**d**^	**Replication** ***p*** **-value** ^e^
*NAT2*	rs4646257	$\frac{\textstyle{{{5 {\hbox {-}} {\mathrm acetylamino} {\hbox {-}} 6 {\hbox {-}} {\mathrm amino} {\hbox {-}} 3 {\hbox {-}} {\mathrm methyluracil}}}}}{{\textstyle{\mathrm{1 {\hbox {-}} methylxanthine}}}}$	2.23 × 10^−58^ (6.83 × 10^46^)	0.970	IG, **NFS:rs1801280, p.Ile114Thr**	31	(^11,15^) NAS	
	rs4921913	5-acetylamino-6-amino-3-methyluracil	2.14 × 10^−12^	0.499		7.8	(^11,15^)	9.6 × 10^−9^
*ACADS*	rs1799958	Ethylmalonate	2.18 × 10^−53^	0.885	**p.Gly209Ser**	28.5	(^11,15^)	2.1 × 10^−21^
*NAT8*	rs13538	$\frac{\textstyle{{\mathrm{2 {\hbox {-}} aminooctanoate}}}}{{\textstyle{\mathrm{X {\hbox {-}} 12511}}}}$	4.4 × 10^−47^ (8.6 × 10^35^)	−0.781	**p.Phe143Ser**	26.6	(^11,15^) NAS	
	rs13538	N-acetylcitrulline	5.5 × 10^−32^	0.780		19.6	(^11,15^)	3.7 × 10^−21^
*TMPRSS11E*	rs34109652	X-11491 (deoxycholic acid glucuronide or isomer)	3.28 × 10^−35^	−0.737	INT	21.4	(^15^)	3.8 × 10^−25^
*SLCO1B1*	rs4149056	glycochenodeoxycholate glucuronide (1)	3.06 × 10^−31^	0.833	**p.Val174Ala**	18.5	(^11,15^) NAS	5.5 × 10^−22^
*PYROXD2*	rs2147896	N-methylpipecolate	9.13 × 10^−26^	−0.663	**p.Met461Thr**	18.3	(^11^) NAS	7.4 × 10^−19^
*UGT3A1*	rs3756669	$\frac{\textstyle{{\mathrm{X {\hbox {-}} 24348}}}}{\textstyle{{\mathrm{pregn}}\,{\mathrm{steroid}}\,{\mathrm{monosulfate}}^ \ast }}$	1.55 × 10^−25^ (4.02 × 10^12^)	−0.915	**p.Cys121Gly**	16.6	(^11^) NAS	
	rs3756669	X-24348	6.25 × 10^−13^	−0.712		8.5	(^11^) NAS	1.01 × 10^−9^
*FADS2*	rs28456	$${\frac{{{\mathrm{1 {\hbox {-}} }}\left( {{\mathrm{1 {\hbox {-}} enyl {\hbox {-}} palmitoyl}}} \right){\mathrm{ {\hbox {-}} 2 {\hbox {-}} arachi {\hbox {-}} donoyl {\hbox {-}} GPC}}\\ \left( {{\mathrm{P {\hbox {-}} 16:0/20:4}}} \right)^ \ast }}{{{\mathrm{X {\hbox {-}} 24438}}\left( {{\mathrm{PC}}\left( {{\mathrm{P {\hbox {-}} 16:0/20:3}}} \right)} \right)}}}$$	9.81 × 10^−25^ (5.38 × 10^16^)	−0.641	INT	17.8	(^11,15^) NAS	
	rs174560	X-24439 (PE(P-16:0/20:3)^*^)	9.09 × 10^−14^	0.457	INT	8.9	(^11,15^)	1.6 × 10^−3^
*AGXT2*	rs37370	3-aminoisobutyrate	8.45 × 10^−21^	0.810	**p.Ser102Asn**	13	(^15^)	4.3 × 10^−10^
*PHYHD1* ^f^	rs181856093	X-22145 (2′-O-methyluridine)	3.29 × 10^−20^	0.531	INT, **NFS: rs2302811, INS: c.3662-4A** **>** **G**	12.7	NL	1.3 × 10^−11^
*THEM4*	rs6690449	$\frac{\textstyle{{\mathrm{X {\hbox {-}} 18921}}}}{\textstyle{{\mathrm{X {\hbox {-}} 23680}}}}$	9.35 × 10^−20^ (2.29 × 10^9^)	−0.547	INT	14.1	(^11,15^) NAS	
	rs2999534	X-23293	1.15 × 10^−11^	0.426	IG	7.2	(^11,15^)	2.05 × 10^−4^
*UGT1A1*	rs78461713	bilirubin (E, E)^*^	5.1 × 10^−17^	0.484	INT	10.6	(^11,15^)	1.4 × 10^−12^
*SULT2A1*	rs62129970	$\frac{\textstyle{{\mathrm{X {\hbox {-}} 11440}}\left( {{\mathrm{tentatively}}\,{\mathrm{steroid}}} \right)}}{\textstyle{{\mathrm{4 {\hbox {-}} androsten {\hbox {-}} 3alpha}},{\mathrm{17alpha {\hbox {-}} diol}}\,{\mathrm{monosulfate}}\left( {\mathrm{2}} \right)}}$ ^62^	7.59 × 10^−17^ (7.33 × 10^10^)	−0.951	IG	11	(^11^)	1.4 × 10^−17^
*SLC22A24* ^f^	rs78176967	$\frac{\textstyle{{\mathrm{X {\hbox {-}} 22379}}\left( {{\mathrm{androsterone}}\,{\mathrm{glucuronide}}} \right)}}{\textstyle{{\mathrm{21 {\hbox {-}} hydroxypregnenolone}}\,{\mathrm{disulfate}}}}$	1.43 × 10^−16^ (6.75 × 10^6^)	0.995	IG	10.8	(^15^), NAS	
	rs61285056	X-22379 (androsterone glucuronide)	9.11 × 10^−11^	0.765	INT	6.9	(^15^)	5.2 × 10^−8^
*SPTLC1P4* ^f^	rs2069258	$\frac{\textstyle{{\mathrm{X {\hbox {-}} 23293}}}}{\textstyle{{\mathrm{cis {\hbox {-}} 4 {\hbox {-}} decenoyl}}\,{\mathrm{carnitine}}}}$	1.71 × 10^−16^ (3.69 × 10^8^)	0.425	IG	10.6	NL	2.4 × 10^−6^
*TTC38* ^f^	rs117135869	$\frac{\textstyle{{\mathrm{X {\hbox {-}} 22162}}}}{\textstyle{{\mathrm{X {\hbox {-}} 24513}}}}$	4.264 × 10^−16^ (2.06 × 10^5^)	0.623	**p.Ala12Val**	10.4	(^15^) NAS	
	rs117135869	X-22162	8.8 × 10^−11^	0.616		6.7	(^15^)	1.7 × 10^−4^
*SLC22A5*	rs274554	Tryptophan betaine	1.05 × 10^−13^	−0.430	INT	8.6	(^11,15^)	3.3 × 10^−10^
*CCBL2* ^f,g^	rs7530513	Imidazole lactate	5.6 × 10^−13^	0.425	INT	8.1	(^15^)	3.2 × 10^−2^
*SLC17A1*	rs1165196	$\frac{\textstyle{{\mathrm{X {\hbox {-}} 12824}}\left( {{\mathrm{hexanoylglutamine}}} \right)}}{\textstyle{{\mathrm{X {\hbox {-}} 16087}}}}$	1.46 × 10^−12^ (1.30 × 10^6^)	−0.543	**p.Thr269Ile**	8.8	(^11,15^) NAS	8.5 × 10^−8^
*CYP3A5*	rs776746	X-12063	1.54 × 10^−12^	−0.620	**SA, c.219-237A** **>** **G**	7.8	(^11,15^)	1.2 × 10^−12^
*SEMA4B* ^f^	c15p90683852	Undecanedioate	1.18 × 10^−10^	0.421	IG	7	NL	9.7 × 10^−4^

Biochemical Name^*^ indicates compounds that have not been officially confirmed based on a standard, but Metabolon is confident in its identity

p.## (missense), bold font indicates functional variant

^a^pgain was introduced in^19^ as “an ad-hoc measure to determine whether a ratio between two metabolite concentrations carries more information than the two corresponding metabolite concentrations alone”, calculated as pgain = min (pval(*m1)*,pval(*m2*))/pval(*m1/m2*), given two metabolites *m1* and *m2*

^b^SNP Annotation and Nearest Functional SNP (NFS), function, mutation, IG refers to intergenic, INT (Intron), INS (Intron near splice), SA (Splice Acceptor)

^c^r2 is percent of variance explained

^d^Reference or Novel Locus (NL) or Novel Association (NAS)

^e^Exact replication SNP is indicated in Supplementary Dataset 4

^f^Newly identified/novel loci

^g^Newly identified nominally replicated

In addition to single-metabolite-variant associations, we examined each locus to identify significantly associated metabolite ratios^19^. To limit multiple testing, two approaches were used to examine associations of metabolic ratios. First, we computed the associations between SNPs (within 100 Kb of the sentinel SNP from single-metabolite association analysis) and the ratio of the sentinel metabolite to all remaining 825 metabolites. Second, for all SNPs where two metabolites had been nominally associated in the discovery phase (*p* ≤ 10^−4^) but in opposite directions (opposite beta signs), we computed the association of the given SNP to the ratio of that pair of metabolites. Both *p*-value and *p*-gain^19^ thresholds were used to find significant ratios. A total of 11 significant SNP to metabolite ratio associations were discovered with *p* ≤ 0.05/(826 × 18 + 826 × 272,061) = 2.2 × 10^−10^ and *p*-gain ≥(1/(2 × 0.05) × (826 × 18) = 1.48 × 10^5^ (see Methods). Seven of these resulted in metabolic fine-mapping of seven of the loci discovered in the single-metabolite phase (i.e. where a ratio replaced the single metabolite as the sentinel association), whereas the remaining four were new associations at loci not associated with single metabolites. Only three of those four replicated in the cohort of 382 individuals (Table 2, and Supplementary Datas 3–5), resulting in a total of 10 SNP to metabolite ratio associations.

**Fig. 2:**
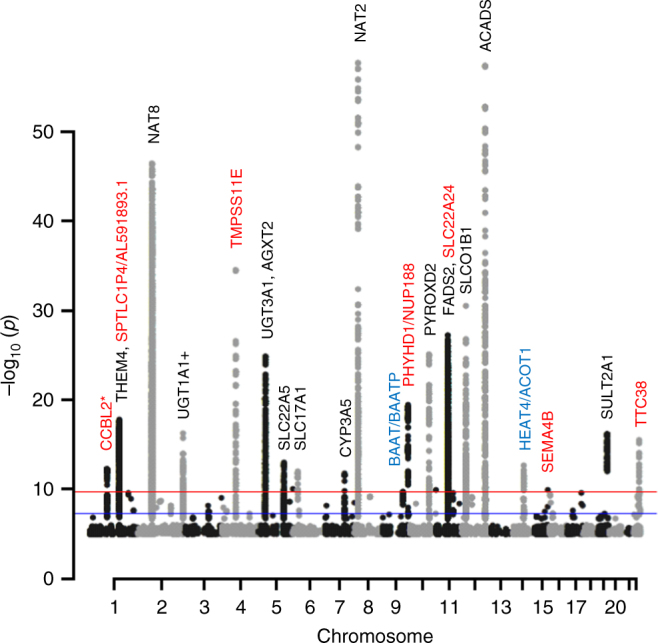
Manhattan plot for the discovered loci. The red line indicates the Bonferroni threshold (2.2 × 10^−10^) and the blue line indicates the genome wide significance threshold (5 × 10^−8^). The newly identified/novel replicated loci are typed in red, and the non-replicated loci are in blue. Unnamed loci at the borderline (2.2 × 10^−10^) are associations with ratios with a *p*-gain below the threshold. *stands for nominally replicated loci

**Fig. 3:**
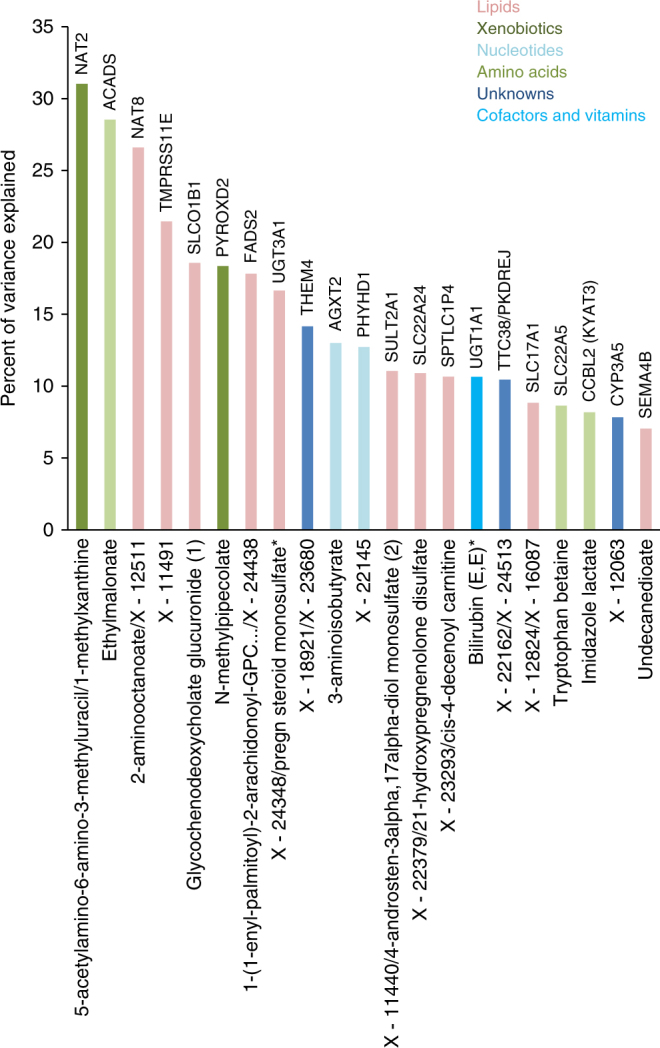
Percent of variance explained in the 21 loci. The height of a column bar indicates the percent of variance explained for each locus, loci genes are indicated above the column bar, and the metabolite/ratio on the *X*-axis. Bars are colored according to Metabolon pathway specified for the metabolites associated with the locus. Biochemical Name* indicates compounds that have not been officially confirmed based on a standard, but Metabolon is confident in its identity

Combined, we discovered 21 unique metabolite and metabolite ratio quantitative trait loci (mQTLs) in Qataris (Fig. 2, and Supplementary Figs. 2 and 3). The variance explained by these genetic variants ranges from the highest value of 31% (5-acetylamino-6-amino-3-methyluracil/1-methylxanthine with rs4646257 in *NAT2* locus) to 7% (association of undecanedioate with c15p90683852 in *SEMA4B* locus) with an average of 14.8% (Table 2, Fig. 3). Of the 21 loci, 7 (31%) were unknown to studies that used older metabolomics platforms (studies prior to 2017) and are defined here as newly identified loci (four loci were concurrently identified in a study published while finalizing this manuscript^15^, and three loci are novel (Table 2, Supplementary Data 3). Five of those seven loci might be new due to the use of the new metabolomics platform, another two (*TMPRSS11E* and *SEMA4B*) were not discovered in ref. ^11^, and other known loci were fine-mapped to new metabolites, and not reported elsewhere (as rs2147896 in *PYROXD2* with *N*-methylpipecolate, and *UGT3A1* with X-24348). We explored the frequencies of the sentinel SNPs in the Genome Aggregation Database (gnomAD)^20^ (Supplementary Data 3), and found that among the seven loci, the sentinel SNP in two novel ones are not reported in the database (*PHYHD1/NUP188, SEMA4B*), and those in another two loci (*SLC22A24, TTC38/PKDREJ*) are rare in other populations.

**Fig. 4:**
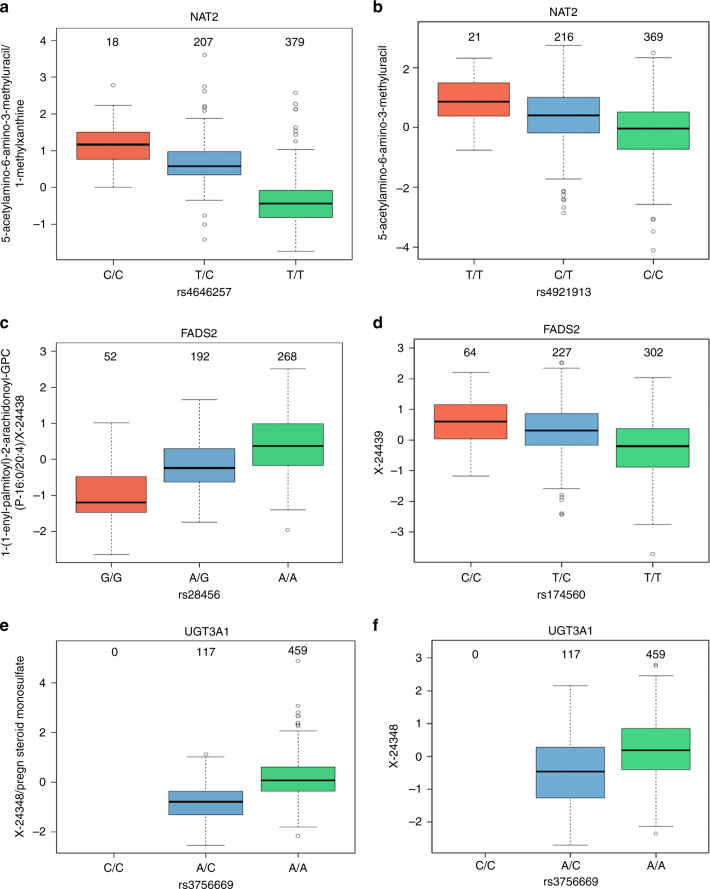
Boxplots for the loci *NAT2*, *FADS2*, and *UGT3A1*. Boxplots showing metabolite/ratio levels and number of samples for each genotype group and comparing ratios to single metabolites for *NAT2* (**a**, **b**), *FADS2* (**c**, **d**), and *UGT3A1* (**e**, **f**) loci, where the percent of variance explained by the ratio is 3.9-, 1.99-, and 1.94-fold greater, respectively, than that explained by the single metabolite

All 10 reported metabolite ratio associations represent metabolic fine-mapping of loci discovered here or in previous studies. According to this cohort, those ratios associate more strongly than the single metabolites previously reported^11,15^. These include the association of rs4646257 in *NAT2* locus with the ratio 5-acetylamino-6-amino-3-methyluracil/1-methylxanthine, rs375811360 in *NAT8* locus with 2-aminooctanoate/X-12511 (X-12511 is possibly 2-acetamidooctanoic acid as identified by Metabolon), rs3756669 in *UGT3A1* locus with X-24348/pregn-steroid-monosulfate, rs28456 in *FADS2* locus with 1-(1-enyl-palmitoyl)-2-arachidonoyl-GPC(P-16:0/20:4)/X-24438 (X-24428 is PC(P-16:0/20:3) as identified by Metabolon), rs6690449 in *THEM4* locus with X-18921/X-23680 and rs1165196 in *SLC17A1* locus with X-12824/X-16087 (X-12824 is hexanoylglutamine as identified by Metabolon) (see expanded loci associations–Supplementary Data 5). Notably, the variance explained by ratios was much higher than that explained by any single metabolite for the same locus (Table 2); for example, for the *NAT2* locus, the variance explained by the ratio of metabolites is 3.9-fold greater than that explained by the single metabolite; similarly, 1.9-fold greater in the *FADS2*, *UGT3A1* and *THEM4* loci, 1.5-fold greater in *SLC22A24* and *TTC38*/*PKDREJ* loci, and 1.3-fold greater in the *NAT8* locus (comparison of ratios to single metabolites—Fig. 4).

We investigated the 21 loci for known eQTLs and disease or pharmacological associations using several databases (biological details are in Supplementary Data 6): GTEx portal (version 2.1, Build #201), OMIM database^21^, Orphanet disease database, CHEMBL targets database, PharmaGKB^22^ and SNIPA (see URLs in Methods). We found that seven sentinel SNPs are in genes encoding enzymes or transporters. Additionally, 16 sentinel SNPs are known eQTLs, including 5 at novel and newly identified loci (*PHYHD1*/*NUP188*, *TTC38*/*PKDREJ*, *TMPRSS11E*, *SPTLC1P4*/*AL591893.1*, *CCBL2*). Moreover, 10 loci contain genes linked to diseases, including 4 of the newly identified loci; namely, *NUP188* has a role in heterotaxy^23^, *SEMA4B* in hypoxia and lung cancer^24^, *CCBL2* in neurodegenerative diseases, and *TMPRSS11E* in squamous cell carcinomas^25^. Finally, genes in 4 loci have been previously studied for effect on drugs.

### Fine-mapping identifies functional variant associations

**Fig. 5:**
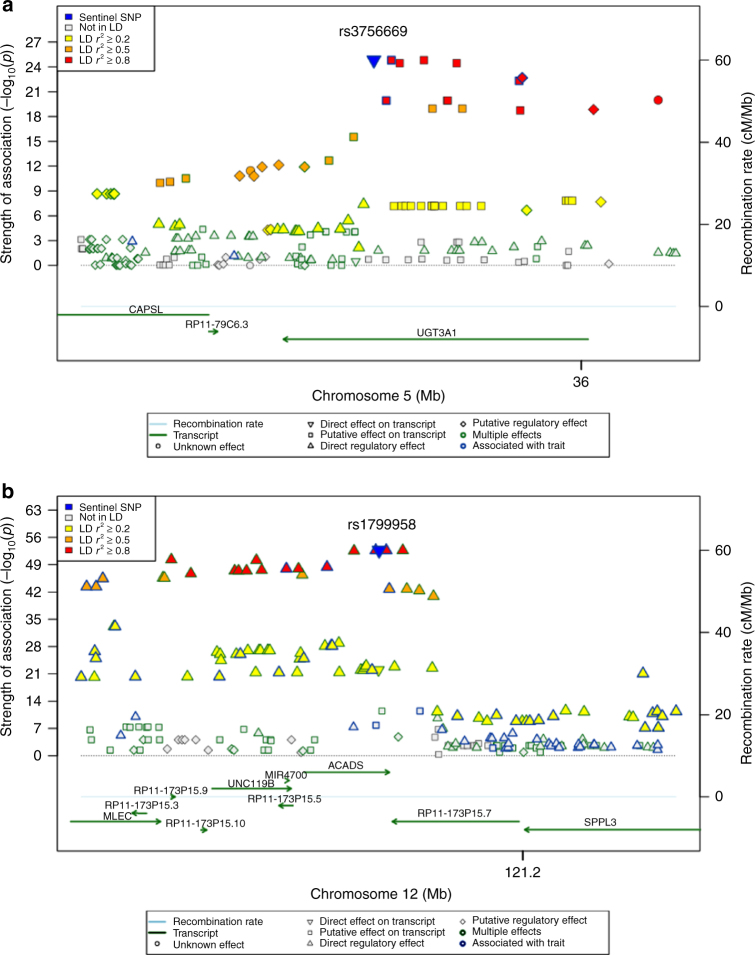
Regional association plots for the loci *UGT3A1* and *ACADS. UGT3A1* (**a**) and *ACADS* (**b**) loci missense SNPs showing the strength of the association (−log10 (*p*-value)) for X–24348/pregn-steroid-monosulfate* and ethylmalonate, respectively, on the *Y*-axis and the genes on the *X*-axis. The colors correspond to different LD thresholds, where LD is computed between the sentinel SNP (lowest *p*-value, colored in blue) and all SNPs. Shapes of markers correspond to their functionality as described in the legend

The availability of WES data in this study population allowed us to investigate if specific loci could be explained by functional (protein-altering) variants. Among the 21 loci, 11 (51%) harbored SNPs that were protein-altering or splice variants (Table 2, Supplementary Data 3). Two of these are among the novel or newly identified loci: the splice variant c.3662-4 A > G (rs2302811) in *NUP188 (PHYHD1/NUP188* locus*)*, association with X-22145 (2′-*O*-methyluridine) and the missense variant p.Ala12Val (rs117135869) in *TTC38* (*TTC38/PKDREJ* locus) association with X-22162/X-24513, and the remaining nine are in previously reported mQTLs (Table 2). More importantly is the missense p.Cys121Gly (rs3756669) in *UGT3A1* (Fig. 5a) which was found to be associated with undetected levels of X-24348 (these appear as missing values in the raw unprocessed metabolite levels) in the subjects which are homozygotes for the mutation, thus a potential loss of function in a pathway possibly related to the metabolite production^26^ (see Discussion). Additionally, the association of the missense variant p.Gly209Ser (rs1799958) in *ACADS* (Fig. 5b) to ethylmalonate levels (concurrently reported^15^) is of clinical implication due to the association of the mutated allele with mild SCAD deficiency (MIM: 606885.0007).

### Rare variants associated with metabolites in Qataris

**Fig. 6:**
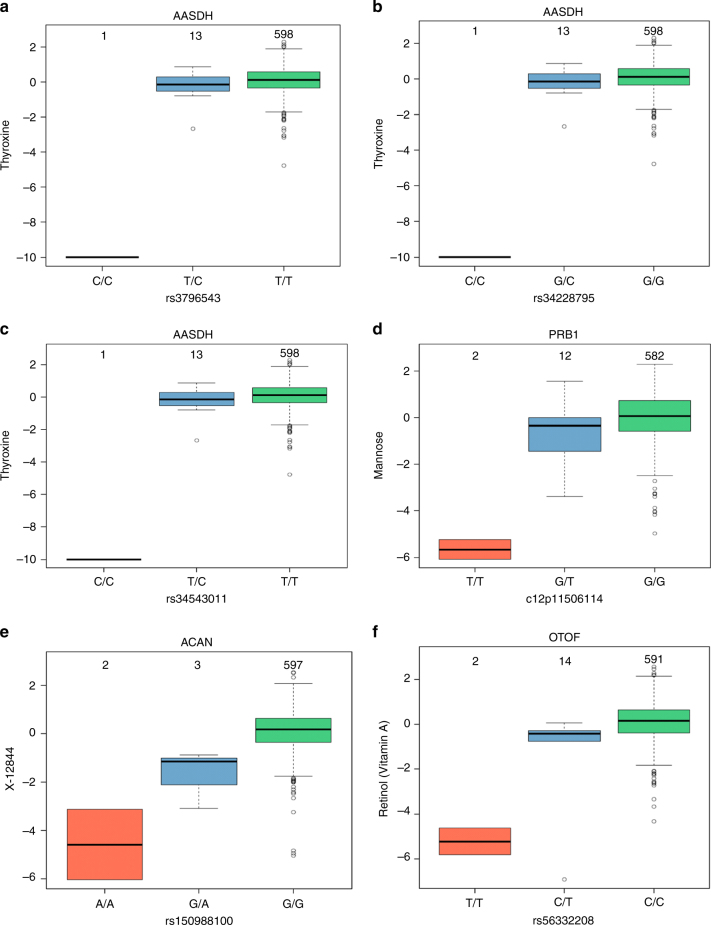
Boxplots for the rare variant loci *AASDH*, *PRB1*, *ACAN*, and *OTOF*, indicating the metabolite level and the number of samples for each genotype group. Boxplots of rare variants associations of *AASDH* with thyroxine (3 SNPs) (**a**–**c**), *PRB1* with mannose (**d**), *ACAN* with X-12844 (**e**), and *OTOF* with retinol (vitamin A) (**f**)

In addition to common-variant mQTL discovery, we hypothesized that with a modest sample size, one may find rare functional variants influencing metabolite levels in homozygous state shared by more than one individual due to high levels of consanguinity. We selected high-quality protein-altering rare variants (MAF < 5%; *n* = 21,933 SNPs, see Methods). First, we performed gene-based burden testing for all genes harboring at least one of these rare variants (*n* = 9823 genes; Bonferroni *p* ≤ 6.16 × 10^−9^); and second, we tested single variant associations for SNPs with at least two homozygotes for the rare variant (*n* = 2660 SNPs in 2119 genes; Bonferroni *p* ≤ 2.27 × 10^−8^). In this analysis, we focused on rare, homozygous variants shared by two or more individuals, whose metabolite values were at either tail of the distribution (highest or lowest). After stringent QC and filtering, we discovered two genes with rare variants influencing metabolite levels through gene-burden analysis. In contrast to burden testing, we identified 10 variants significantly influencing metabolite levels through single-variant testing (Table 3, Fig. 6, Supplementary Fig. 5).

## Discussion

We describe the first large-scale (*n* = 996 individuals) metabolite association study in a Middle Eastern population, combining deep WES data with an updated metabolomics platform. Altogether, we discover and replicate 21 common variant mQTLs. Amongst those, seven are novel or newly identified loci (associations that have been concurrently discovered^15^ and completely new ones, as indicated in Table 2), and 10 represent fine-mapping of previously reported loci to new metabolite ratios that associate more significantly than previously discovered single metabolites. Importantly, by having deep exome data, we fine-map a total of 11 loci to candidate protein-altering variants, with biological implications described below. Further, in the rare variant spectrum, we present 12 novel mQTL associations, all not previously reported.

Being able to replicate 35% of the 80 replicable loci reported in the largest mGWAS study to date^11^ with one tenth the sample size may be attributable to the high levels of consanguinity in our population, which allows for sufficient numbers of alternate allele homozygotes to be present for metabolic associations. Loci replicated among different ethnicities are likely to belong to pathways common to human metabolism (7 out of the 10 most significant loci reported in^11^—*PYROXD2*, *ACADS*, *NAT8*, *FADS2*, *SLCO1B1*, *SULT2A1*, and *UGT1A1*—being among the top associations in our study).

Several of the newly discovered associations may reveal putative biological links between SNPs and metabolites. One such example is the association of rs34109652 in *TMPRSS11E* with X-11491 (tentatively identified by Metabolon as the bile acid deoxycholic acid glucuronide). This particular SNP had been reported as an eQTL for *UGT2B15* (GTEx version 4.1, Build #201), a gene at which CpG methylation was also significantly associated with X-11491 levels^27^, consistent with the gene’s putative function of conjugating bile acids (GenAtlas(http://genatlas.medecine.univ-paris5.fr/fiche.php?n=6643)). Another biological association is that of the missense variant rs117135869 (p.Ala12Val) in *TTC38* (*TTC38*/PKDREJ locus) associating significantly to X-22162, and more significantly, to the ratio of this metabolite to X-24513. *TTC38* (Tetratricopeptide Repeat Domain 38) expression is significantly positively correlated with age^28^, and levels of both metabolites are also significantly associated with age by regression analysis in our cohort (see Methods). Additionally, X-24513 is significantly partially correlated with C-mannosyltryptophan (also known as C-glycosyltryptophan) (see Methods), which was reported to be associated with aging^29^. More interestingly, the fact that this locus encompasses *PKDREJ* (Polycystic Kidney Disease and Receptor for Egg Jelly related protein) gene (regional association plot—Supplementary Fig. 3), a homolog of PKD genes associating with kidney disease^30^, and that X-24513 might be similar in characteristics to C-mannosyltryptophan, reportedly elevated in chronic kidney disease^31^, highlights the importance of investigating the biological implication of this association in relation to both kidney diseases and ageing. Finally, the association of rs7530513 in the aminotransferase *CCBL2* (Cysteine-S-conjugate beta-lyase 2, also known as *KYAT3* (Kynurenine–oxoglutarate transaminase 3) with imidazole lactate is another biologically relevant association; CCBL2 enzyme [EC2.6.1.7] has a transaminase activity towards histidine (Uniprot^32^) and it has been reported that histidine transaminase [EC2.6.1.38], takes L-histidine and 2-oxyglutarate as substrates and produces imidazole pyruvate that is later converted to imidazole lactate^33^. However, we are unable to confirm that the same histidine transaminase is the one involved in both processes.

A main advantage of using WES data in an association analysis is the enhanced resolution at associated loci that could reveal protein-altering variants affecting metabolite levels. We were able to identify such sentinel SNPs for over half of all loci (11 of 21), possibly revealing functional relationships between genes harboring these variants and their associated metabolites. One such example is the fine-mapping of a previously identified signal in the 3′UTR of ACADS to a missense mutation (rs1799958, p.Gly209Ser) in exon 6 (Fig. 5b). This mutation (MIM 606885.0007) has been reported independently as causing mild SCAD deficiency, with the mutant allele predicted to have 85% of wild-type activity^34^. Further, the population-frequency of this variant (MAF ~25% in GnomAD, 37.8% in Qataris) is consistent with it having been the mild functional mutation in previously reported association signals between this locus and levels of both ethylmalonate and butyrylcarnitine, metabolites whose levels are known to be perturbed in SCAD deficiency^35^.

Another important finding is at the *UGT3A1* locus, where, in contrast to previous studies that reported an association of the intergenic SNP rs10491431 with steroid levels, we uncovered an association of the missense mutation rs3756669 (p.Cys121Gly) in *UGT3A1* with the unknown metabolite X-24348 and more strongly with the ratio of X-24348 to pregn-steroid-monosulfate (Figs. 4 and 5). This unknown has shown significant partial correlation to two other metabolites that also significantly correlate with steroids (Supplementary Fig. 4a, see Methods). The missense mutation therefore possibly interferes with native gene function related to the production of this metabolite, since all minor homozygotes for this SNP had missing values of X-24348 (Supplementary Fig. 2) (*p*-value = 5.94 × 10^−12^ for random occurrence of missing values, see Methods), suggesting the amino-acid substitution leads to loss of function of the gene. This hypothesis is supported by previous functional evidence demonstrating that this mutation leads to diminished UGT3A1 glucuronidation activity^26^, especially of bile acids and estradiol. Metabolon has also recently studied this unknown and found that it might be a form of *N*-acetylglucosamine modification of a pregnandiol, yet cannot be confirmed at the time being. Together, the data suggest the unknown metabolite may itself be a conjugated steroid, which requires this conjugation to appear at detectable levels in the blood. Notably, the mutation appears to have population-specific allele distribution, having been observed in up to 40% of Asians, 15% of Europeans, but only 3% of Africans^26^—a distribution which is maintained in our study cohort (overall Qatari MAF 12.8%), being present in Q1 and Q2 Qataris (Bedouin and Persian/South Asian), but not in Q3, who are of African descent^36^.

In addition to identifying functional SNPs, the use of the updated metabolomics platform (DiscoveryHD4) enabled the refinement of previously reported loci to new metabolites or metabolite ratios in the same or in different biological pathway from those originally reported^11^. For example, *NAT2* encodes an arylamine transferase that controls the conversion of paraxanthine to 5-acetylamino-6-amino-3-methyluracil^33^, and its locus has previously been reported to be associated with 1-methylxanthine. In this study, we discovered a stronger association with the ratios 5-acetylamino-6-amino-3-methyluracil/1-methylxanthine and 5-acetylamino-6-amino-3-methyluracil/paraxanthine, which are consistent with the known activity of the NAT2 enzyme in caffeine metabolism (see network of metabolites linked to NAT2, and KEGG pathway in Supplementary Fig. 4b, c). Being the top most significant association in this population in comparison to the top most ones previously reported^11,15^, it might have several biological implications for the studied population. It is thus worthy to note that the two missense SNPs at this locus are identified in OMIM as associated with the rate of acetylation, presenting a possible mechanism for affecting drug metabolism^22^; specifically, slow acetylation was reported for individuals harboring either of the two missense SNPs rs1799930 and rs1801280 (MIM: 612182.0001 and 612182.0002). Additionally, we discovered associations between *SLC17A1* and the ratio hexanoylglutamine/X-16087. Previously, rs1185567 in *SLC17A3* was reported to be associated with steroid levels^11^. However, since the SLC17 family are vesicular glutamate transporters^37^, our association signal, linking the missense variant p.Thr269Ile (rs1165196) to a ratio containing hexanoylglutamine may reflect a direct biological relationship. We also discovered mQTLs associating known loci to metabolites in pathways different from those previously reported. Once such example is the association between the missense variant rs2147896 (p.Met461Thr) in *PYROXD2* with levels of *N*-methylpipecolate. This gene had previously been linked to levels of urinary trimethylamine^18^ and dimethylamine^5^, yet interestingly not found significantly associated with any of the mentioned metabolites in previous large scale studies^11,15^. Finally, our study expands the associations of several loci with newly identified metabolites or ratios as in the *NAT2* locus and *FADS2* locus among others (Supplementary Data 5). One example that supports the functional importance of the discovered loci is the *SLCO1B1* locus. Its sentinel SNP, rs4149056, was previously associated with the ratio isoleucine/X-11529^11^, and other unknowns, and found in our study to be associated with the bile acids glycochenodeoxycholate-glucuronide(1) (which was also recently found to be the retired form of X-11529 by Metabolon), and glycocholenate sulfate. That supports the functionality of SLCO1B1 as a bile acid transporter, suggesting that the mutation p.Val174Ala alters the gene’s native function^38^.

In addition to common mQTLs, we discovered a total of 12 rare mQTLs—10 by single-variant analysis and 2 by gene-burden analysis—with interesting biological implications (Table 3 and Supplementary Fig. 5). First, the *ACAN* gene encodes a major component of the extracellular matrix, lending important biomechanical properties of cartilage, which explains its role in diseases such as osteoarthritis (MIM: 155760). We discovered an association of a missense variant p.Ala766Thr (rs150988100) in *ACAN* with a steroid (X-12844, tentatively identified by Metabolon as glucuronidated steroid); it is well-established that circulating steroid levels are linked to inflammation in joints and diseases such as arthritis^39–42^. Additional investigations would be needed to uncover the relation between the variant and the metabolite since one of the two subjects having this mutation have musculoskeletal problems. Similarly, the association of rs377301648 in *ZNF133* with a 3-methyl-2-oxovalerate, a metabolite in the branched-chain amino-acid pathway might be due to the role of the gene in osteoblast differentiation^43^ and the previously reported involvement of 3-methyl-2-oxovalerate in osteoarthritis^44^. Additionally, association of the c12p11506114 mutation in PRB1 with mannose (Fig. 6) might be due to the involvement of this glycosylated, proline-rich protein in the salivary secretion pathway^45^, (Michael W King, PhD|© 1996–2016 themedicalbiochemistrypage.org)^46^.

**Table 3:** 12 novel functional rare variant mQTLs

Gene-based burden test							
SNP name (c#chr p#position)	rsID	Gene	Metabolite	EA/OA	C-MAF	*p*-value	
c4p57221348	rs3796543	*AASDH*	Thyroxine	C/T			
c4p57248716	rs34228795	*AASDH*	Thyroxine	C/G	0.036	4.1 × 10^−09^	
c4p57250285	rs34543011	*AASDH*	Thyroxine	C/T			
c12p56075599	rs199581976	*METTL7B*	Androsterone sulfate	T/C			
c12p56075915	rs75289684	*METTL7B*	Androsterone sulfate	A/C	0.035	4.78 × 10^−09^	
c12p56077768	rs115687886	*METTL7B*	Androsterone sulfate	T/C			

Single-variant analysis								
SNP name (c#chr p#position)	rsID	Gene	Metabolite	EA/OA	EAF	*N*	Beta (s.e)	*p*-value
c12p11506114*	-	*PRB1*	Mannose	T/G	0.013	576	−1.32(0.192)	5.99 × 10^−12^
c15p89398112	rs150988100	*ACAN*	X-12844 (glucuronidated steroid)	A/G	0.006	581	−2.05(0.323)	1.88 × 10^−10^
c2p26760624	rs56332208	*OTOF*	Retinol (Vitamin A)	T/C	0.0153	586	−1.18(0.19)	4.83 × 10^−10^
c15p89398112	rs150988100	*ACAN*	X-09789	A/G	0.0059	592	−2.25(0.373)	1.56 × 10^−09^
c18p48256030	-	*MAPK4*	X-21365 (*N*-trimethyl 5-aminovalerate)	C/G	0.0161	589	−1.27(0.22)	6.84 × 10^−09^
c20p18295959	rs377301648	*ZNF133*	3-methyl-2-oxovalerate	C/T	0.0160	592	−1.15(0.2)	9.36 × 10^−09^
c11p18158958	rs61733595	*MRGPRX3*	Tryptophan	T/C	0.0084	592	−1.43(0.25)	1.01 × 10^−08^
c12p55688833	rs372117452	*OR6C6*	Androsterone sulfate	G/A	0.012	590	−1.37(0.24)	1.09 × 10^−08^
c12p56086993	rs144983062	*ITGA7*	Androsterone sulfate	C/T	0.023	590	−1.02(0.18)	1.25 × 10^−08^
c18p48256030	-	*MAPK4*	Tryptophan	G/C	0.016	592	−1.11(0.19)	1.32 × 10^−08^

*EA* effective allele, *OA* observed allele, *EAF* effective allele frequency, *CMAF* cumulative minor allele frequency

All SNPs have a call rate of 100% except for the SNP marked (*), which has a call rate of 97%

Another interesting rare variant association is that between rs56332208 in *OTOF* with retinol (vitamin A). This might be a disease-relevant link because retinoic acid mediates the regeneration of specialized mechano-sensory hair cells in the inner ear that capture auditory and balance sensory inputs, and which die after acoustic trauma, ototoxic drugs or aging diseases, leading to progressive hearing loss^47^. Previous studies have also described mutations in OTOF causing recessive neurosensory non-syndromic deafness in patient cohorts from many different ethnicities via the gene’s role in exocytosis of inner and outer hair cells^48–52^. Thus, our study may provide a mechanistic link between this gene and hair cell development via modulation of retinol levels. Finally, burden-testing revealed mQTLs in *AASDH* and *METTL7B* associated with decreased levels of thyroxine and androsterone sulfate respectively. The *AASDH* gene plays an active role in the pipecolate pathway in which thyroxine is a by-product^53^. Thus, these mutations may impact protein function leading to significant reduction of thyroid hormone and metabolism. Regarding the *METTL7B* mQTL, one of the three mutations in this gene (rs115687886) is a nonsense mutation, suggesting the other two are also loss-of-function mutations as they influence the metabolite in the same direction. Increases in *METTL7B* expression had been previously observed in patients with acute respiratory distress syndrome, involving tissue injury and inflammation^54^, whereas adrenal androgens as androsterone sulfate have been observed to decrease in stress and inflammation^39–42^. The fact that one of the two subjects with the rs199581976 mutation had recurrent chest infections and pneumonia and the other subject had bronchial asthma, recurrent pneumonia, and right lung lower lobe collapse, supports the functionality of the gene, and thus provides another possible link between a rare variant and disease via metabolite-level modulation.

To summarize, our study revealed 21 mQTLs in Qataris, among which 7 are unknown to studies that used older metabolomics platforms, 10 are metabolically fine-mapped with new metabolite ratios, and 11 in which the sentinel SNP was at or in complete LD with protein-altering variants. We also discovered 12 novel functional rare variant mQTLs that are likely specific to this ethnic population. We believe that it is important to replicate rare variant associations, yet the low frequency of these variants and absence of similar cohorts makes it challenging. This study demonstrates the efficiency of using WES for mQTL discovery, which could be more convenient compared to the more expensive WGS, while providing deep coverage of protein coding variants, for fine-mapping of common mQTLs and being suitable for rare variant mQTL discovery. The use of WES is strongly supported by the ability to replicate a fair fraction of the mQTLs in Caucasians (19% of all loci and 35% of replicable loci). Finally, this study is the largest and the first of its kind in a Middle Eastern population, where  we show that studies in consanguineous populations offer a large insight with modest sample sizes and have the potential to reveal loci linked to disease and pharmacological/drug targets important to precision medicine in this region of the world.

## Methods

### Study cohort

Human subjects were recruited and written informed consent was obtained at Hamad Medical Corporation (HMC) and HMC Primary Health Care Centers in Doha, Qatar and approved by the Institutional Review Boards of Hamad Medical Corporation and Weill Cornell Medicine in Qatar. Briefly, a total of 614 subjects were recruited for exome sequencing, and another 382 subjects for array genotyping. Subjects were included on the basis of being 3-generation Qataris (four grandparents born in Qatar) and being healthy or diabetics, above the age of 30 years. Sample demographic characteristics are given in Table 1.

### Exome sequencing

DNA of 614 subjects was extracted from blood using the QIAamp DNA Blood Maxi Kit (Qiagen Sciences Inc, Germantown, MD) and subjected to exome sequencing on the Illumina HiSeq 2000 platform using standard methods. Each subject was sequenced to a minimum mean depth of 70X. Genotypes were generated using the GATK Best Practices workflow^55^ (Whole exome data for a set of recently received 64 samples were merged with those of the 550 samples after calling variants using GATK on each and finding that the majority of SNPs detected in both sets overlap. We limited the analysis to variants in both sets by setting a genotype call rate of ≥98% for common variant analysis and 90% for rare variant analysis). Detailed preparation methods and genotypes are available in^56^. The DNA of 382 subjects was extracted and subjected to array genotyping using Illumina Omni 2.5M array kits.

### Imputation and filtering

Exome and array data were imputed after being filtered (MAF ≥ 0.05, *p*_HWE_ > 10^−6^, genotype call rate ≥98%) based on phased 108 Qatari whole genomes as a reference panel, using shapeit^57^ and Impute2 software packages^58,59^. Throughout all the manuscript and supplementary data sets/information, two unique notations of “c”#“p”# and #:# for indicating a chromosome, position pair (for example c1p100 and 1:100) are used to distinguish original exome SNPs and imputed exome SNPs, respectively. A total of 22 million SNPs were imputed into 614 Qatari Exomes. For common-variant association analysis, 1,650,892 SNPs were used, after removing SNPs with imputation quality *R*^2^ < 0.5, MAF < 0.05, genotype call rate <98%, and pHWE <10^−6^. For rare variant analysis, the methods are described separately below. To define a Bonferroni threshold for finding significant associations, we used the pruned set of SNPs at LD 0.8 (using plink command indep-pairwise 50 5 0.8) that resulted in a total of 272,061 SNPs. Replication was performed in array-genotyped data from a separate set of 382 samples, where imputation using the same 108 Qatari genomes produced a total of 11 million high-quality SNPs.

### Metabolomics data

Serum samples were prepared for metabolomics analysis as follows: 200 μl of serum were aliquoted, barcoded and transported on dry ice to Metabolon Inc. for analysis. Some samples were found hemolyzed, and the degree of hemolysis was recorded for each sample (according to a hemolysis chart as given by Metabolon Inc.), for correcting for its effect on metabolomics measurements. The Metabolon DiscoveryHD4 platform was used (details are given in Supplementary Note 1). This platform utilized a Waters ACQUITY ultra-performance liquid chromatography (UPLC) and a Thermo Scientific Q-Exactive high resolution/accurate mass spectrometer interfaced with a heated electrospray ionization (HESI-II) source and Orbitrap mass analyzer operated at 35,000 mass resolution. A total of 1303 metabolites were measured on that platform. Outlier metabolite measurements (3 standard deviations larger than the mean) were replaced by a missing value to avoid biasing the results. Metabolites with more than 20% missing values were removed from the data. A total of 826 metabolites (including 249 unknown metabolites) survived as high quality and observed in >80% of all individuals. Metabolite measurements were log-scaled and *z*-score normalized. Since samples were collected over different periods of time, and there was no adopted fasting criterion, we addressed that issue by investigating whether there are batch effects in the PCA of metabolic data, and did not find any grouping for any of the 614 exome samples or the 382 array samples. Several unknown metabolites were investigated by the aid of Metabolon, and which revealed their identities (see below).

### Identification of unknown metabolites by Metabolon

Identification of tentative structural features for unknown biochemicals incorporates a detailed analysis of mass spec data, i.e., gathering information such as the accurate monoisotopic mass, the elution time and fragmentation pattern of the primary ion, and correlation to other molecules. The accurate monoisotopic mass is used to identify a likely structural formula for the unknown biochemical, which is then used to search against chemical structure databases (e.g., ChemSpider, SciFinder). When a candidate structure fits the accurate monoisotopic mass and fragmentation data, an authentic standard is commercially purchased or synthesized (when possible). Conformation of a proposed structure is based on a match to three primary criteria, including co-elution with the unknown molecule of interest, and a high degree match to both the accurate monoisotopic mass and fragmentation pattern. When a standard is not available to confirm the identity of the unknown biochemical but sufficient data exist to support a high degree of confidence in its identity, the unknown biochemical may be retired for a named structure that is differentiated from other named metabolites confirmed with authentic standards by the addition of an asterisk after the biochemical name. When a high degree of confidence in the identity of the unknown molecule is not obtained, the molecule retains its unnamed status as designated by an 'X-' in the molecule name. Refer to Supplementary Note 2 for the particular identification information on each of the identified unknown metabolites, and Supplementary Fig. 6 for the comparison of MS^2^ fragmentation spectrum and Extracted Ion Chromatogram of the candidate and unknown metabolite that were structurally confirmed by Metabolon.

### mGWAS analysis

All associations were performed on imputed exome or imputed array genotype data. *P*-values and effect sizes were calculated using functions from both GenABEL and ProbABEL packages in R (version 3.1.2) that were used for computing associations between SNPs and metabolite/ratio levels (after being pre-processed as described in the previous sections) while correcting for age, gender, BMI, T2D, hemolysis, population stratification (using two PCA components) and kinship (for family relatedness). The “polygenic” function in GenABEL was used for correcting metabolite levels for covariates and kinship matrix, and regression analysis using “mmscore” function was used to find association between the residuals obtained from this function and the SNPs using an additive inheritance model. Heritability estimates are based on “*esth2*” values resulting from this function. Kinship matrix was computed based on the genotype data using the “ibd” function in GenABEL. The mean inflation factor for all metabolites was 0.98. Percent of variance is calculated as follows: *r*^2^ = *χ*^2^*/(N* − 2 + *χ*^2^*)*, where *χ*^2^ = (*β*/s.e.)^2^, *β* and s.e. are the *β* and the standard error of the *β*, as obtained from the regression results, and *N* is the number of samples involved in this association. Regional association plots were produced using an in-house tool similar to that of (http://snipa.helmholtz-muenchen.de/snipa/), yet based on LD of subjects in the exome data used here.

### Loci and sentinel SNPs

Association results were divided into 500 Kbp blocks, and in each of these the sentinel SNP and sentinel metabolite are defined according to the SNP-metabolite association with the highest significance. Those define the mQTLs. In the case where a locus had two significant SNPs not in LD (i.e., LD < 0.5) and lying in two different genes, it was broken down into two loci (as in the case of THEM4 gene and SPTLC1P4/AL591893.1, where those two loci are at LD = 0.3). However, where there are two SNPs not in LD (LD < 0.5) but are in the same gene, we did not split the locus and we report LD values for those SNPs (in NAT2 and FADS2 genes) in Supplementary Data 5. All functional variants—missense and splice variants reported are in LD with the sentinel SNP of the locus except for the NAT2 locus where the two missense SNPs are not in LD with the sentinel SNP.

### Ratios analysis

*p*-gain was introduced in ref. ^19^ as an “ad-hoc measure to determine whether a ratio between two metabolite concentrations carries more information than the two corresponding metabolite concentrations alone”. It is calculated as: *p*-gain = min(*p*-val(*m*1), *p*-val(*m*2))/*p*-val(*m*1/*m*2), given two metabolites *m*1 and *m*2, and the *p*-values (*p*-val) of the association of the metabolite/ratio to the same SNP. The *p*-gain threshold according to^19^ assumes a level of significance of *α*/*B*, where a type I error rate of *α* = 0.05 is used, and the critical value for the *p*-gain is *B*/(2⋅*α*), i.e. 10⋅*B*. Thus for Bonferroni correction of *B* tests, the uncorrected critical value of 10 can be multiplied by the number of tests *B*. Accordingly, we set the value of 10 × 826 × 18 (10×number of metabolites×number of loci obtained from single metabolites) as an approximate threshold (we took the maximum of the number of tests of the first  and the second methods where the first method (as mentioned earlier) considers the ratio of the sentinel metabolite against all the rest of 825 metabolites, tested with SNPs in 100 KB of the sentinel SNP of this locus in each of the loci discovered by single-metabolite analysis, and the second method considers the ratio of two metabolites associated with the same SNP but with opposite beta signs, in this case 13,281 associations were tested).

### Fisher exact test for randomness of missing values

This was used to detect whether the missing metabolite values from certain genotype groups (AA, AB, and BB) were missing by chance, when studying the effect of a missense on the metabolite level (after making sure the missingness was not due to removing outliers in the pre-processing of metabolite levels). The two inputs for the Fisher’s exact test are: (a) the number of missing values from each genotype group, and (b) the total number of samples in each genotype group. fisher.test method in R was used for this calculation. The low *p*-value indicates a low probability having these missing values by chance.

### Metabolite regression analysis and partial correlations

To find associations between selected metabolites and other phenotypes as age (as in the case of X-22162 and X-24513), the metabolite level is regressed against age, gender, BMI, hemolysis, T2D, and population stratification (for inclusion of ethnicity) for all 996 individuals using lm function in R. Bonferroni *p*-value is used to report a significant association with a phenotype (*p* ≤ 0.05/826). Partial correlations between two metabolites are calculated using the GeneNet package in R, and significant partial correlations are those which pass a Bonferroni *p*-value of (*p* ≤ 0.05/(826 × 825/2)).

### Rare variants analysis

High-quality rare variants were selected from the original non-imputed exome data, with MAF ≤ 5%, *p*_HWE_ > 10^−6^, and genotype call rate ≥90%. Single-variant analysis was done using GenABEL package in R (as mentioned above for common variants), and burden test was done using seqMeta package in R (as in refs. ^60,61^). For burden tests, we accounted for the number of genes (*n* = 9823 genes; Bonferroni p ≤ 6.16 × 10^−9^), and for single-variant analysis, we accounted for the number of SNPs where we tested single-variant associations for SNPs with at least two homozygotes for the rare variant (*n* = 2660 SNPs in 2119 genes; Bonferroni *p* ≤ 2.27 × 10^−8^). All SNPs in the reported 12 rare variant mQTLs have a call rate of 100% except for c12p11506114 with a call rate of 97%. A filtering step was done by visual inspection of genotype-metabolite boxplots of significant associations to consider only associations where metabolite values of the minor homozygotes are at the extreme tail of the metabolite distribution (i.e., they should be the lowest or highest metabolite values for that metabolite).

### URLs of databases used for annotation

GTEx portal (version 2.1, Build #201)[www.gtexportal.org], OMIM diseases database [www.omim.org], Orphanet disease database [http://www.orpha.net] CHEMBL targets database [www.ebi.ac.uk/chembl], PharmaGKB [www.pharmgkb.org], SNIPA [http://snipa.helmholtz-muenchen.de/snipa/], GenAtlas (http://genatlas.medecine.univ-paris5.fr/fiche.php?n=6643), and GnomAD: http://gnomad.broadinstitute.org/.

### Data availability

All information on metabolites are in Supplementary Data 1, expanded association results for 21 common variant mQTLs are in Supplementary Data 5, and the detailed biological annotations/interpretations of associations are in Supplementary Data 6, list of all associations (*p* < = 1.4 × 10^−7^) are in Supplementary Data 7. All plots are also available in Supplementary Information and detailed information on Metabolon’s method for identification of unknowns is available in the Supplementary Note. Exome data used in this project were selected from a pool of samples of which more than 1000 samples are deposited in SRA accessions SRP060765, SRP061943, and SRP061463, accessible online at http://www.ncbi.nlm.nih.gov/Traces/study/?acc=SRP060765%2CSRP061943%2CSRP061463&go=go). (SRA accession SRP061943).

## Electronic supplementary material

Supplementary Information

Description of Additional Supplementary Files

Supplementary Data 1

Supplementary Data 2

Supplementary Data 3

Supplementary Data 4

Supplementary Data 5

Supplementary Data 6

Supplementary Data 7
