# Supplementary material for: Whole-exome sequencing identifies common and rare variant metabolic QTLs in a Middle Eastern population
Source: Nat Commun. 2018 Jan 23;9:333. doi: 10.1038/s41467-017-01972-9 (PMC5780481; doi:10.1038/s41467-017-01972-9)
Supplement: Supplementary file 1 — Supplementary Information [file 41467_2017_1972_MOESM1_ESM.pdf]

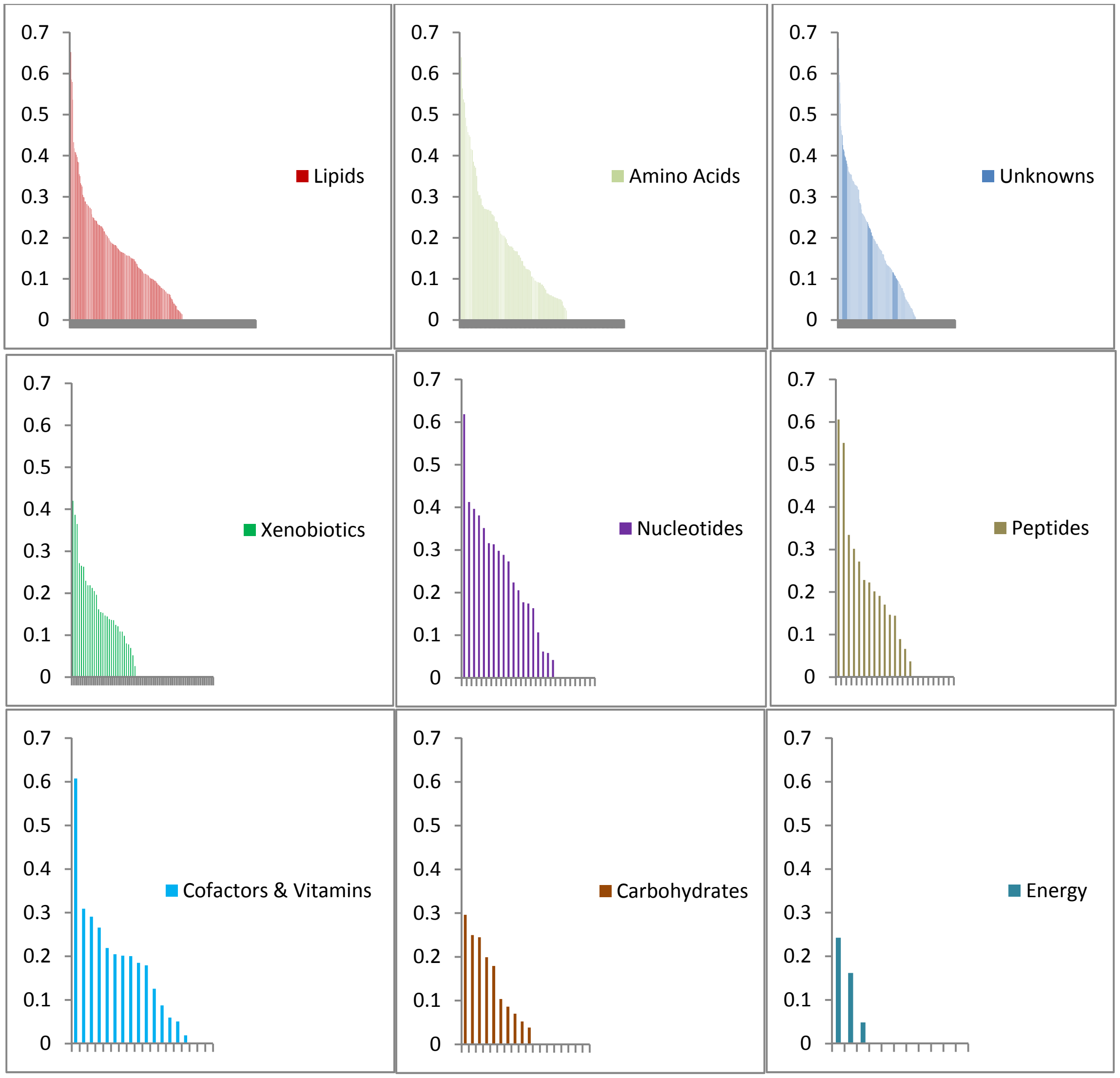

Supplementary Figure 1: Distributions of kinship-based heritability estimates of metabolites for each super-pathway. The y-axis indicates the heritability estimate and the x-axis indicates the metabolites ordered in a descending order of their heritability estimates.

Supplementary Figure 2: Boxplots of 21 common variant loci showing metabolite/ratio levels against the genotype groups.

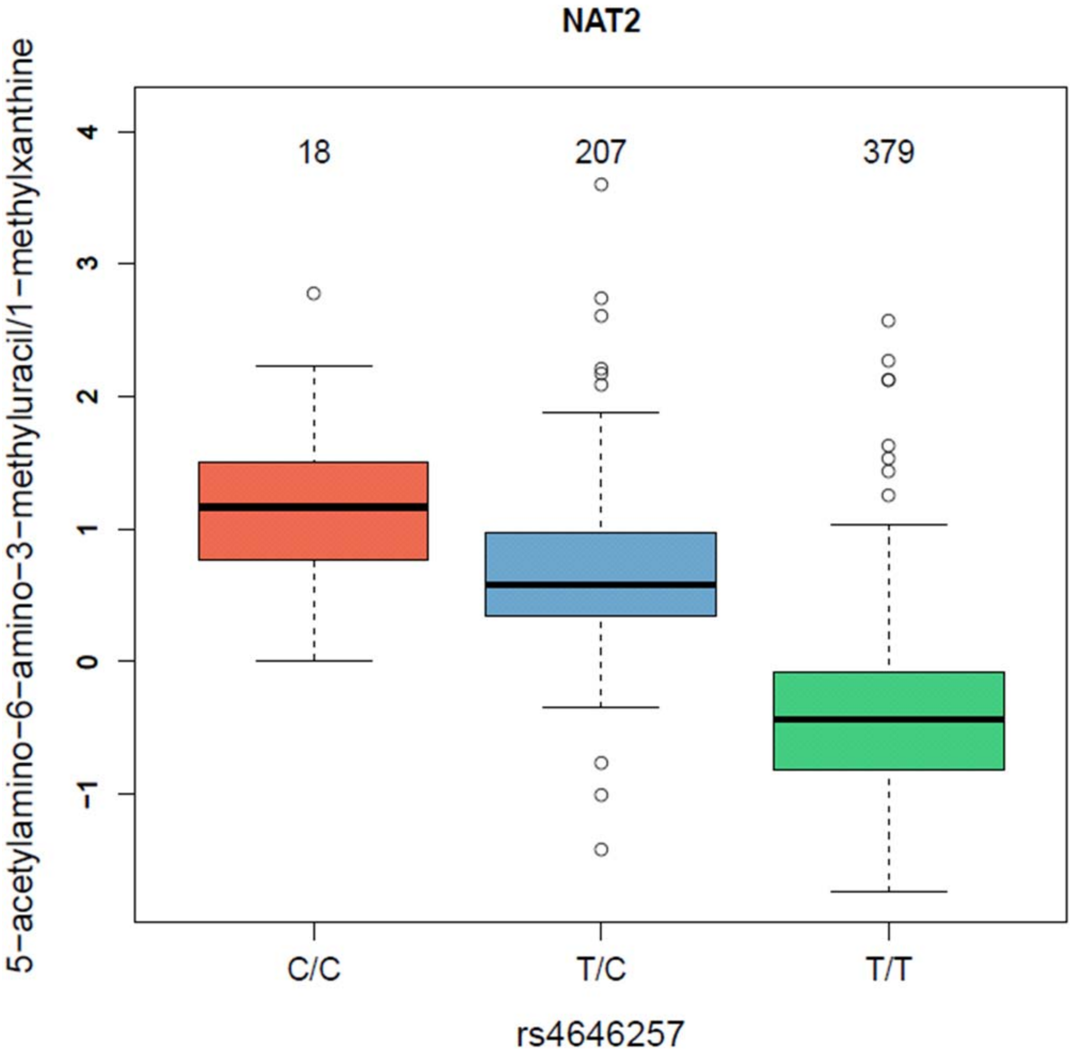

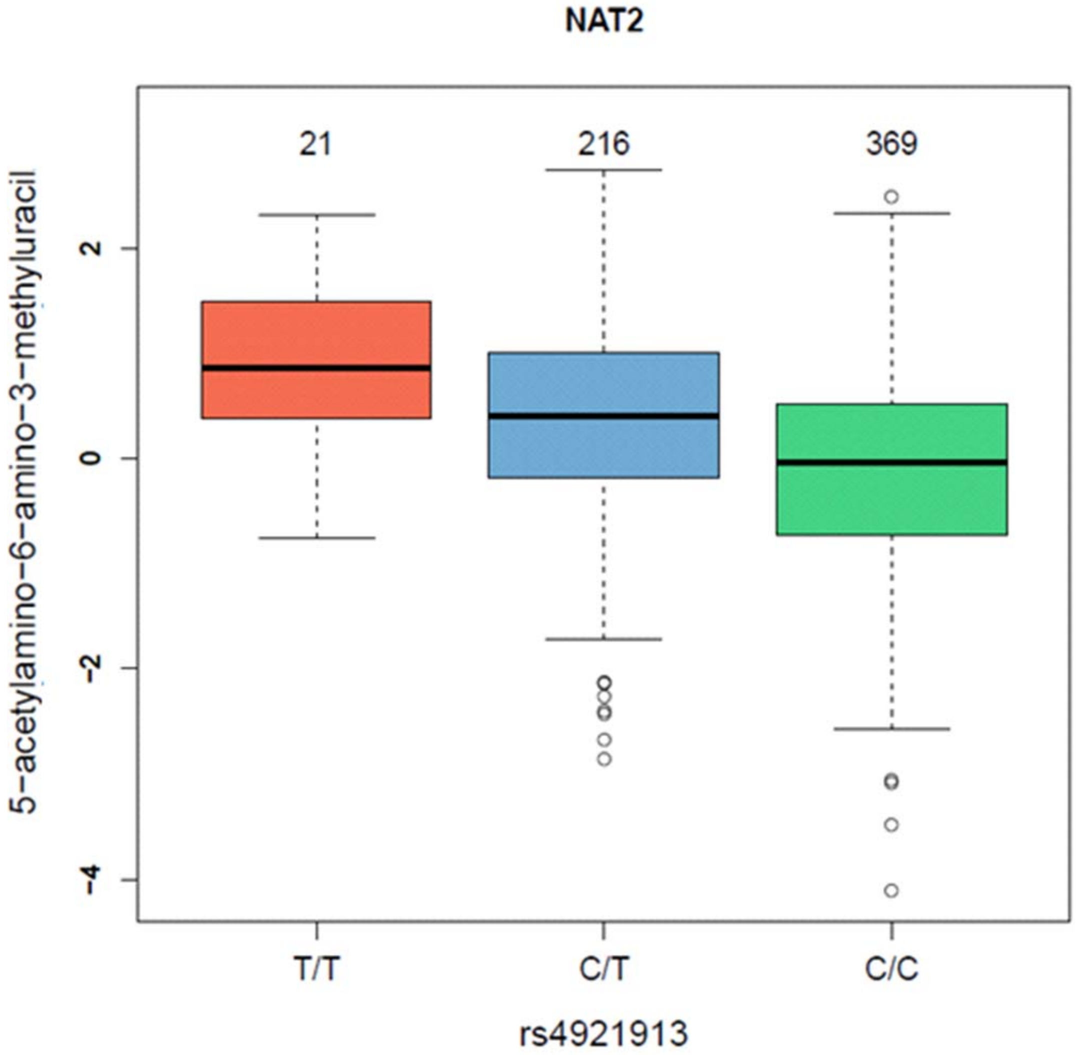
NAT2
21
216
369
5−acetylamino−6−amino−3−methyluracil
2
0
−2
−4
T/T
C/T
C/C
rs4921913

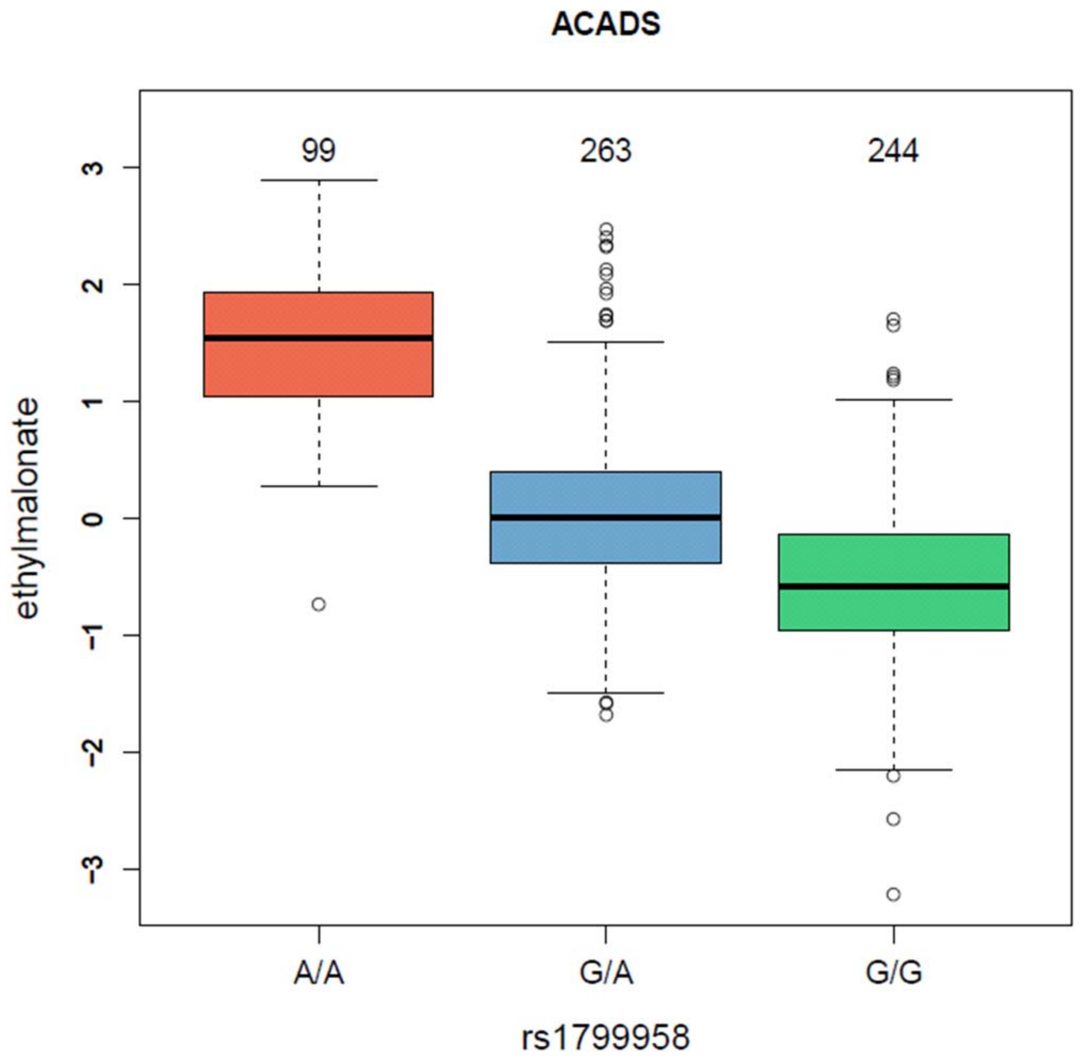
ACADS
99
263
244
3
2
1
0
-1
-2
-3
ethylmalonate
A/A
G/A
G/G
rs1799958

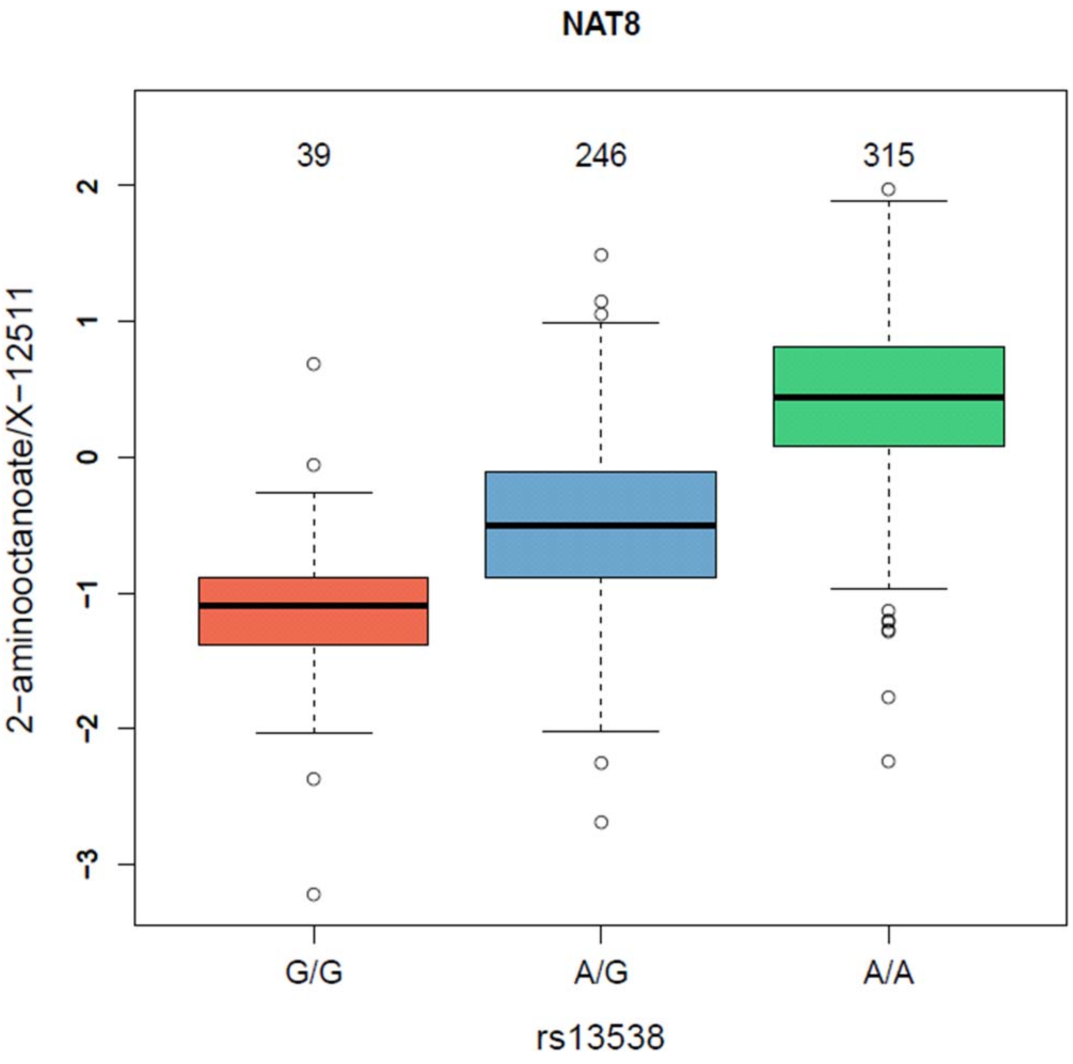
NAT8
39
246
315
2
1
0
−1
−2
−3
2−aminooctanoate/X−12511
G/G
A/G
A/A
rs13538

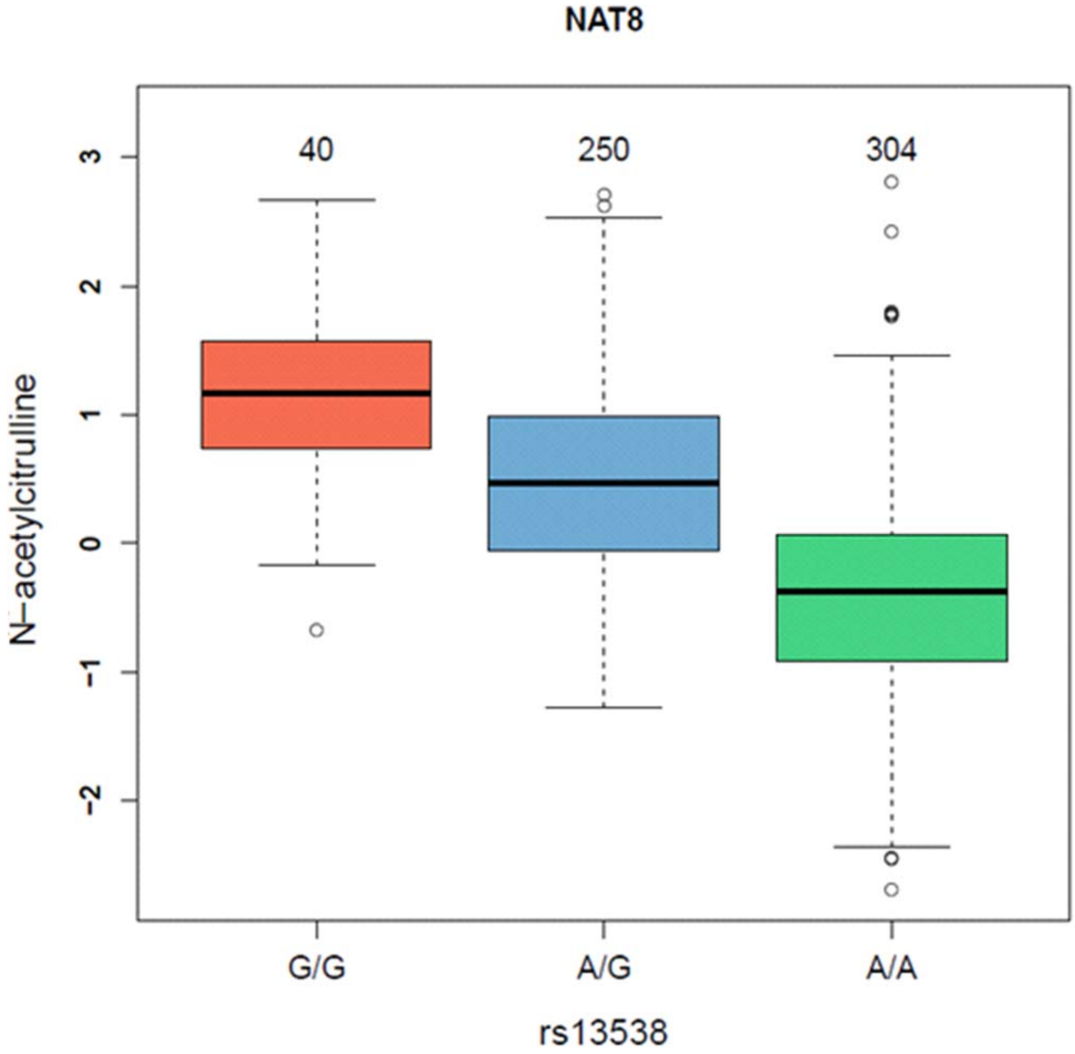
NAT8
40
250
304
N-acetylcitrulline
3
2
1
0
-1
-2
G/G
A/G
A/A
rs13538

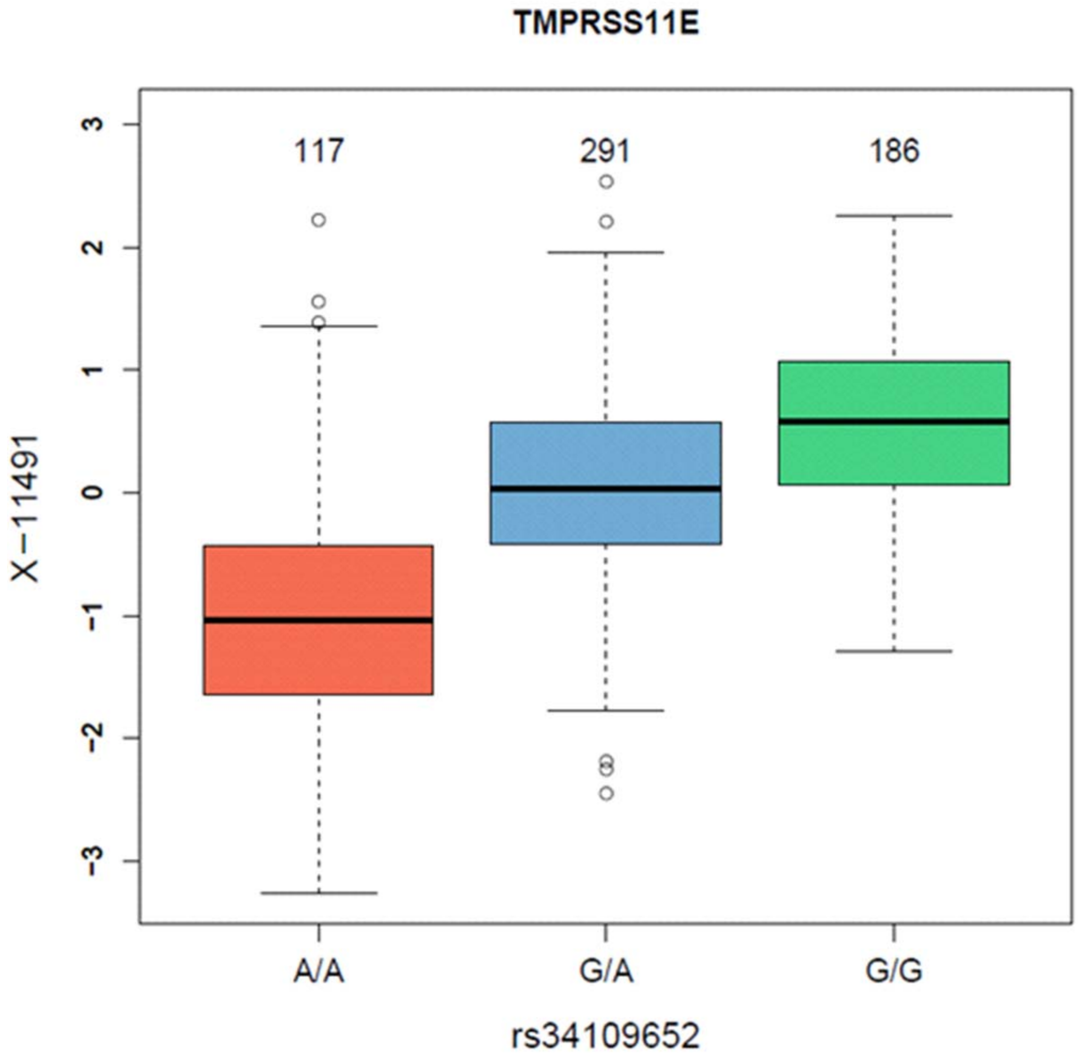

TMPRSS11E
117
291
186
3
2
1
0
-1
-2
-3
X−11491
A/A
G/A
G/G
rs34109652

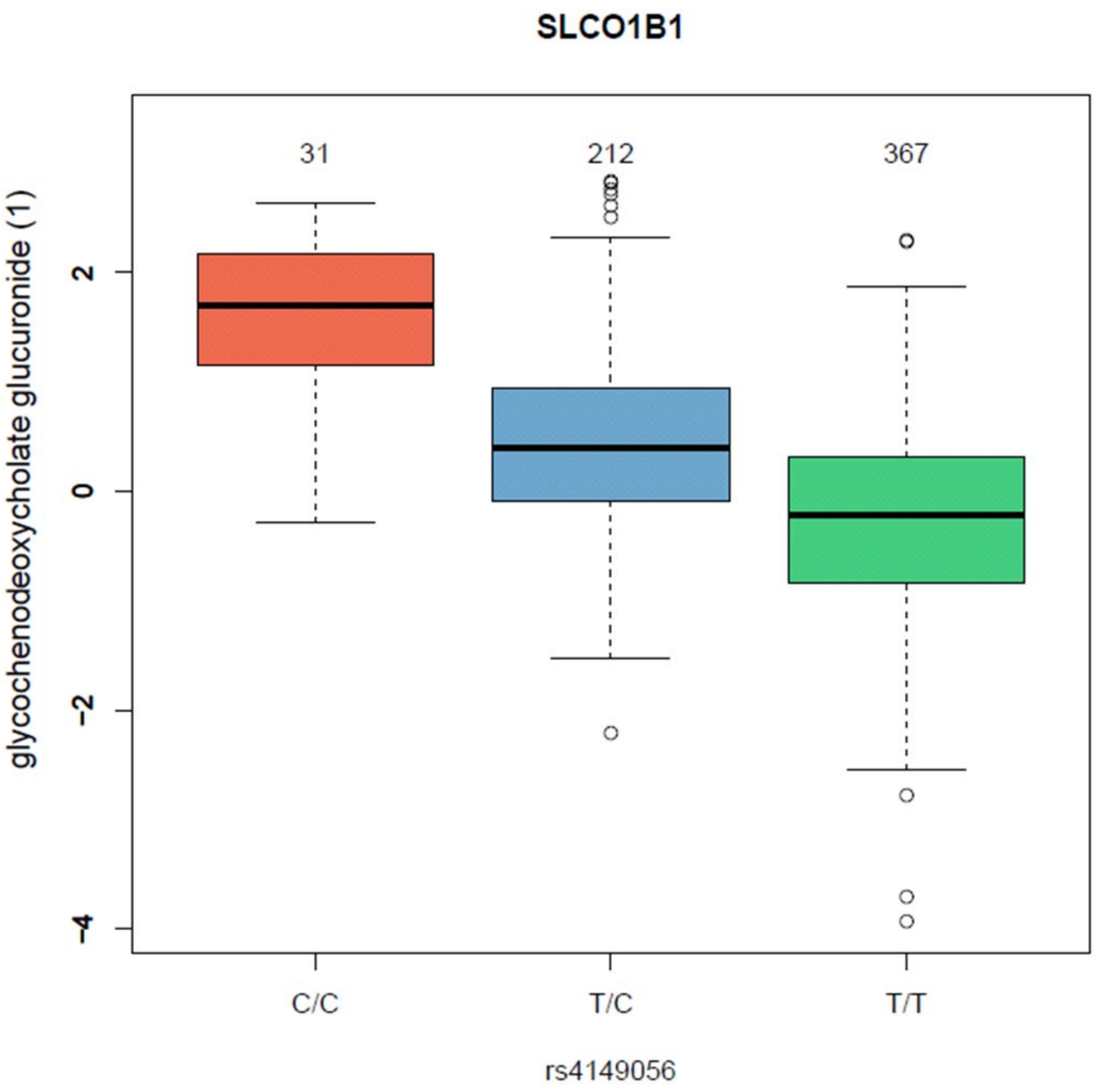
SLCO1B1
31
212
367
glycochenodeoxycholate glucuronide (1)
2
0
-2
-4
C/C
T/C
T/T
rs4149056

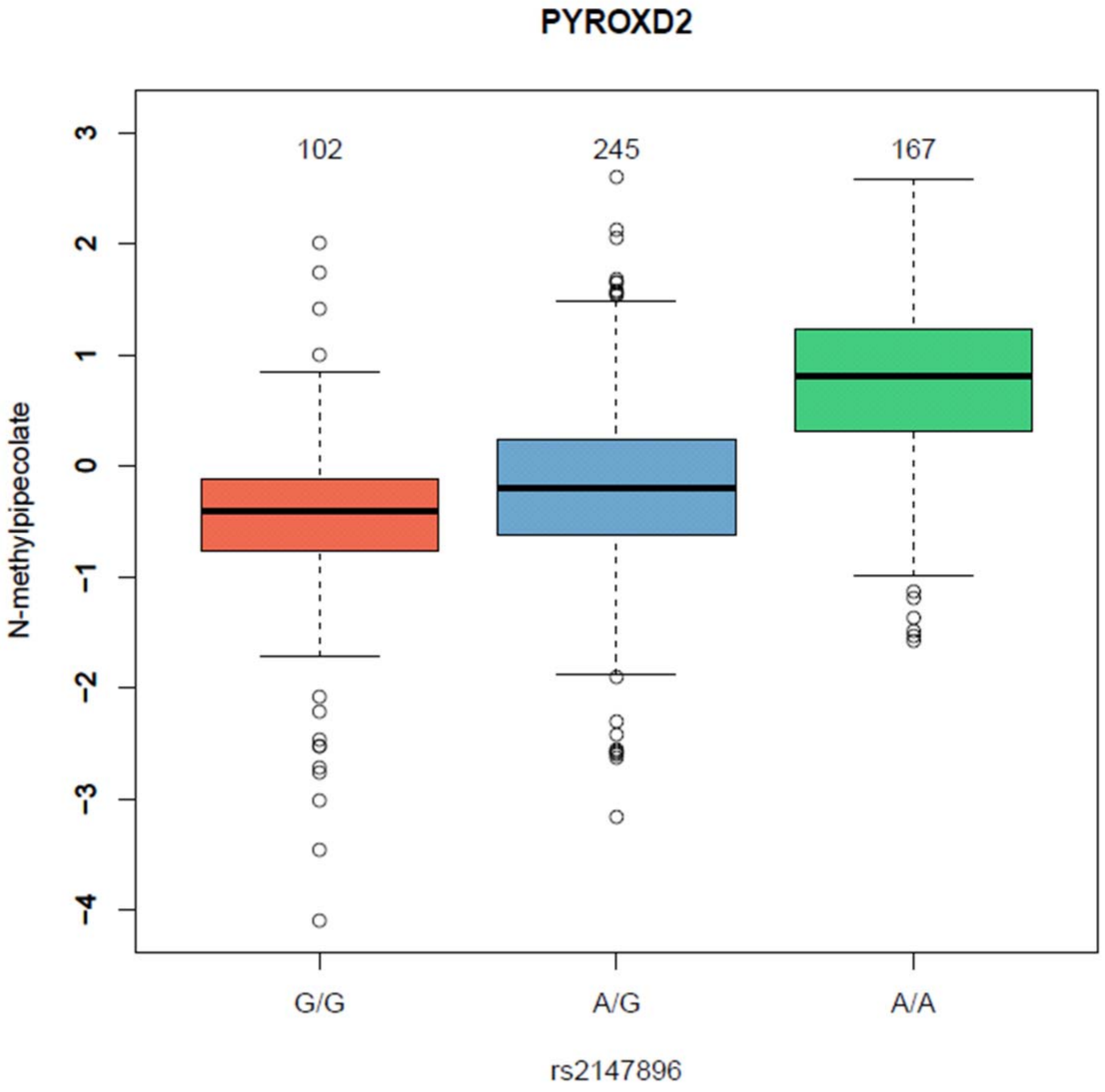
PYROXD2
N-methylpipecolate
3
2
1
0
−1
−2
−3
−4
102
245
167
G/G
A/G
A/A
rs2147896

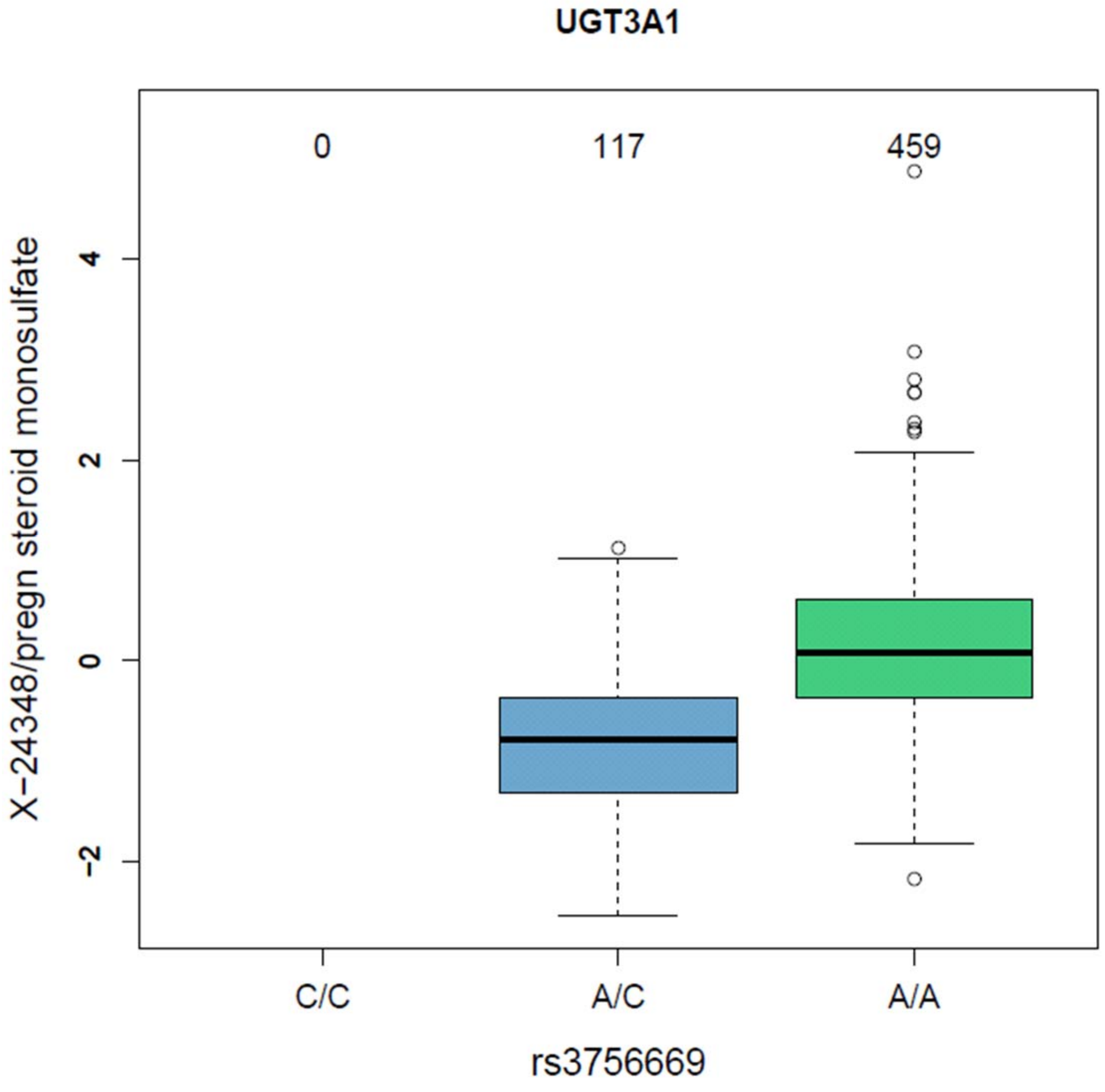
UGT3A1
0
117
459
X−24348/pregn steroid monosulfate
4
2
0
-2
C/C
A/C
A/A
rs3756669

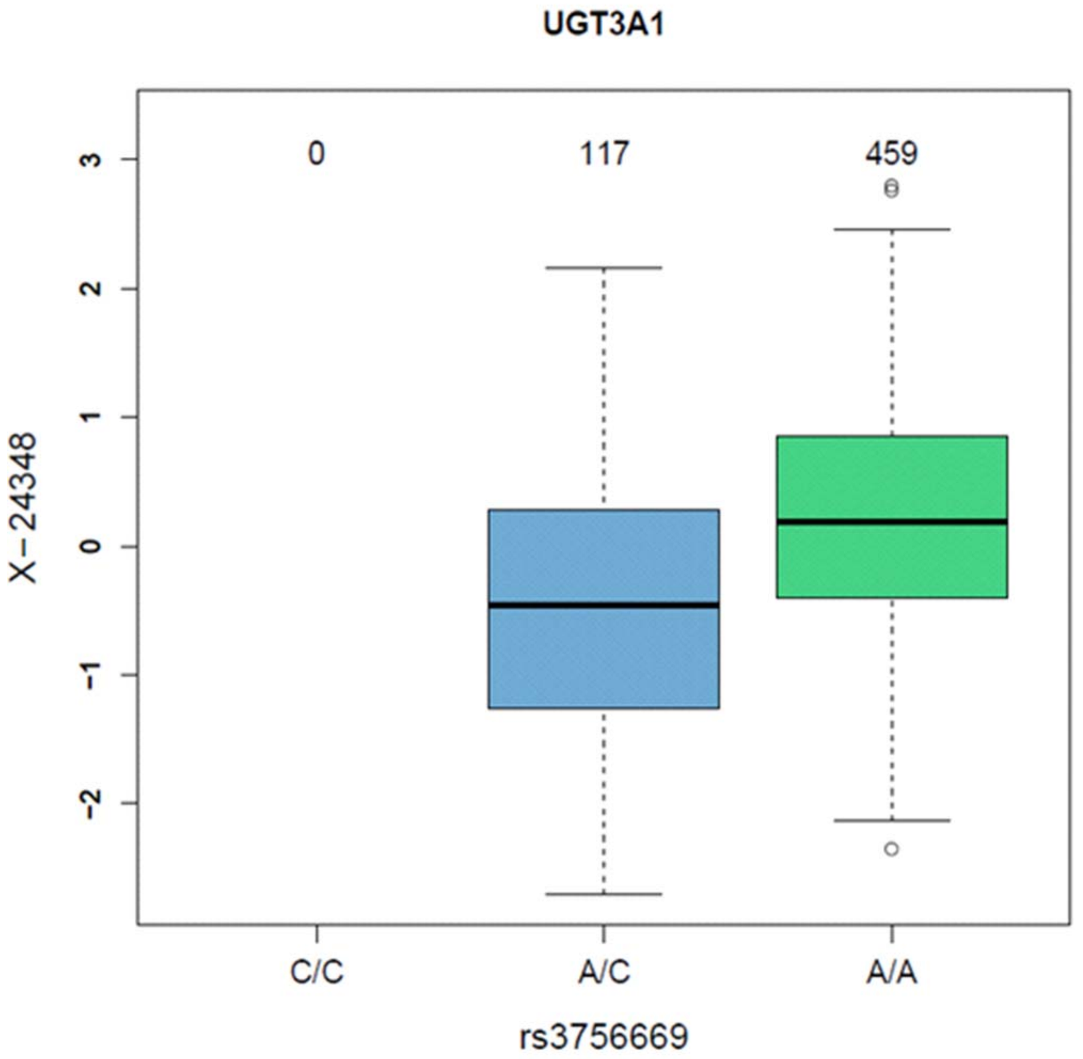
UGT3A1
0
117
459
X − 24348
3
2
1
0
-1
-2
C/C
A/C
A/A
rs3756669

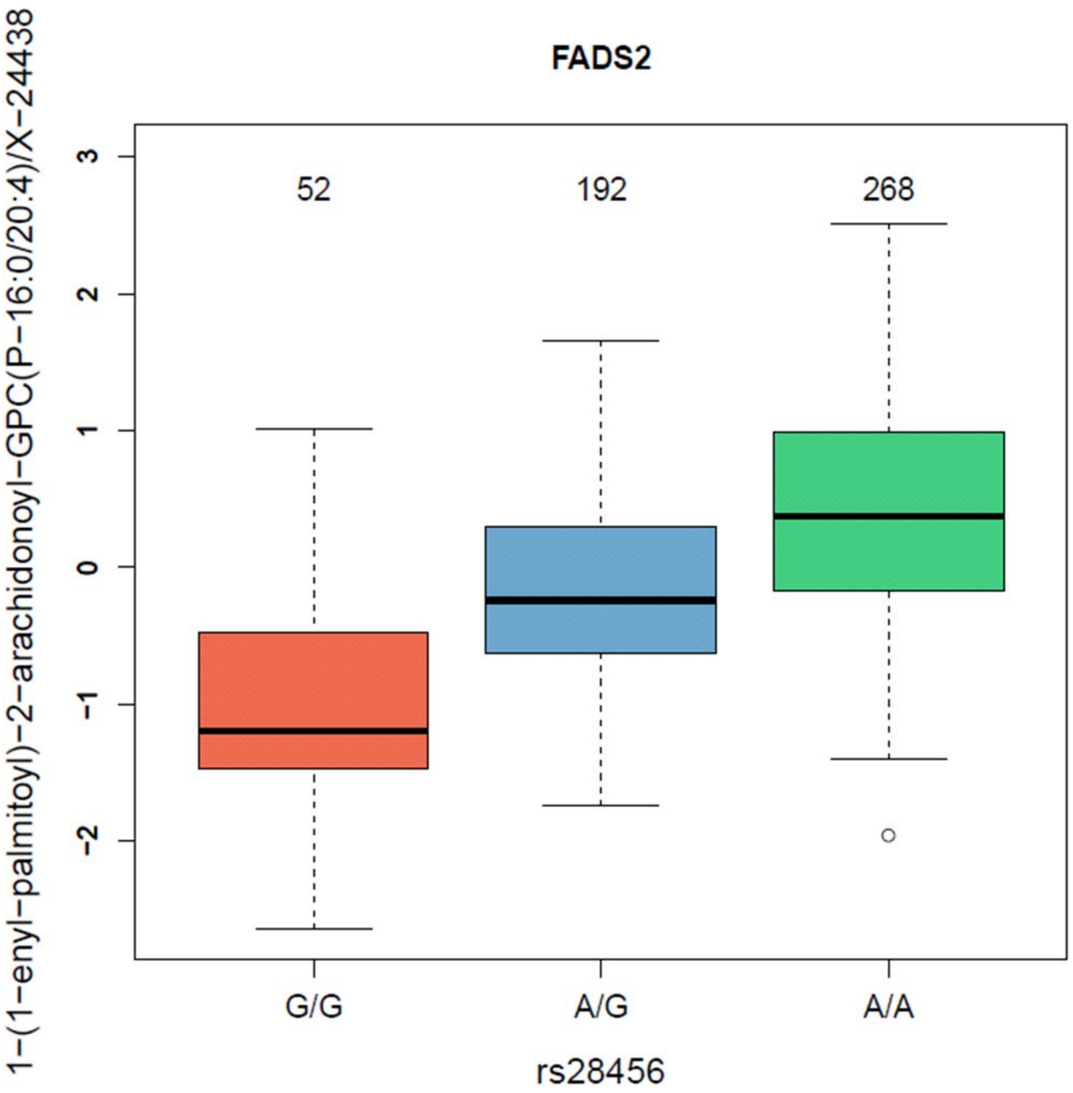
FADS2
52
192
268
3
2
1
0
-1
-2
G/G
A/G
A/A
rs28456
1−(1−enyl−palmitoyl)−2−arachidonoyl−GPC(P−16:0/20:4)/X−24438

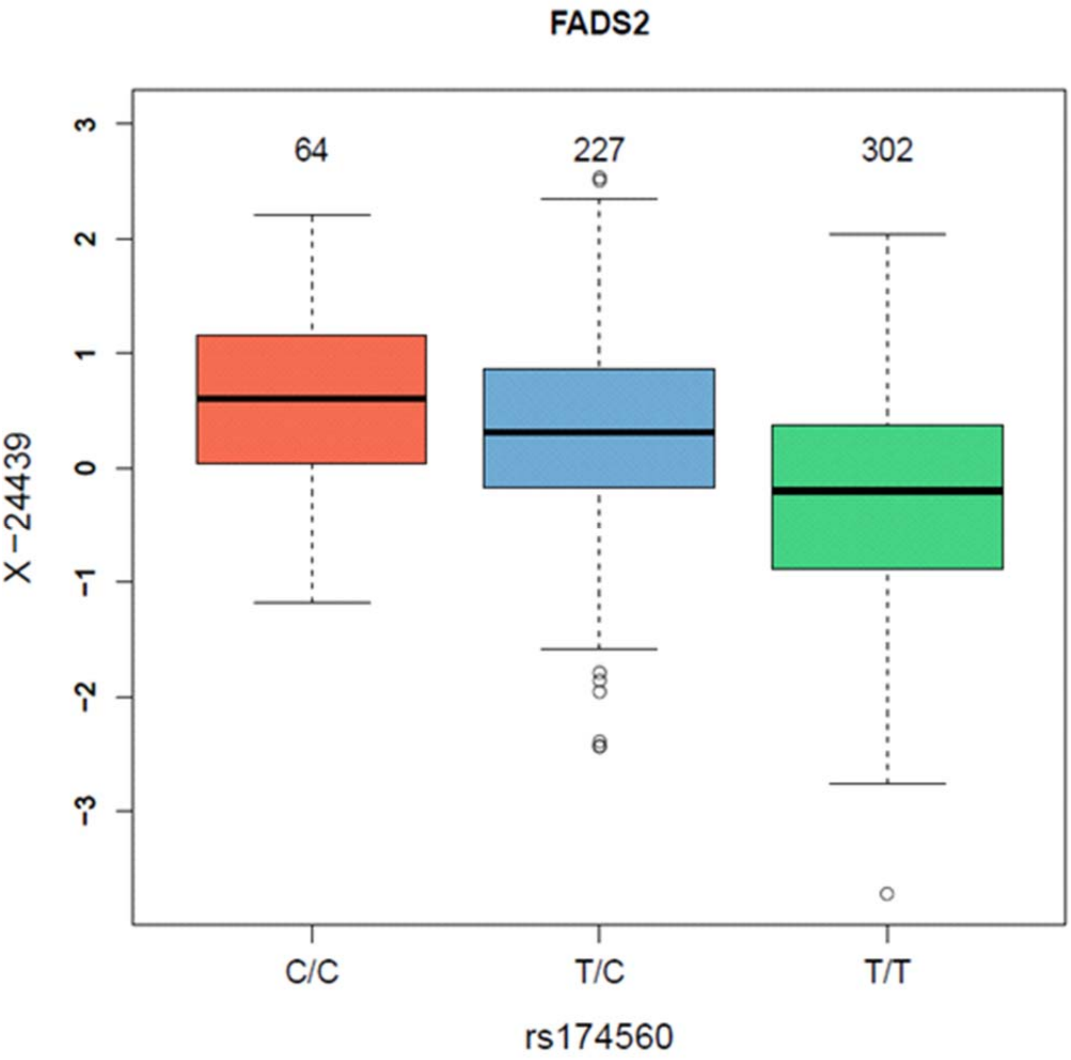

FADS2
64
227
302
3
2
1
0
-1
-2
-3
X -24439
C/C
T/C
T/T
rs174560

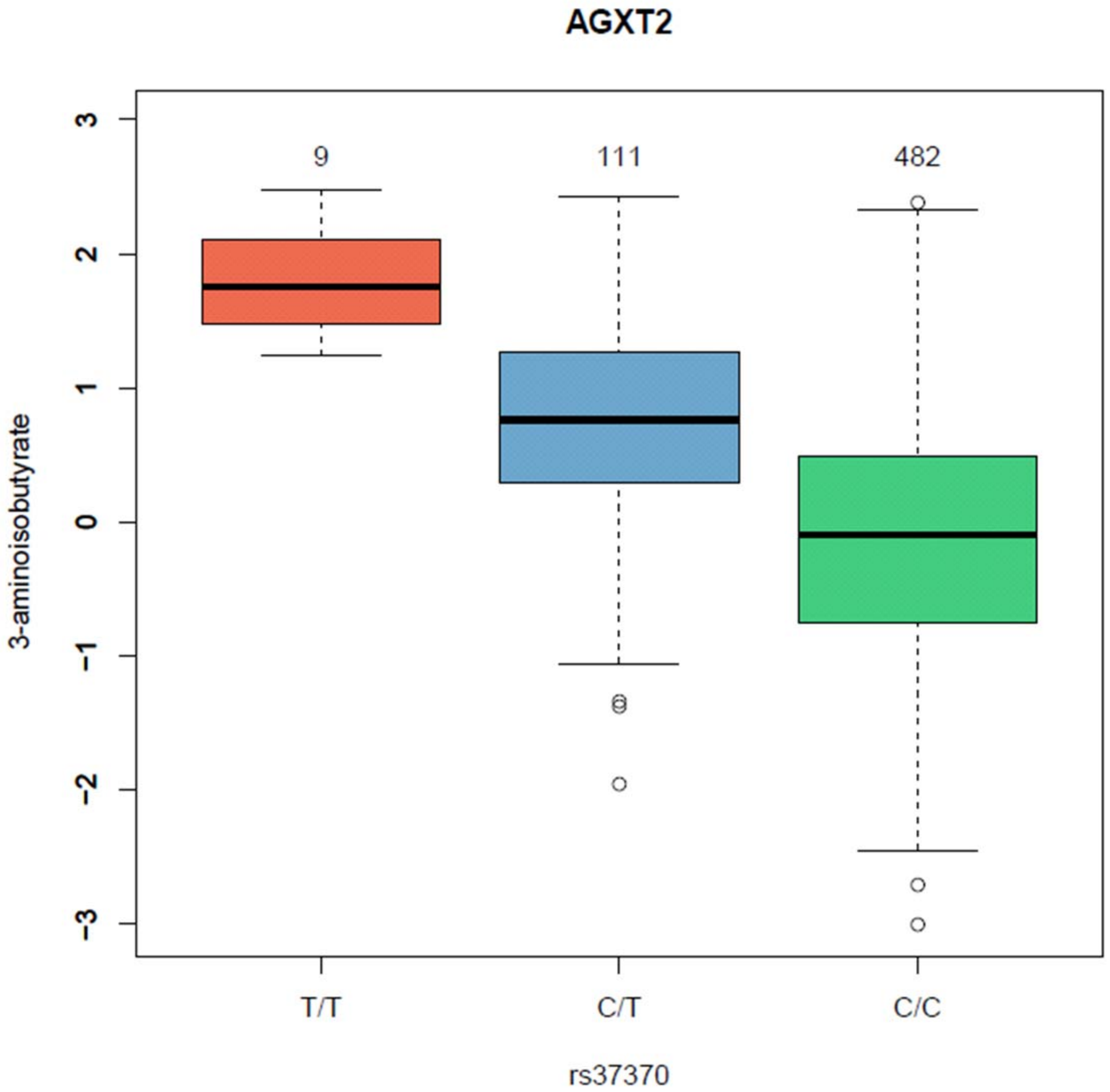
AGXT2
3-aminoisobutyrate
3
2
1
0
−1
−2
−3
9
111
482
T/T
C/T
C/C
rs37370

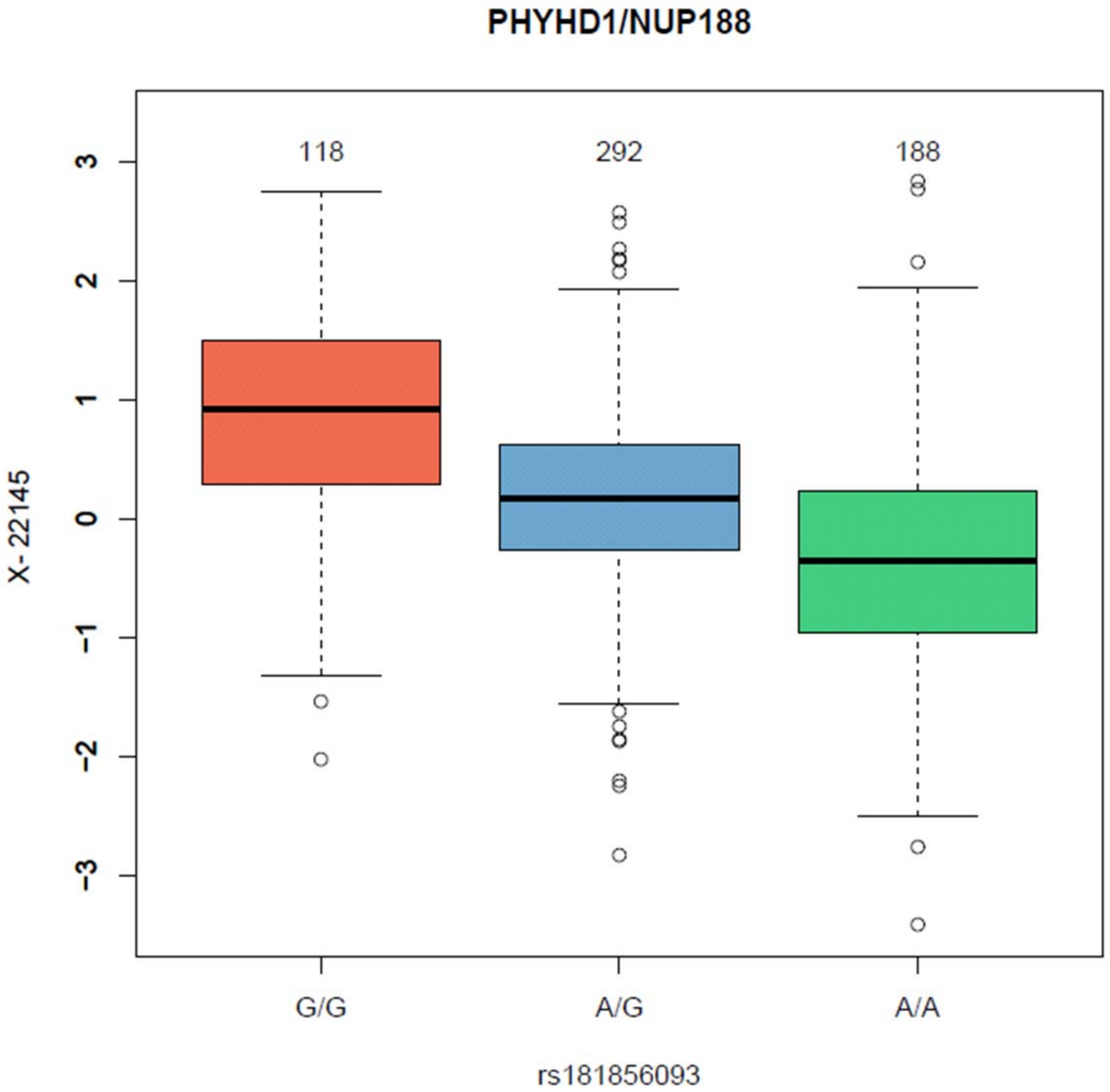
PHYHD1/NUP188
118
292
188
3
2
1
0
−1
−2
−3
X- 22145
G/G
A/G
A/A
rs181856093

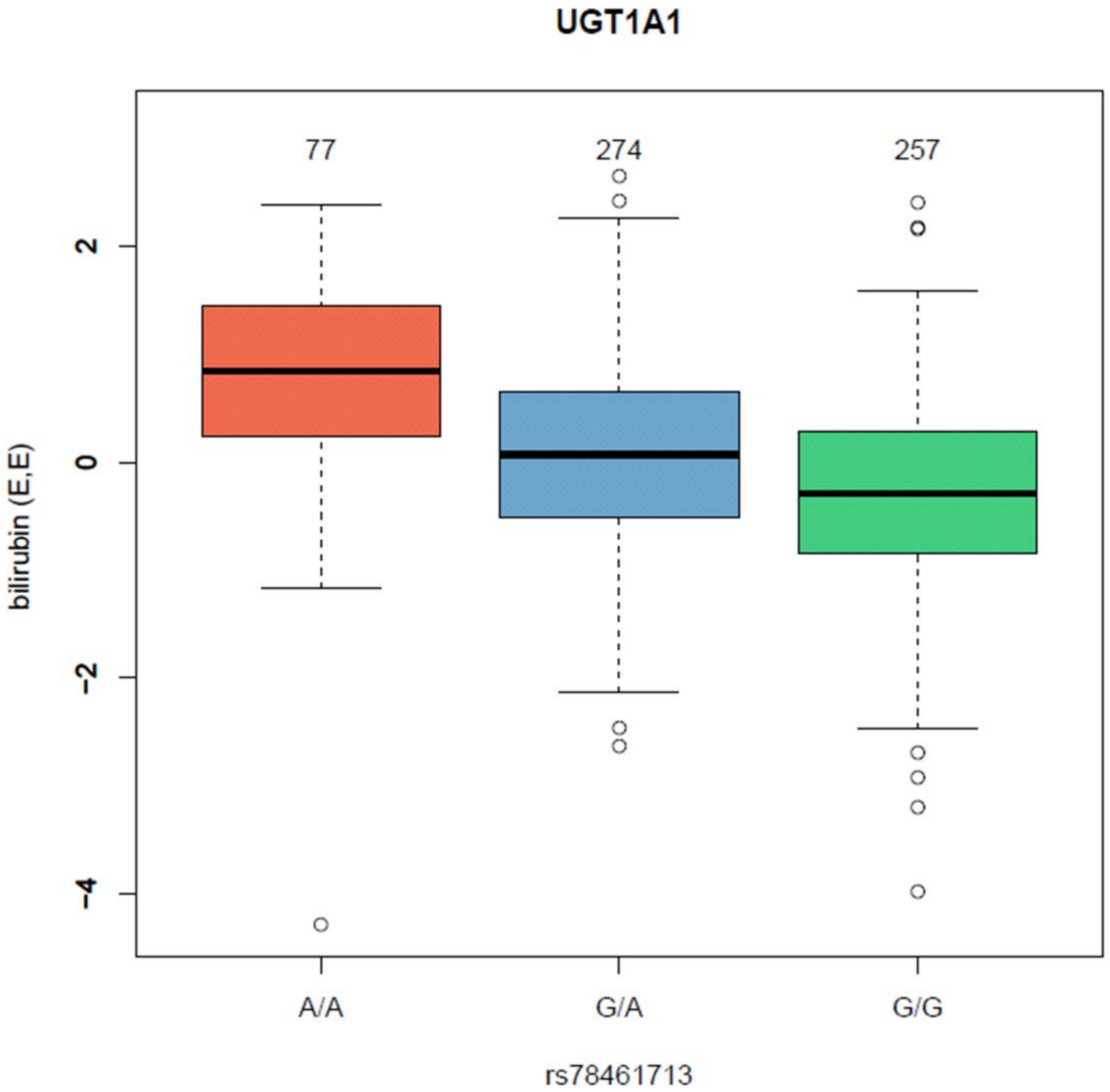

UGT1A1
77
274
257
2
0
-2
-4
bilirubin (E,E)
A/A
G/A
G/G
rs78461713

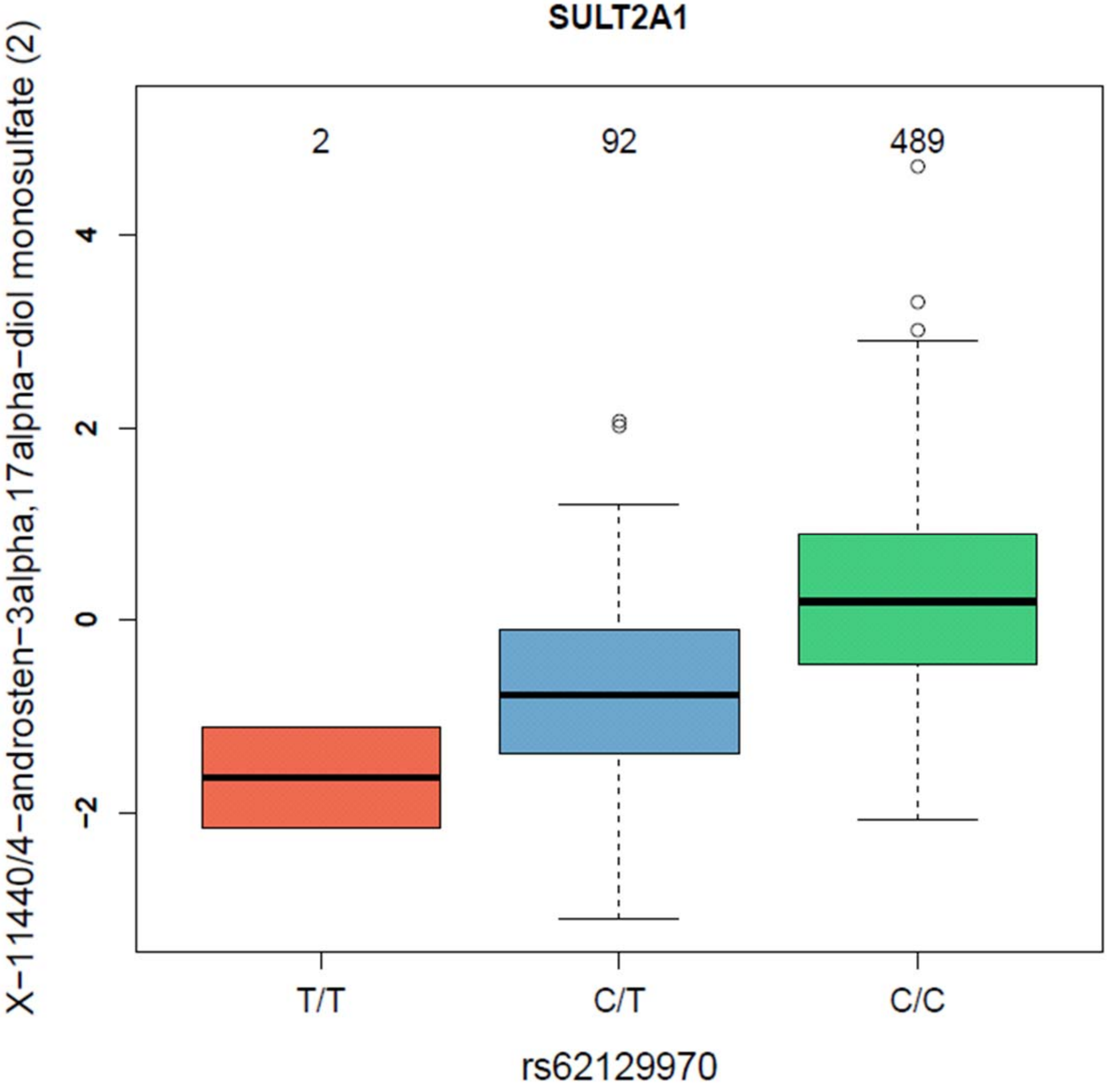
SULT2A1
2
92
489
4
2
0
-2
X−11440/4−androsten−3alpha,17alpha−diol monosulfate (2)
T/T
C/T
C/C
rs62129970

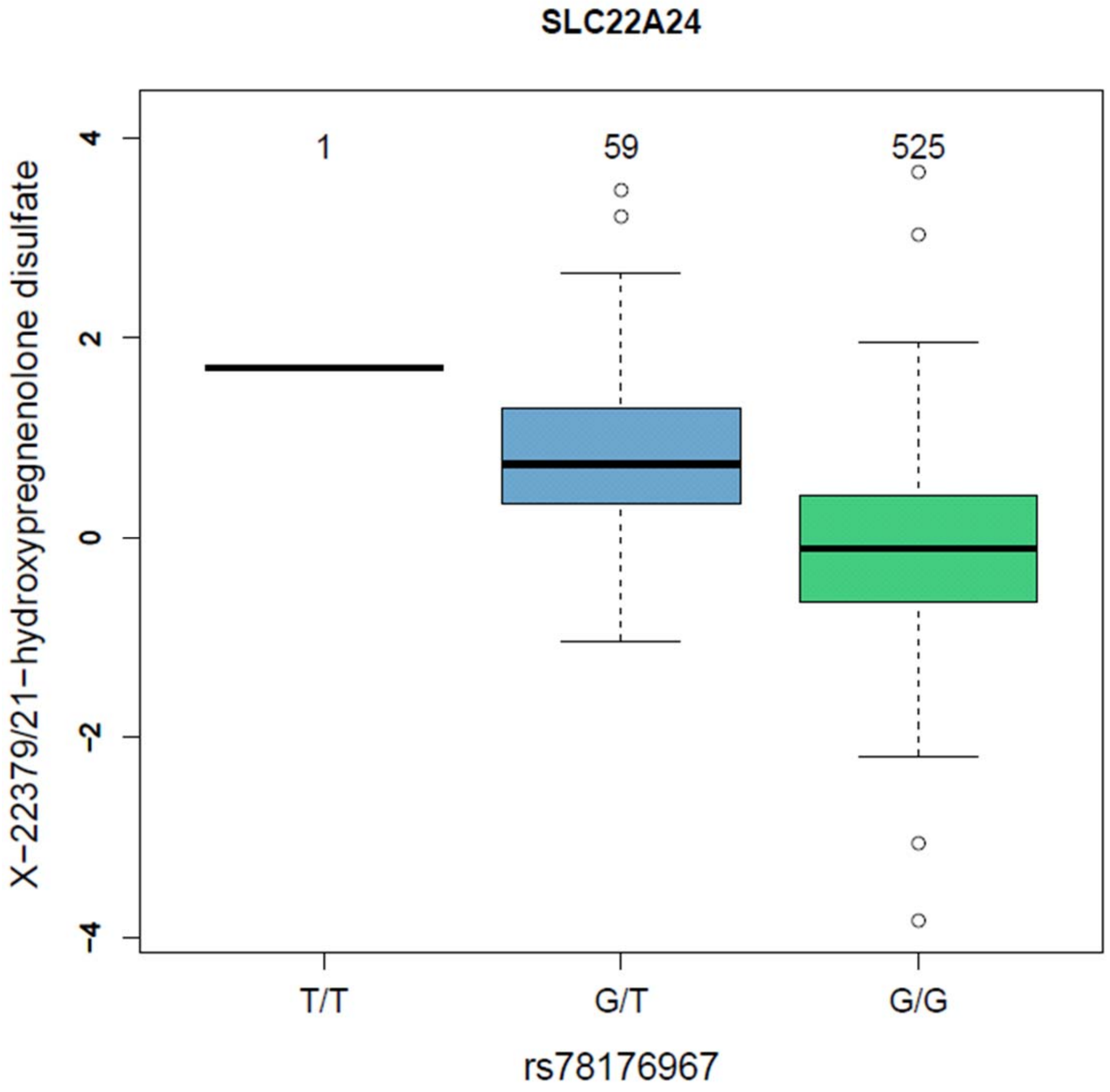
SLC22A24
X−22379/21−hydroxypregnenolone disulfate
1
59
525
4
2
0
-2
-4
T/T
G/T
G/G
rs78176967

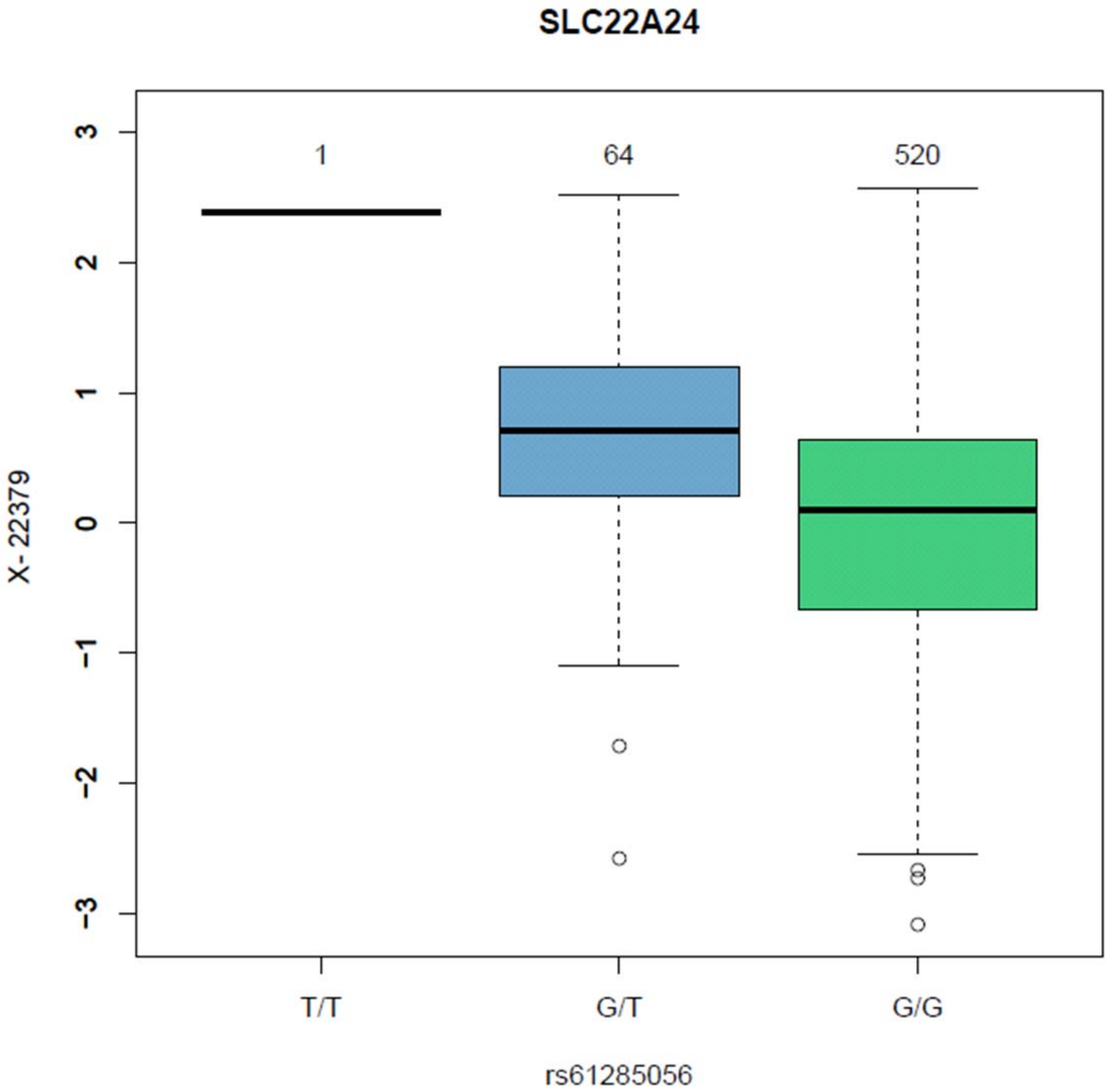

SLC22A24
1
64
520
3
2
1
0
−1
−2
−3
X- 22379
T/T
G/T
G/G
rs61285056

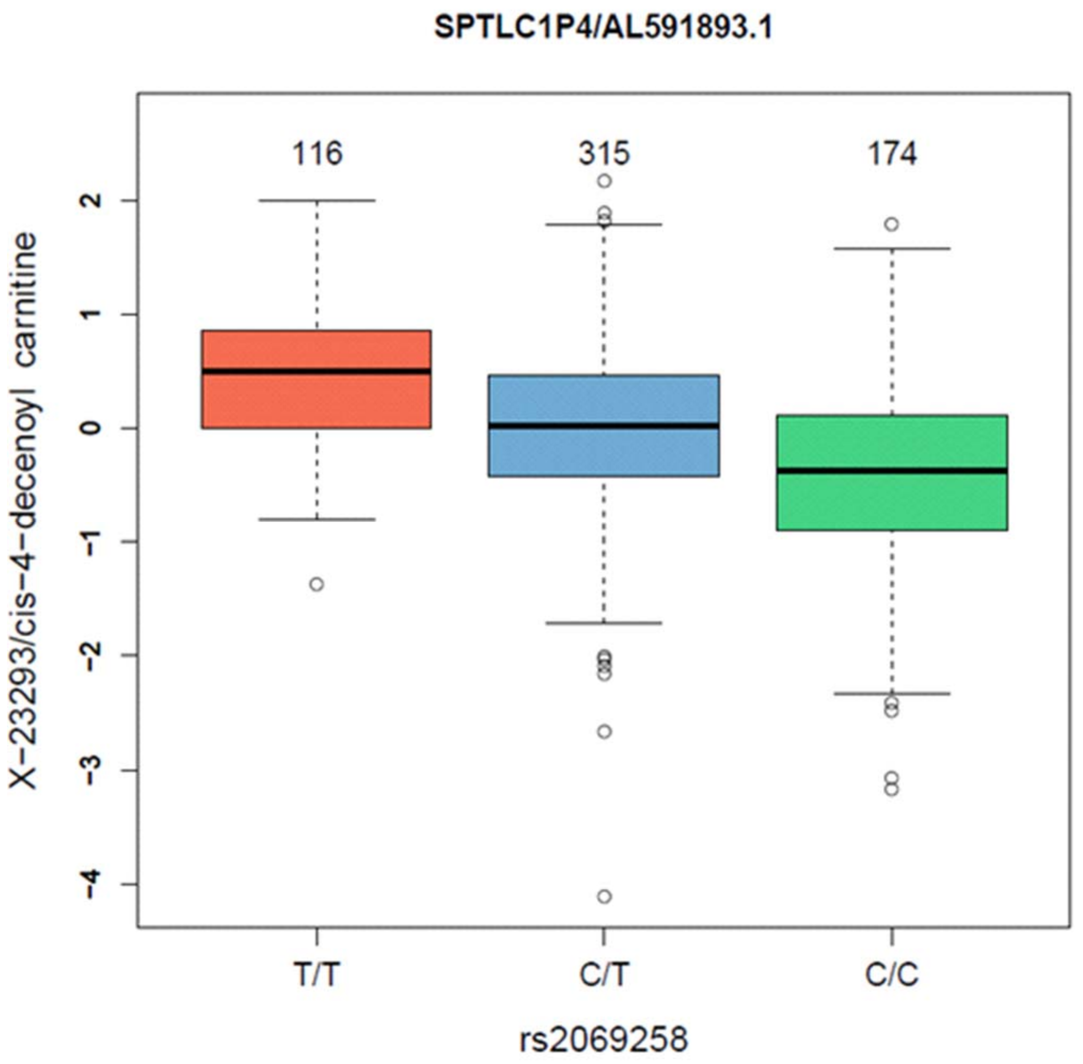

SPTLC1P4/AL591893.1
116
315
174
X−23293/cis−4−decenoyl carnitine
2
1
0
−1
−2
−3
−4
T/T
C/T
C/C
rs2069258

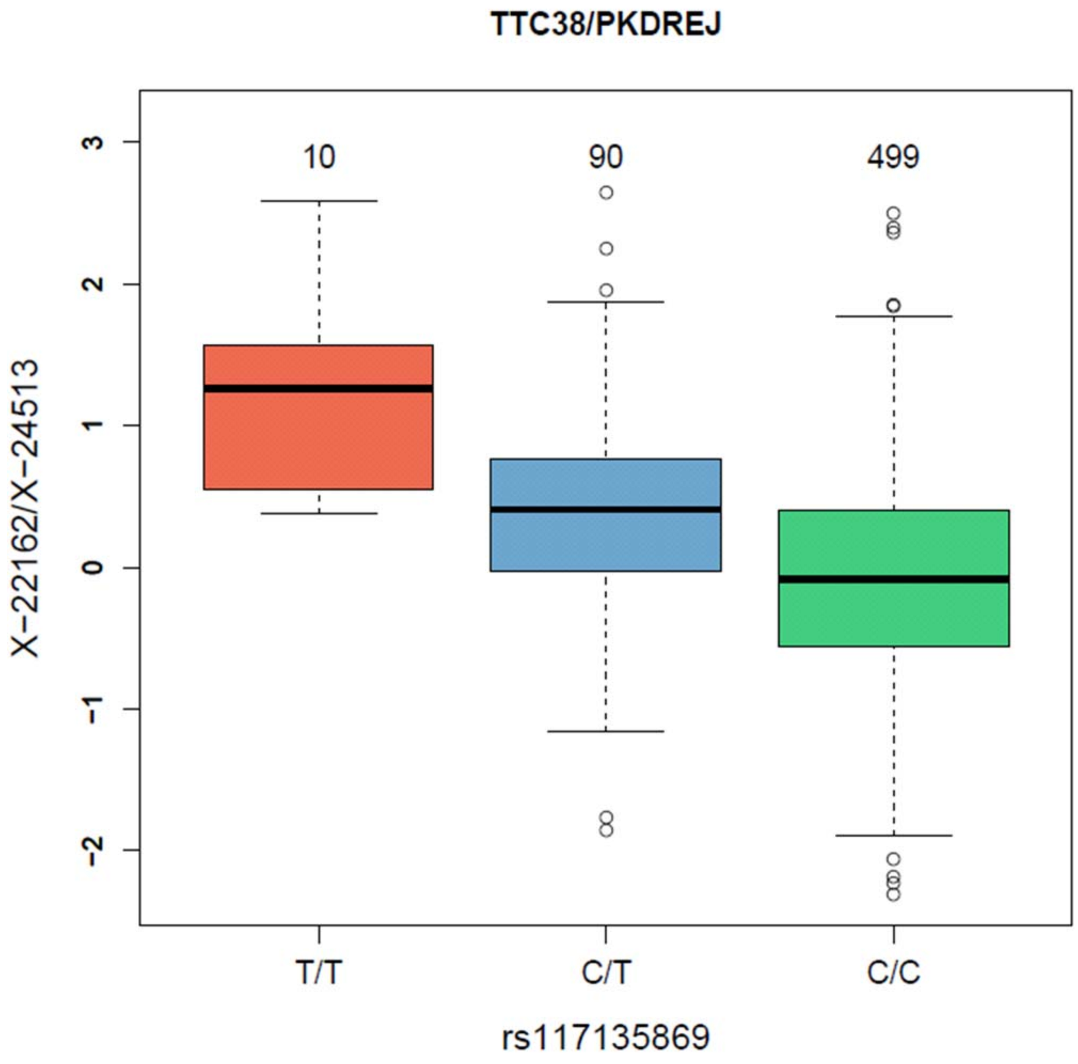
TTC38/PKDREJ
10
90
499
3
2
1
0
-1
-2
X-22162/X-24513
T/T
C/T
C/C
rs117135869

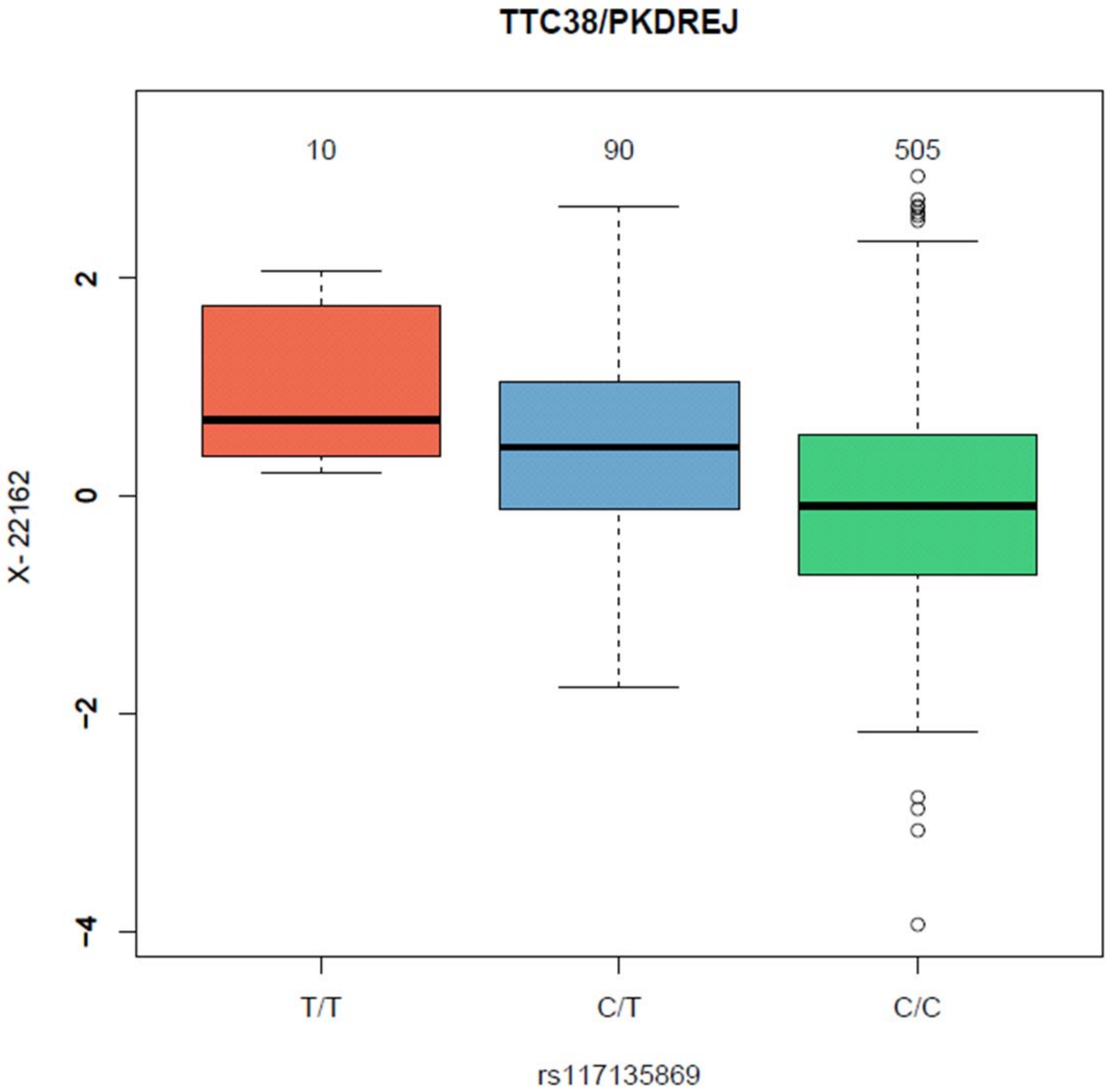

TTC38/PKDREJ
10
90
505
2
0
-2
-4
X- 22162
T/T
C/T
C/C
rs117135869

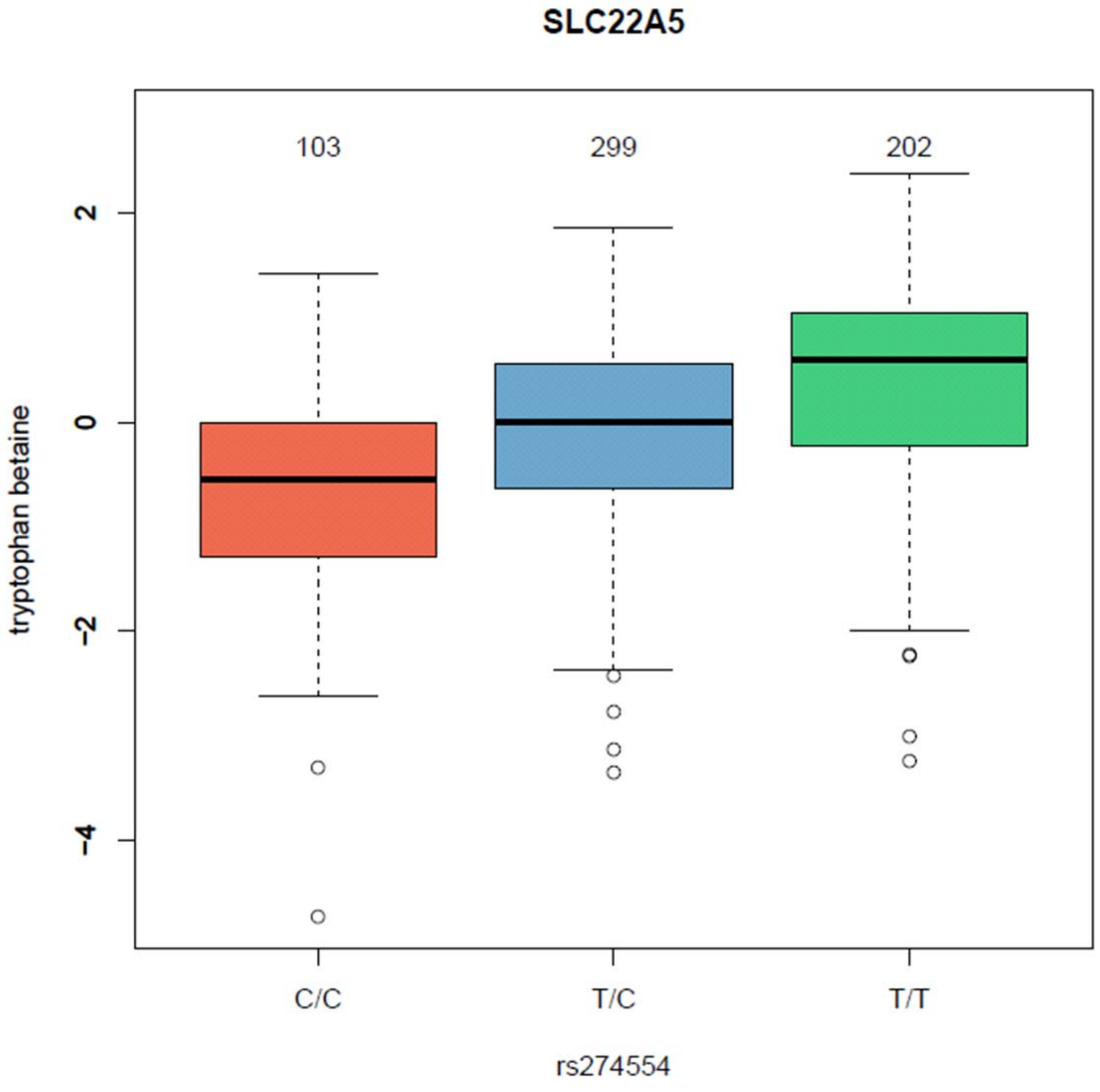

SLC22A5
103
299
202
2
0
-2
-4
tryptophan betaine
C/C
T/C
T/T
rs274554

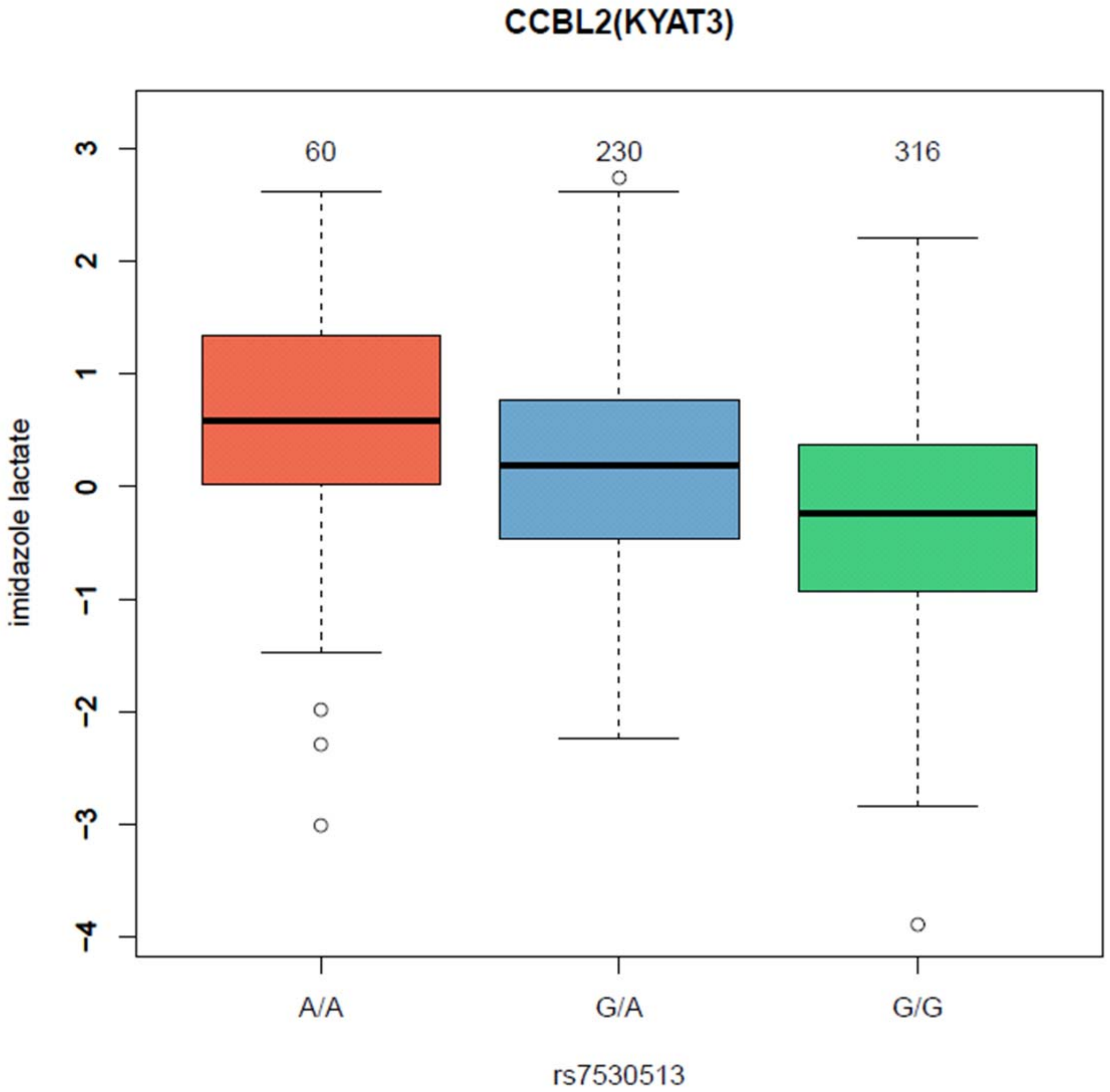
CCBL2(KYAT3)
60
230
316
3
2
1
0
−1
−2
−3
−4
imidazole lactate
A/A
G/A
G/G
rs7530513

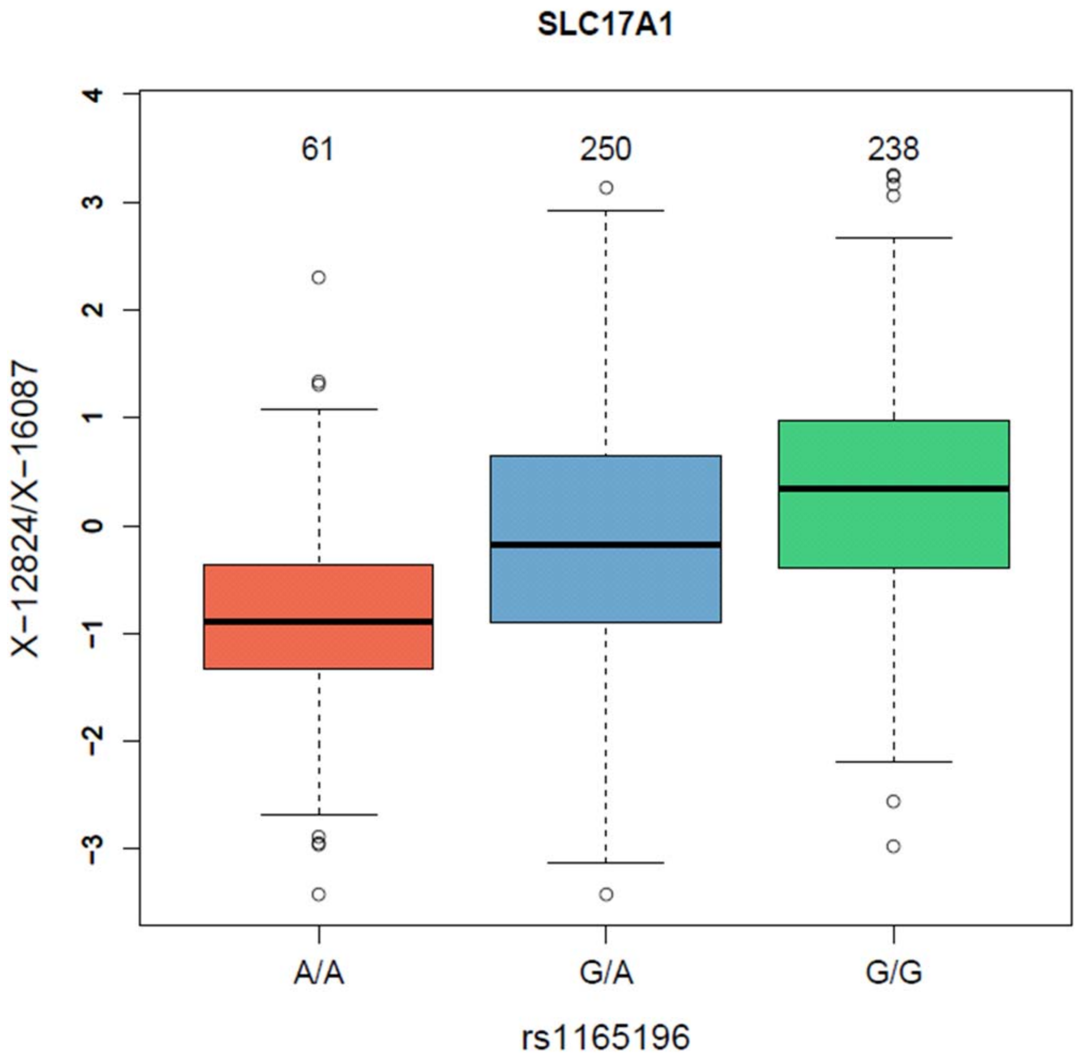
SLC17A1
61
250
238
X−12824/X−16087
4
3
2
1
0
−1
−2
−3
A/A
G/A
G/G
rs1165196

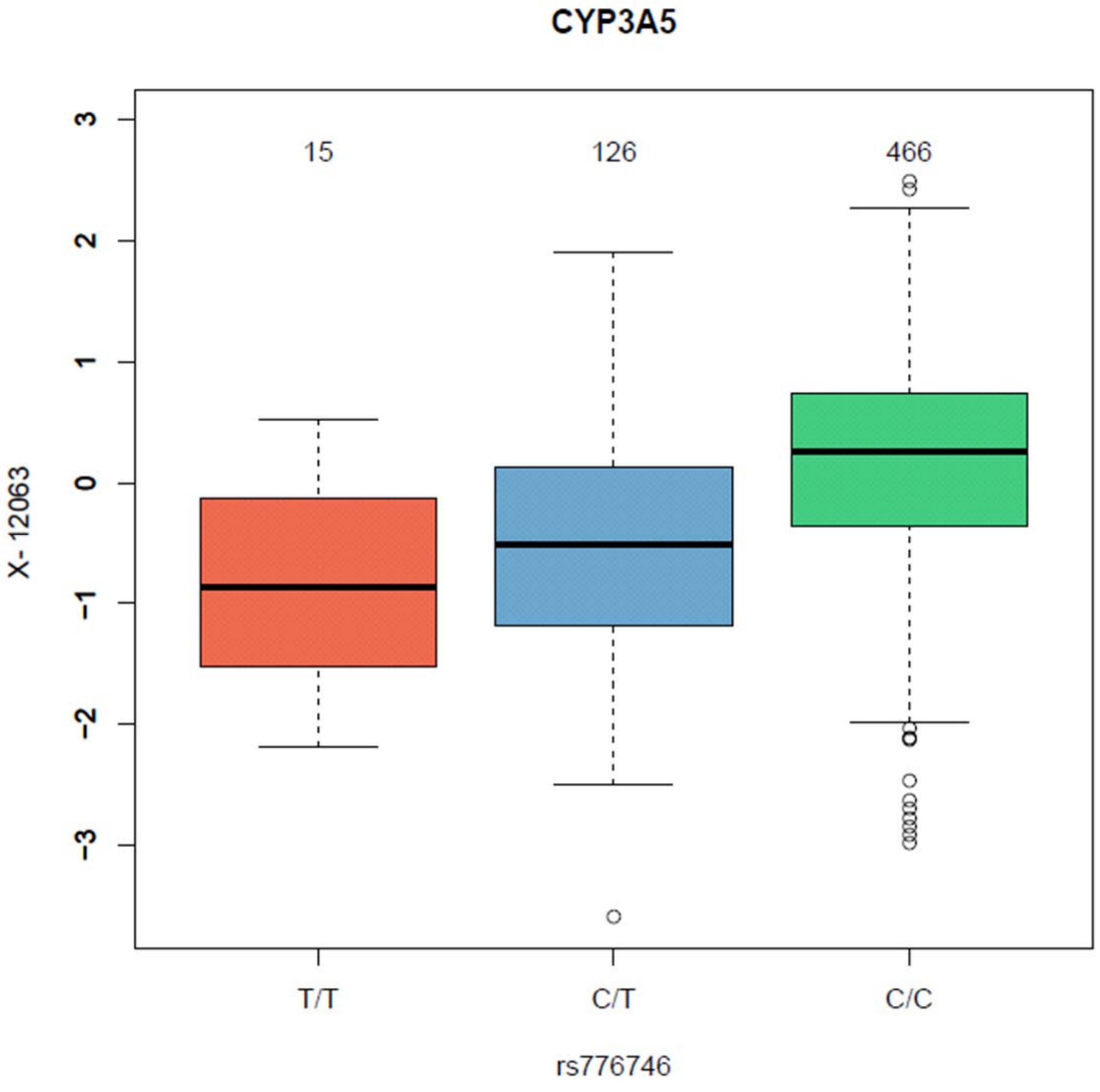
CYP3A5
X- 12063
3
2
1
0
-1
-2
-3
15
126
466
T/T
C/T
C/C
rs776746

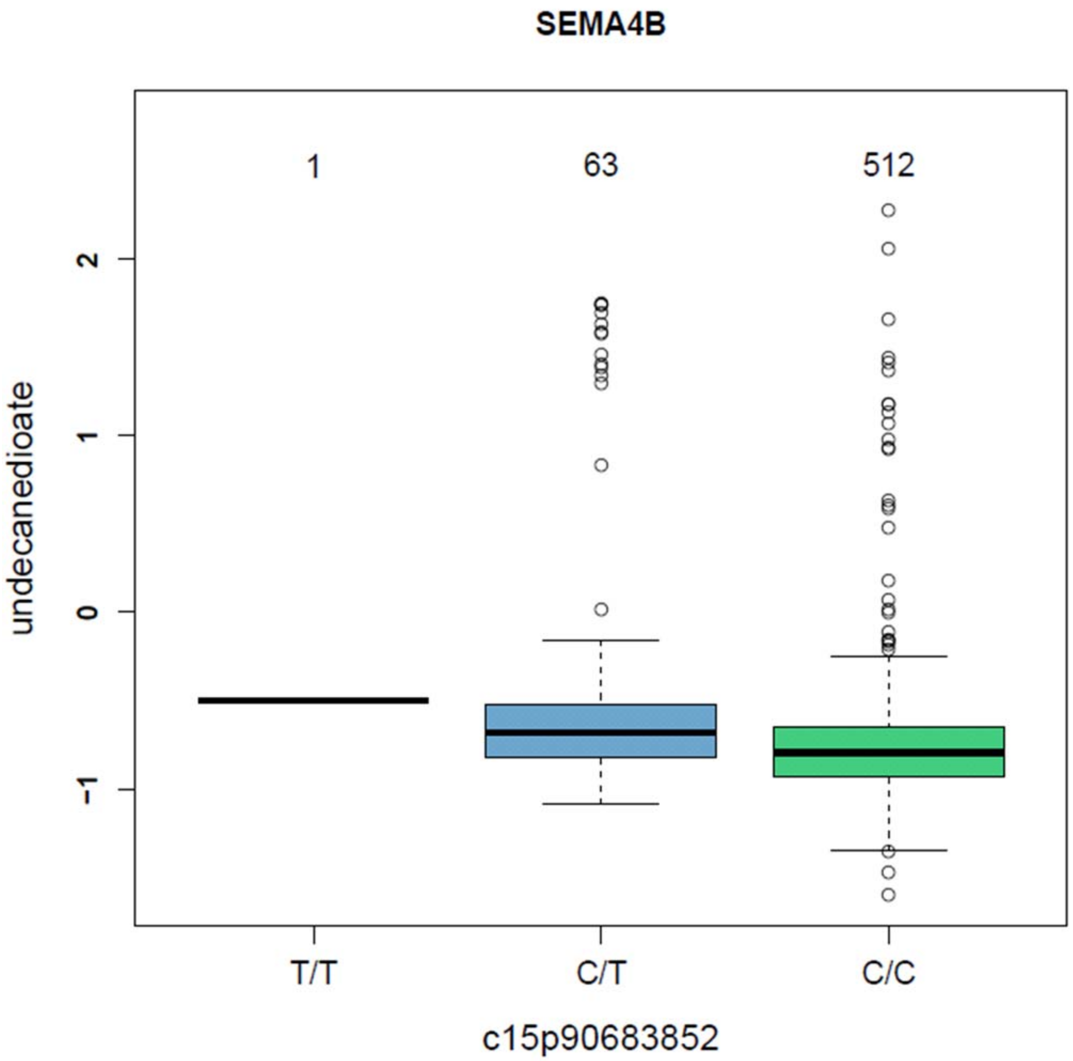
SEMA4B
1
63
512
2
1
0
-1
undecanedioate
T/T
C/T
C/C
c15p90683852

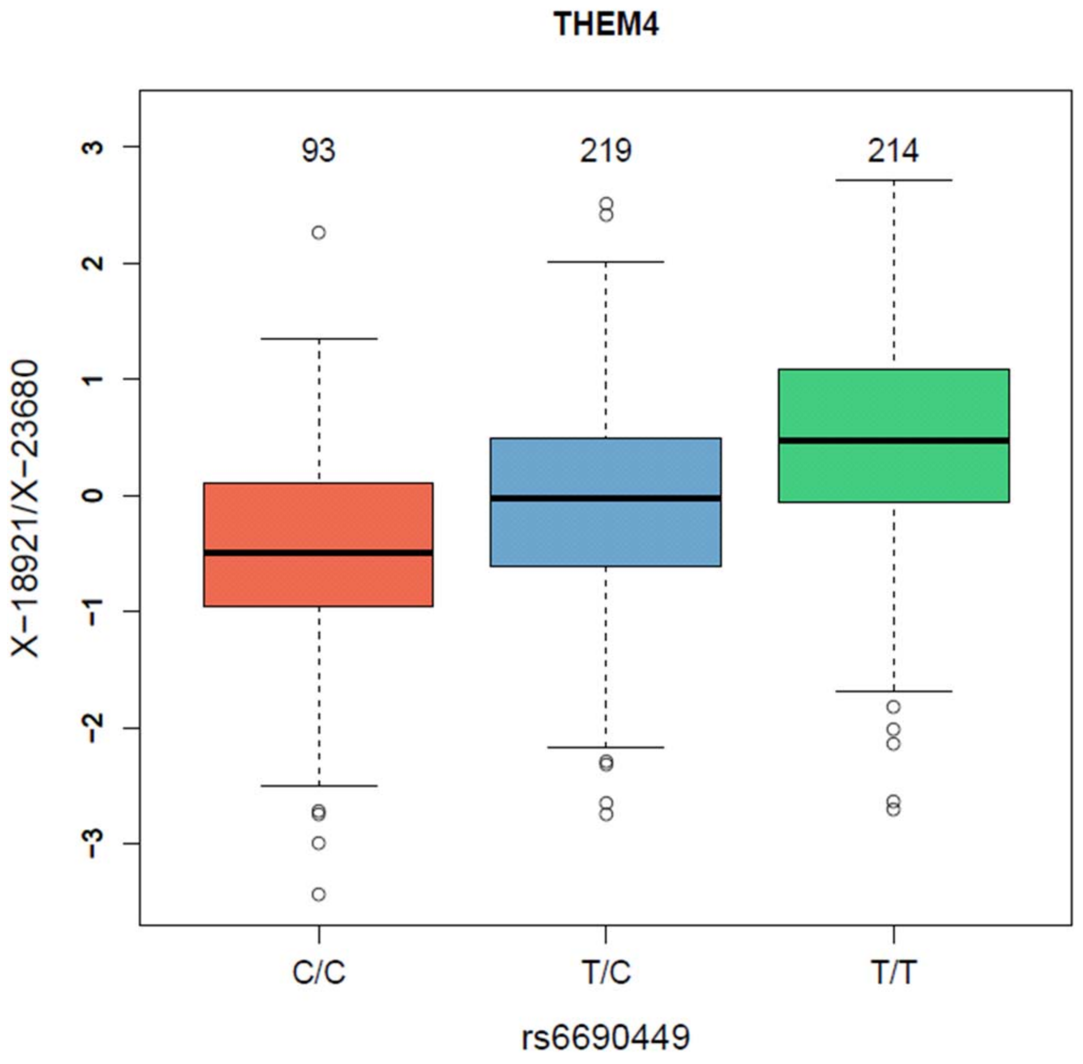

THEM4
93
219
214
X−18921/X−23680
3
2
1
0
−1
−2
−3
C/C
T/C
T/T
rs6690449

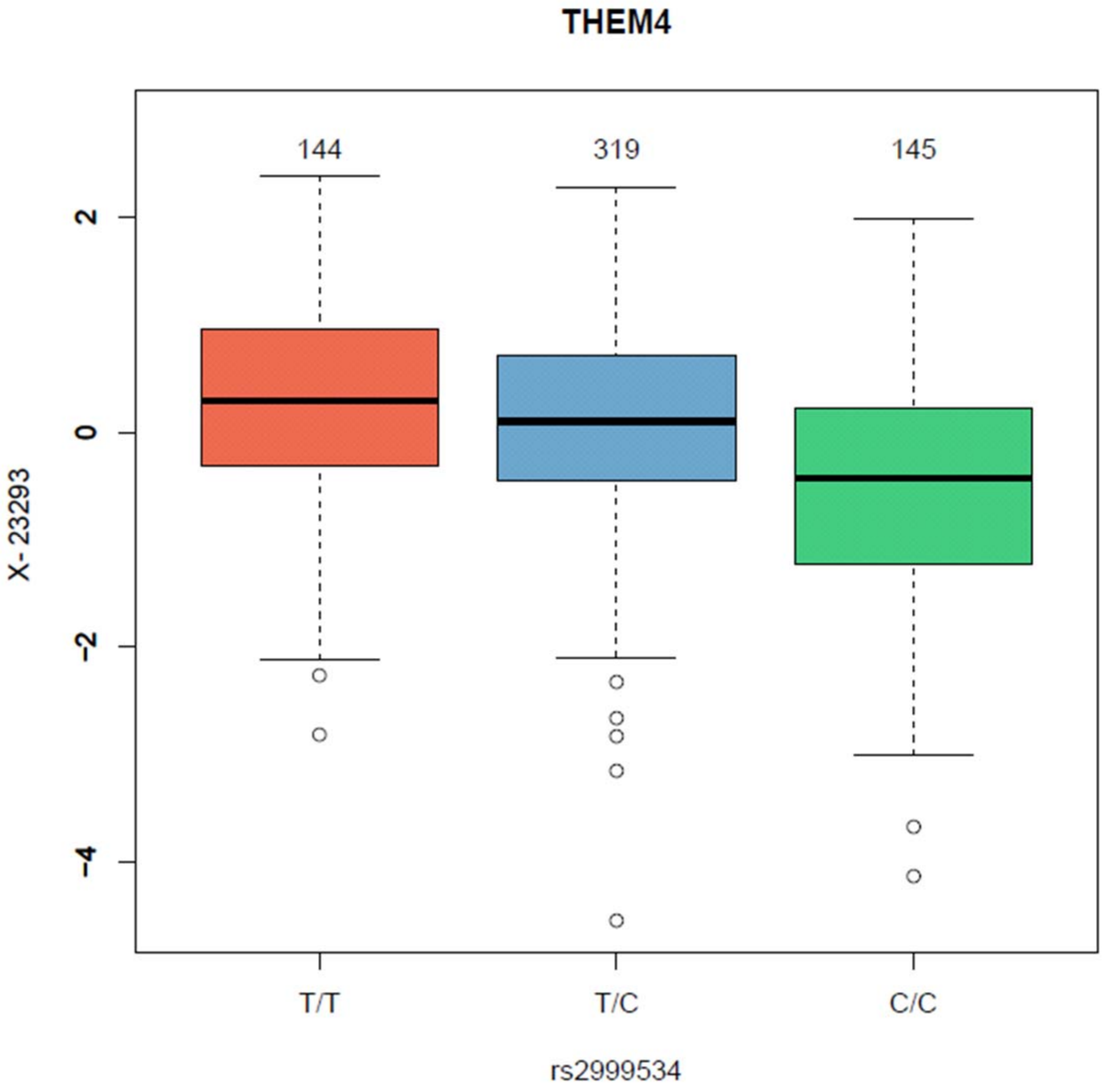
THEM4
144
319
145
2
0
-2
-4
X- 23293
T/T
T/C
C/C
rs2999534

Supplementary Figure 3: Regional Association plots for 21 common variant loci showing the significance of associations with other SNPs in the region around the sentinel SNP along with their LD to the sentinel SNP and the functional annotation. (Plots were obtained from an in house developed software adopted from SNIPA to reflect the LD between SNPs based on the Exome data used in this study.)

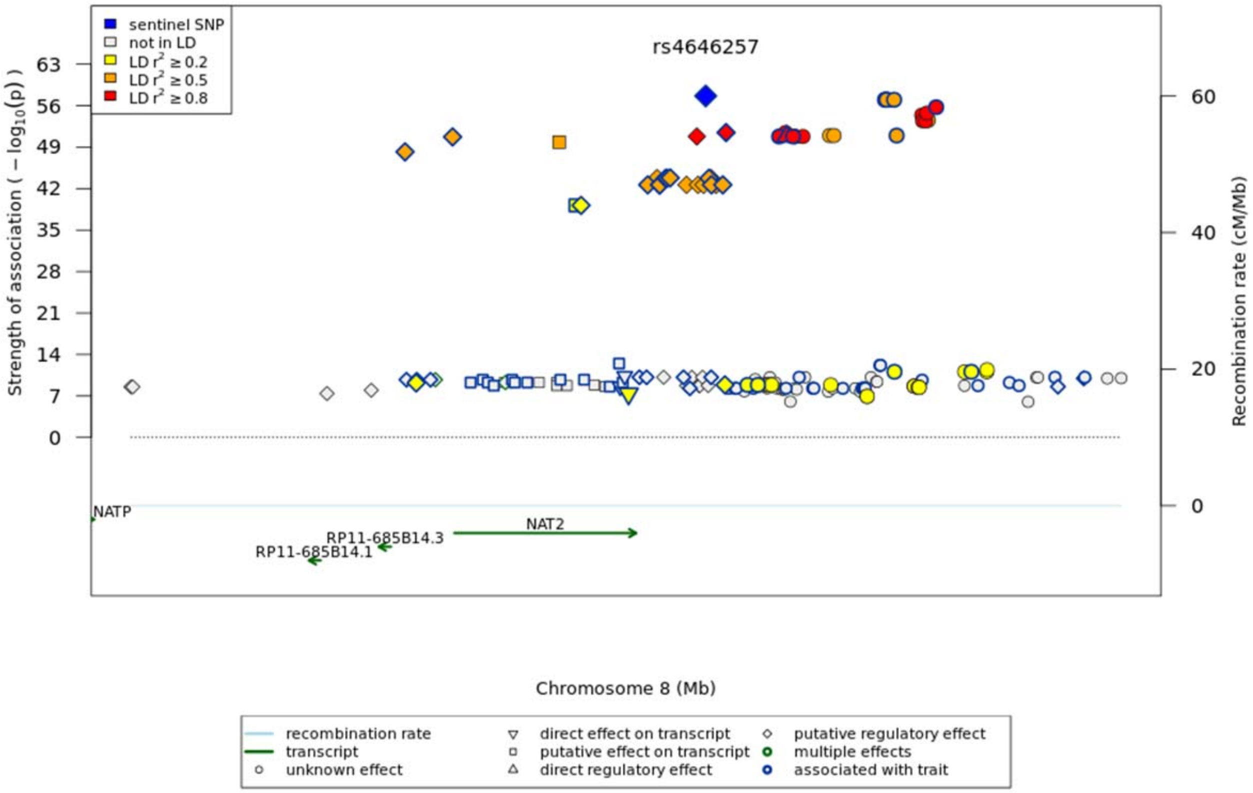

NAT2

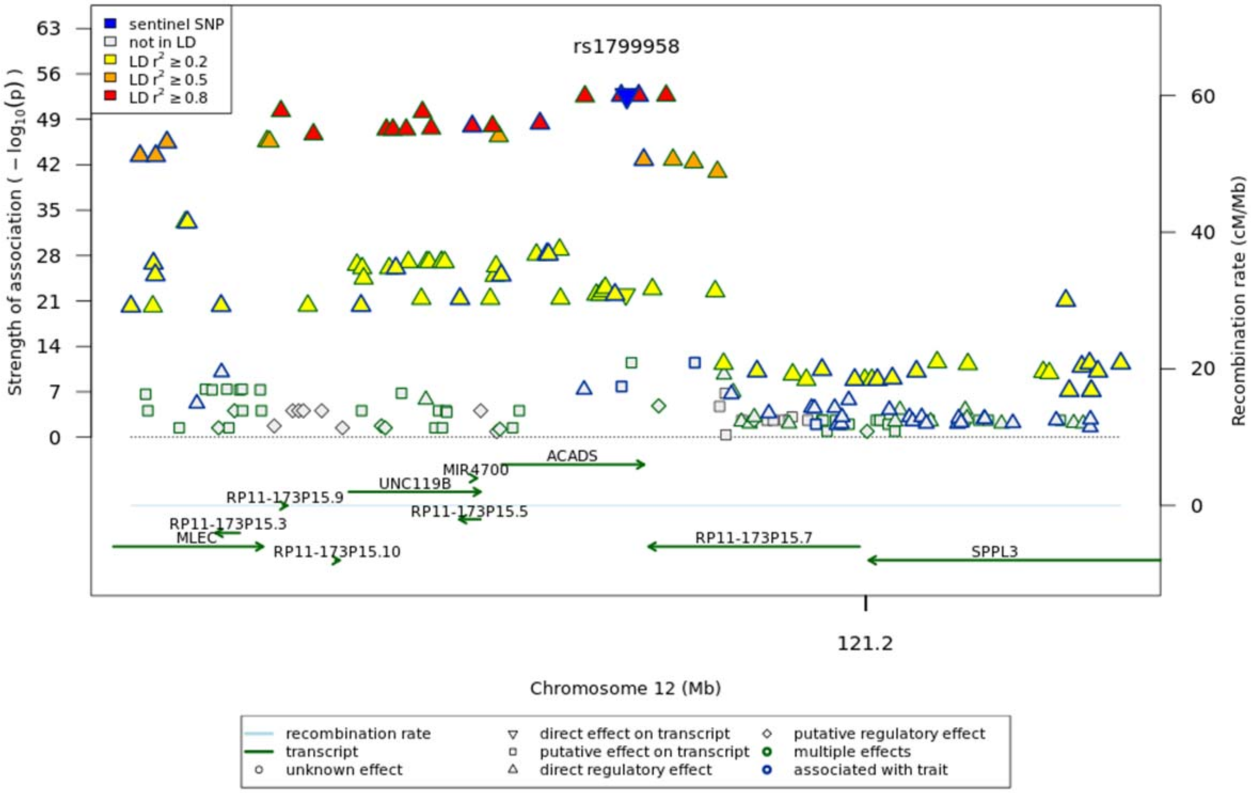

rs1799958
sentinel SNP
not in LD
LD $r^2 \geq 0.2$
LD $r^2 \geq 0.5$
LD $r^2 \geq 0.8$
Strength of association ($-\log_{10}(p)$)
Recombination rate (cM/Mb)
ACADS
MIR4700
UNC119B
RP11-173P15.9
RP11-173P15.5
RP11-173P15.3
MLEC
RP11-173P15.10
RP11-173P15.7
SPPL3
121.2
Chromosome 12 (Mb)
recombination rate
transcript
unknown effect
direct effect on transcript
putative effect on transcript
direct regulatory effect
putative regulatory effect
multiple effects
associated with trait

ACADS

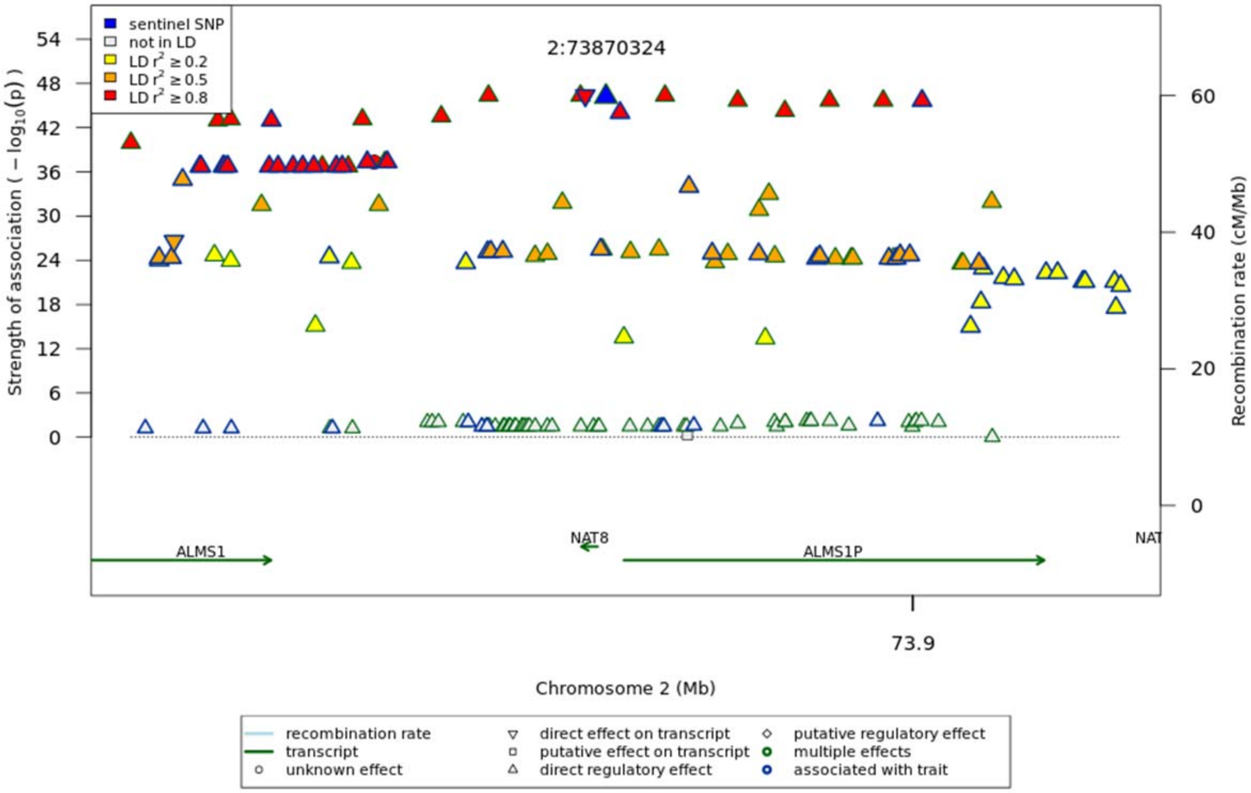

sentinel SNP
not in LD
LD r² ≥ 0.2
LD r² ≥ 0.5
LD r² ≥ 0.8
2:73870324
Strength of association (− log10(p))
Recombination rate (cM/Mb)
ALMS1
NAT8
ALMS1P
NAT
73.9
Chromosome 2 (Mb)
recombination rate
transcript
unknown effect
direct effect on transcript
putative effect on transcript
direct regulatory effect
putative regulatory effect
multiple effects
associated with trait

NAT8

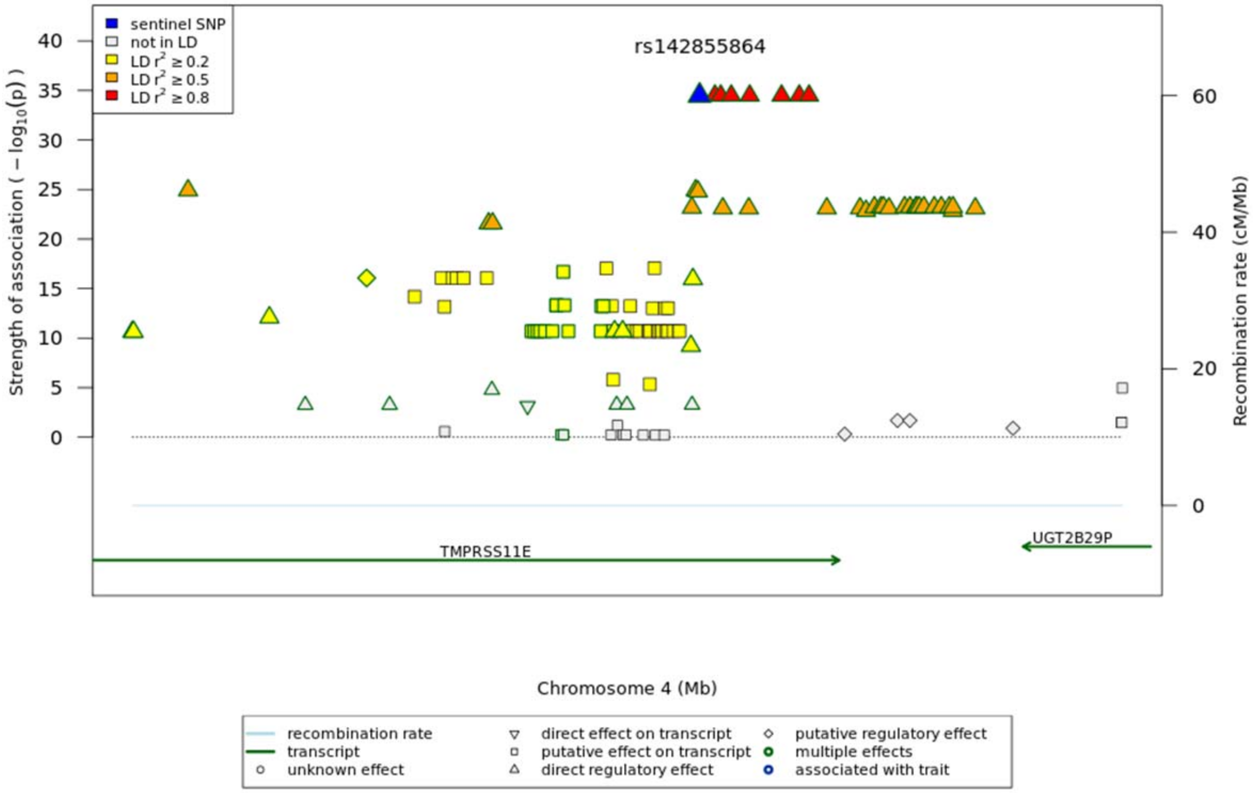

rs142855864
sentinel SNP
not in LD
LD r2 ≥ 0.2
LD r2 ≥ 0.5
LD r2 ≥ 0.8
Strength of association (− log10(p))
Recombination rate (cM/Mb)
TMPRSS11E
UGT2B29P
Chromosome 4 (Mb)
recombination rate
transcript
unknown effect
direct effect on transcript
putative effect on transcript
direct regulatory effect
putative regulatory effect
multiple effects
associated with trait

TMPRSS11E

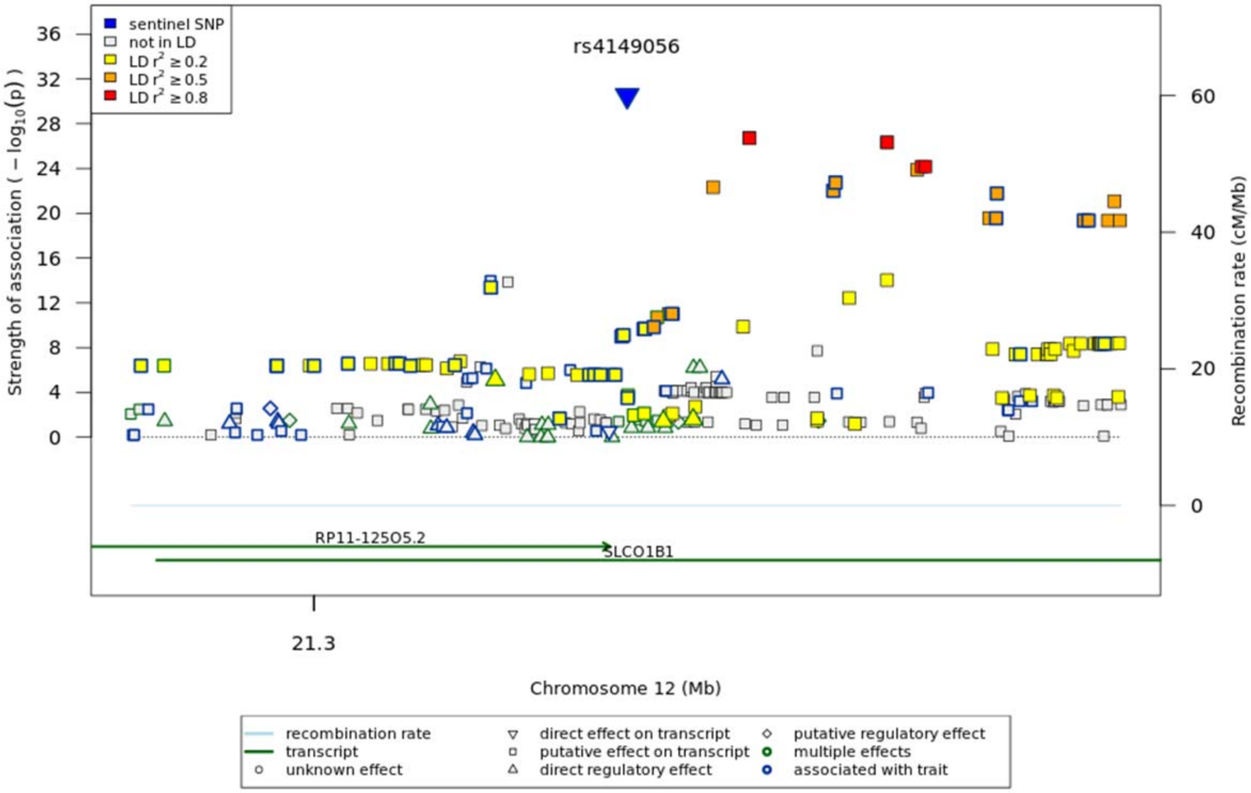

sentinel SNP
not in LD
LD r² ≥ 0.2
LD r² ≥ 0.5
LD r² ≥ 0.8
rs4149056
Strength of association (− log10(p))
Recombination rate (cM/Mb)
RP11-125O5.2
SLCO1B1
21.3
Chromosome 12 (Mb)
recombination rate
transcript
unknown effect
direct effect on transcript
putative effect on transcript
direct regulatory effect
putative regulatory effect
multiple effects
associated with trait

SLCO1B1

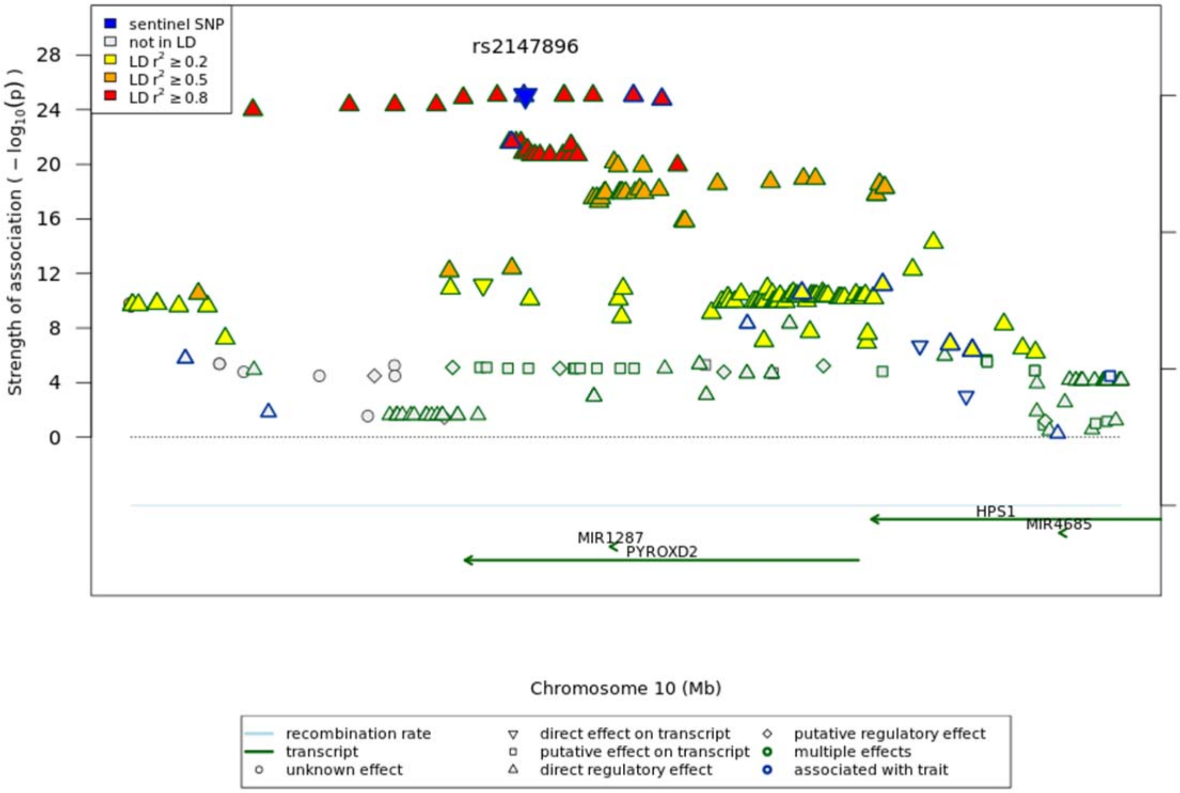
sentinel SNP
not in LD
LD r² ≥ 0.2
LD r² ≥ 0.5
LD r² ≥ 0.8
rs2147896
Strength of association (− log10(p))
Recombination rate (cM/Mb)
HPS1
MIR4685
MIR1287
PYROXD2
Chromosome 10 (Mb)
recombination rate
transcript
unknown effect
direct effect on transcript
putative effect on transcript
direct regulatory effect
putative regulatory effect
multiple effects
associated with trait

PYROXD2

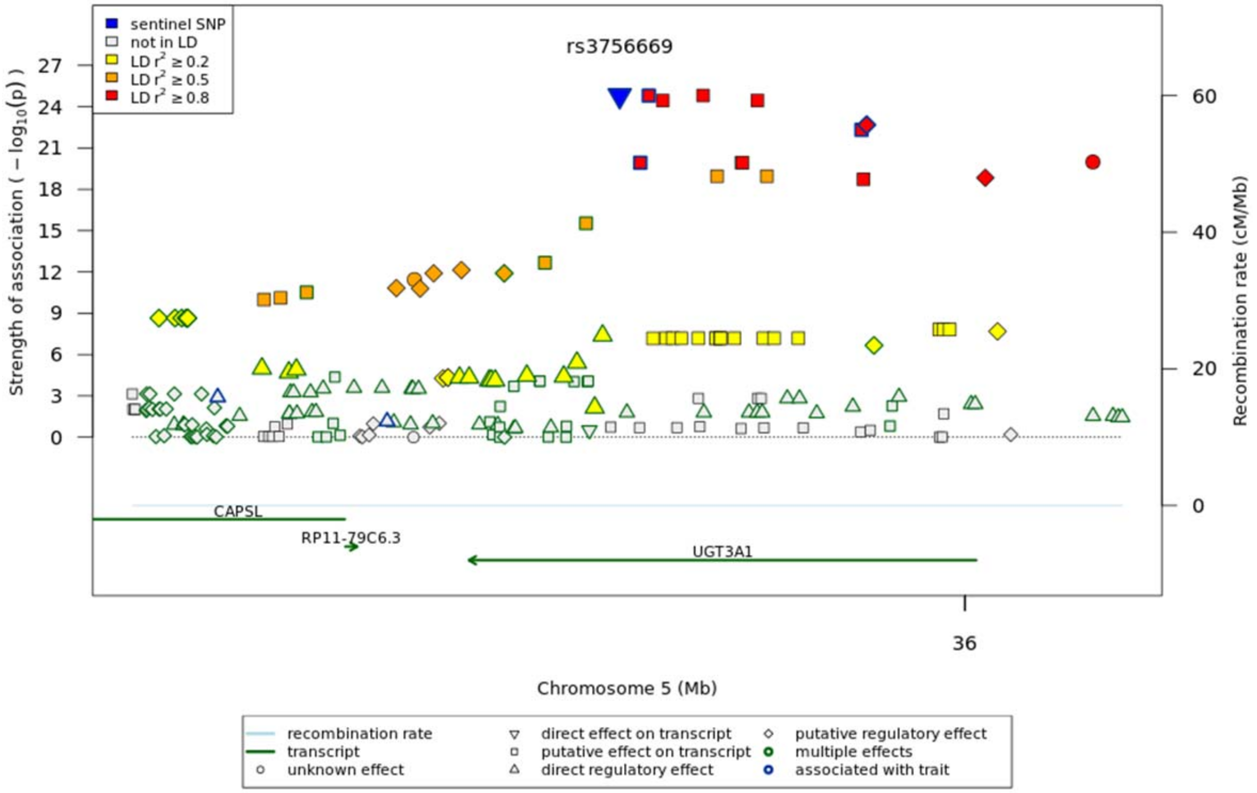

sentinel SNP
not in LD
LD $r^2 \geq 0.2$
LD $r^2 \geq 0.5$
LD $r^2 \geq 0.8$
rs3756669
Strength of association ($-\log_{10}(p)$)
Recombination rate (cM/Mb)
CAPSL
RP11-79C6.3
UGT3A1
36
Chromosome 5 (Mb)
recombination rate
transcript
unknown effect
direct effect on transcript
putative effect on transcript
direct regulatory effect
putative regulatory effect
multiple effects
associated with trait

UGT3A1

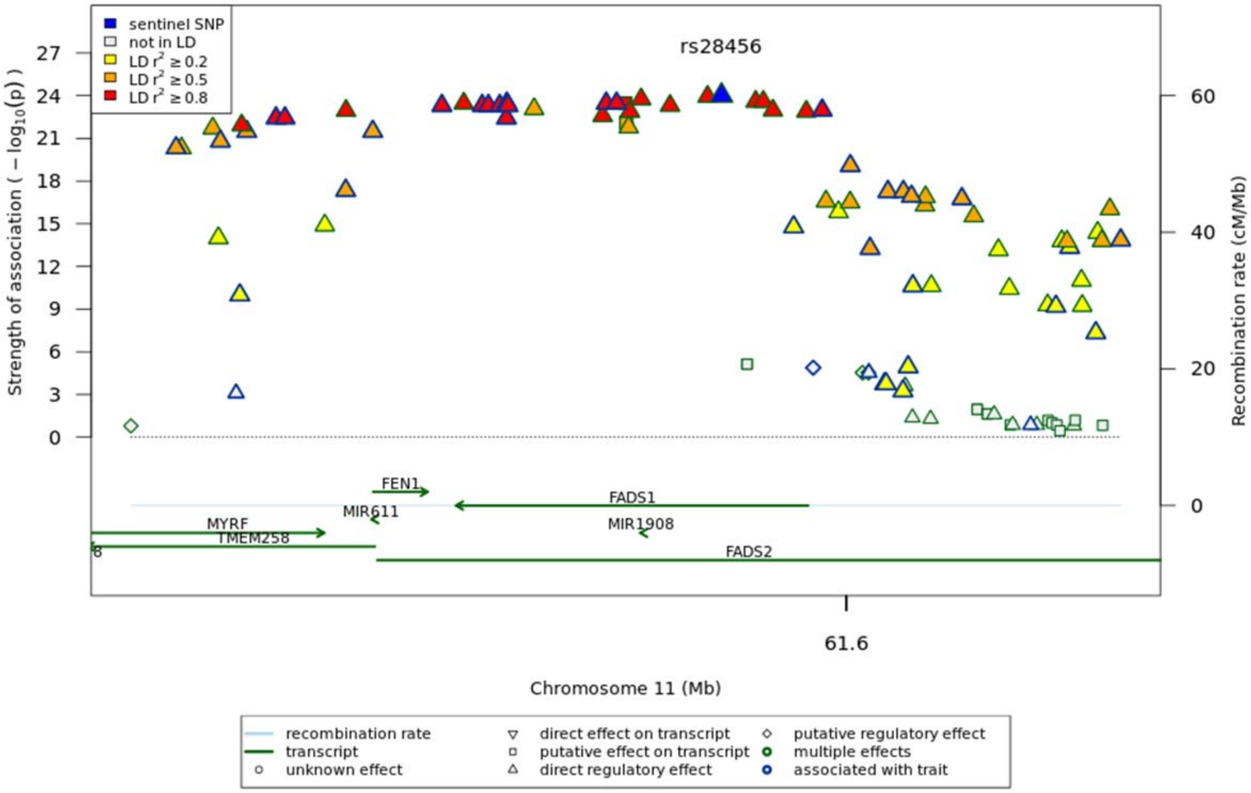

sentinel SNP
not in LD
LD r² ≥ 0.2
LD r² ≥ 0.5
LD r² ≥ 0.8
rs28456
Strength of association (− log10(p))
Recombination rate (cM/Mb)
FEN1
FADS1
MIR611
MYRF
MIR1908
TMEM258
FADS2
61.6
Chromosome 11 (Mb)
recombination rate
transcript
unknown effect
direct effect on transcript
putative effect on transcript
direct regulatory effect
putative regulatory effect
multiple effects
associated with trait

FADS

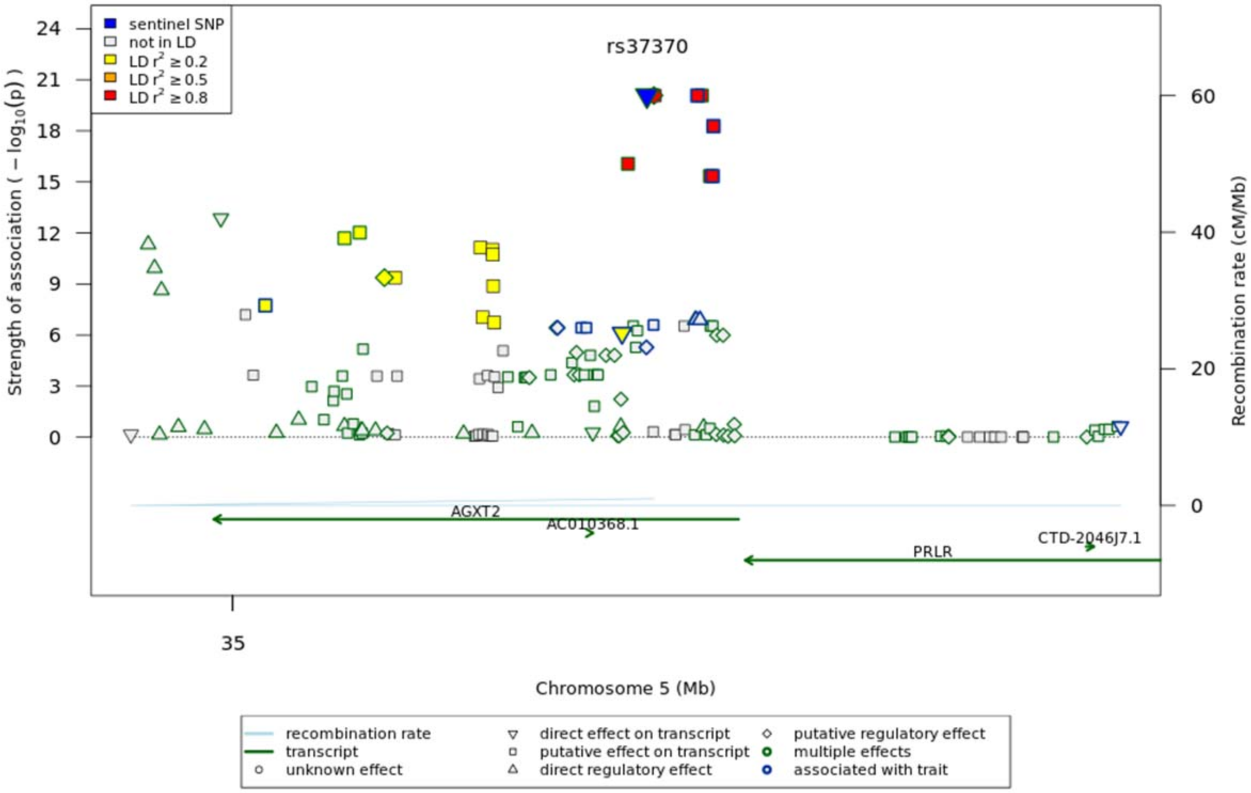

sentinel SNP
not in LD
LD $r^2 \geq 0.2$
LD $r^2 \geq 0.5$
LD $r^2 \geq 0.8$
rs37370
Strength of association ($-\log_{10}(p)$)
Recombination rate (cM/Mb)
AGXT2
AC010368.1
PRLR
CTD-2046J7.1
35
Chromosome 5 (Mb)
recombination rate
transcript
unknown effect
direct effect on transcript
putative effect on transcript
direct regulatory effect
putative regulatory effect
multiple effects
associated with trait

AGXT2

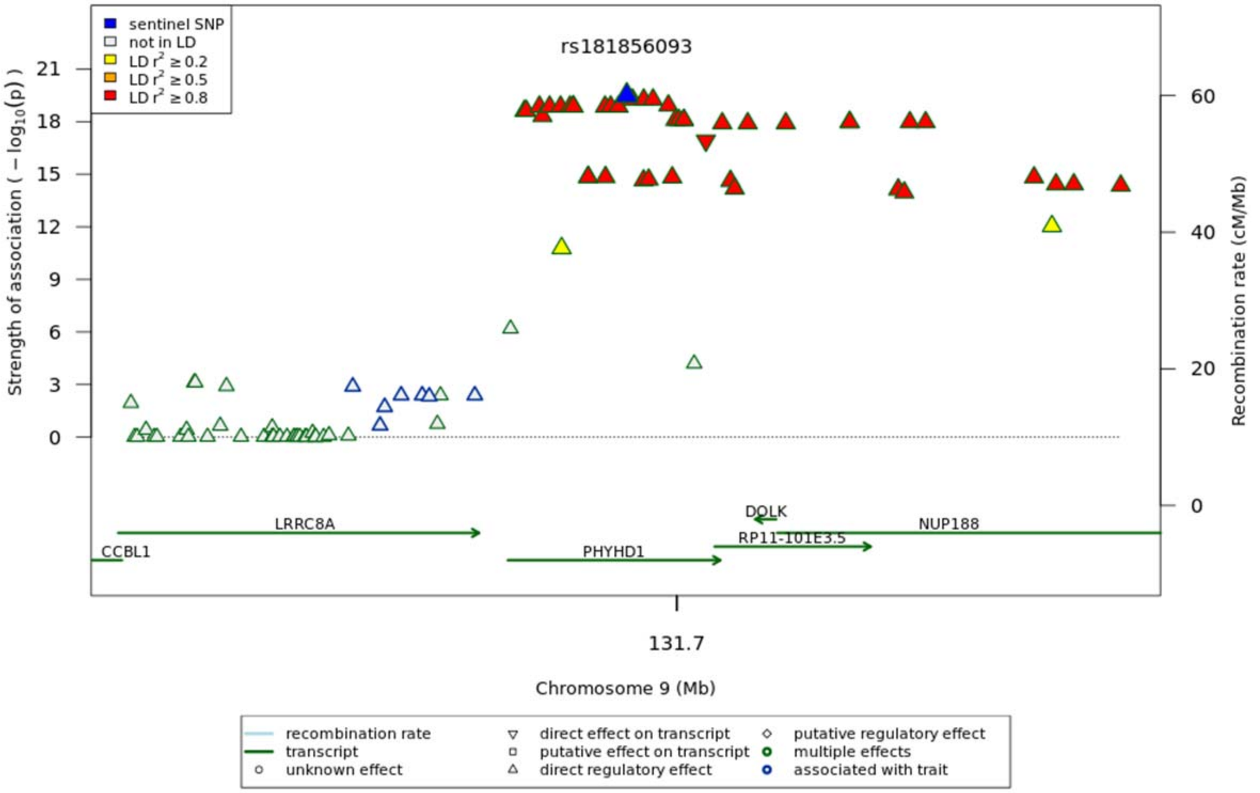

PHYHD1/NUP188

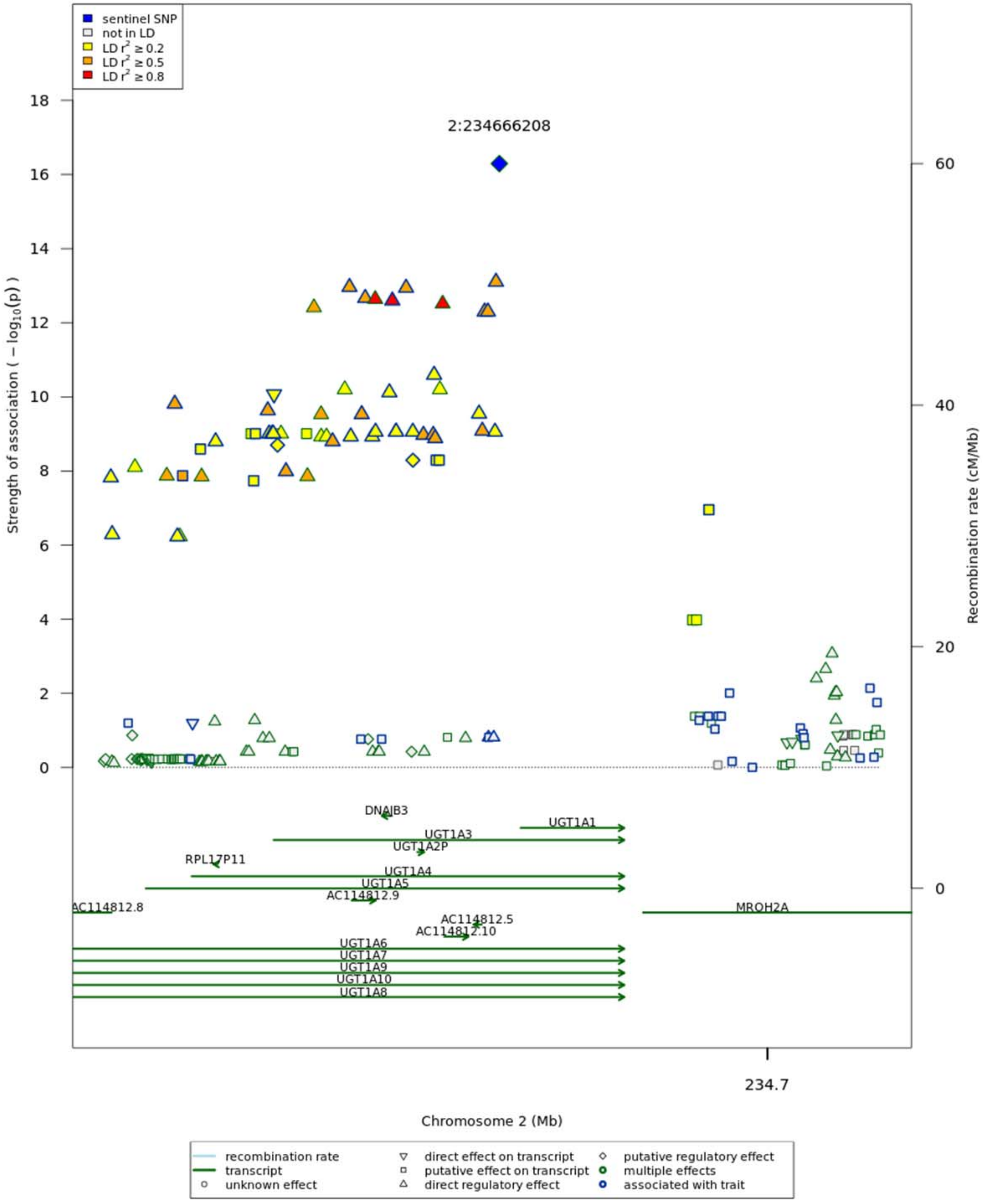
sentinel SNP
not in LD
LD $r^2 \geq 0.2$
LD $r^2 \geq 0.5$
LD $r^2 \geq 0.8$
2:234666208
Strength of association ($-\log_{10}(p)$)
Recombination rate (cM/Mb)
DNAJB3
UGT1A1
UGT1A3
UGT1A2P
RPL17P11
UGT1A4
UGT1A5
AC114812.9
AC114812.8
MROH2A
AC114812.5
AC114812.10
UGT1A6
UGT1A7
UGT1A9
UGT1A10
UGT1A8
234.7
Chromosome 2 (Mb)
recombination rate
transcript
unknown effect
direct effect on transcript
putative effect on transcript
direct regulatory effect
putative regulatory effect
multiple effects
associated with trait

UGT1A*

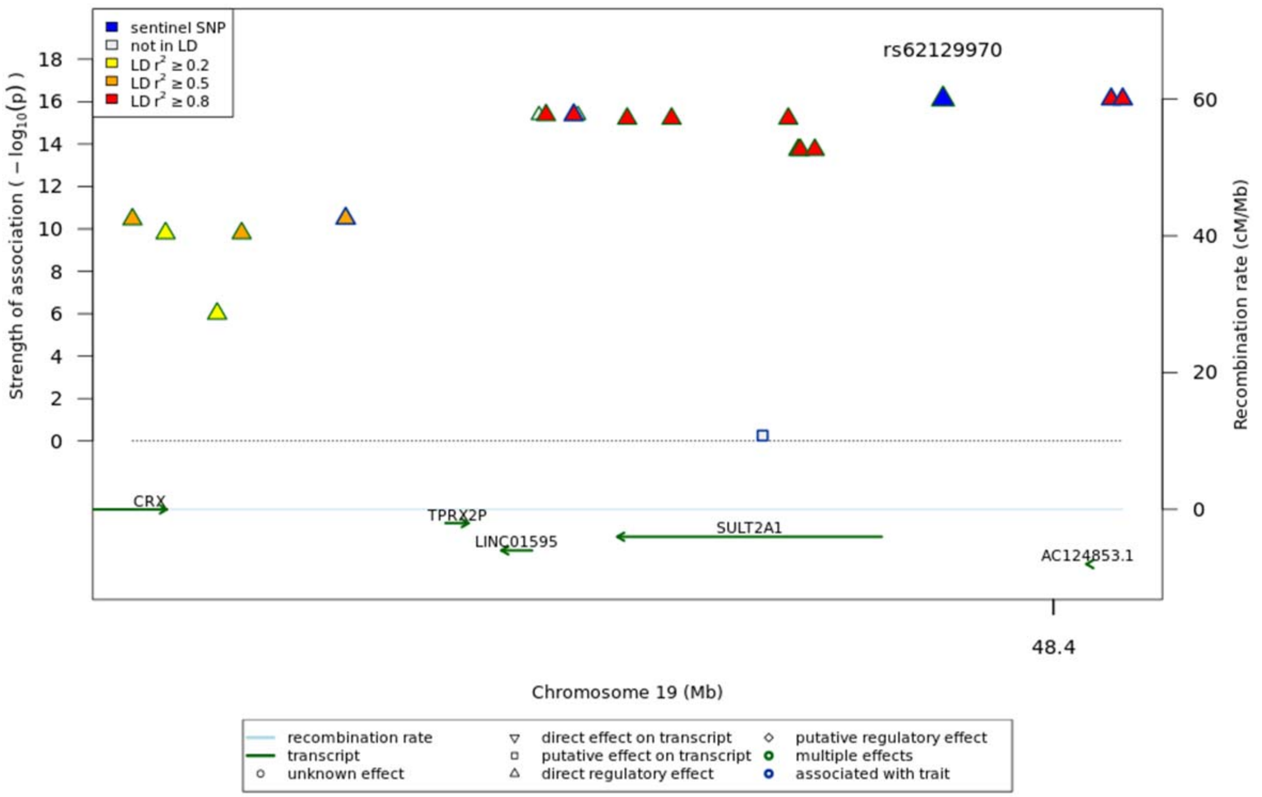

sentinel SNP
not in LD
LD $r^2 \geq 0.2$
LD $r^2 \geq 0.5$
LD $r^2 \geq 0.8$
rs62129970
Strength of association ($-\log_{10}(p)$)
Recombination rate (cM/Mb)
CRX
TPRX2P
LINC01595
SULT2A1
AC124853.1
48.4
Chromosome 19 (Mb)
recombination rate
transcript
unknown effect
direct effect on transcript
putative effect on transcript
direct regulatory effect
putative regulatory effect
multiple effects
associated with trait

SULT2A1

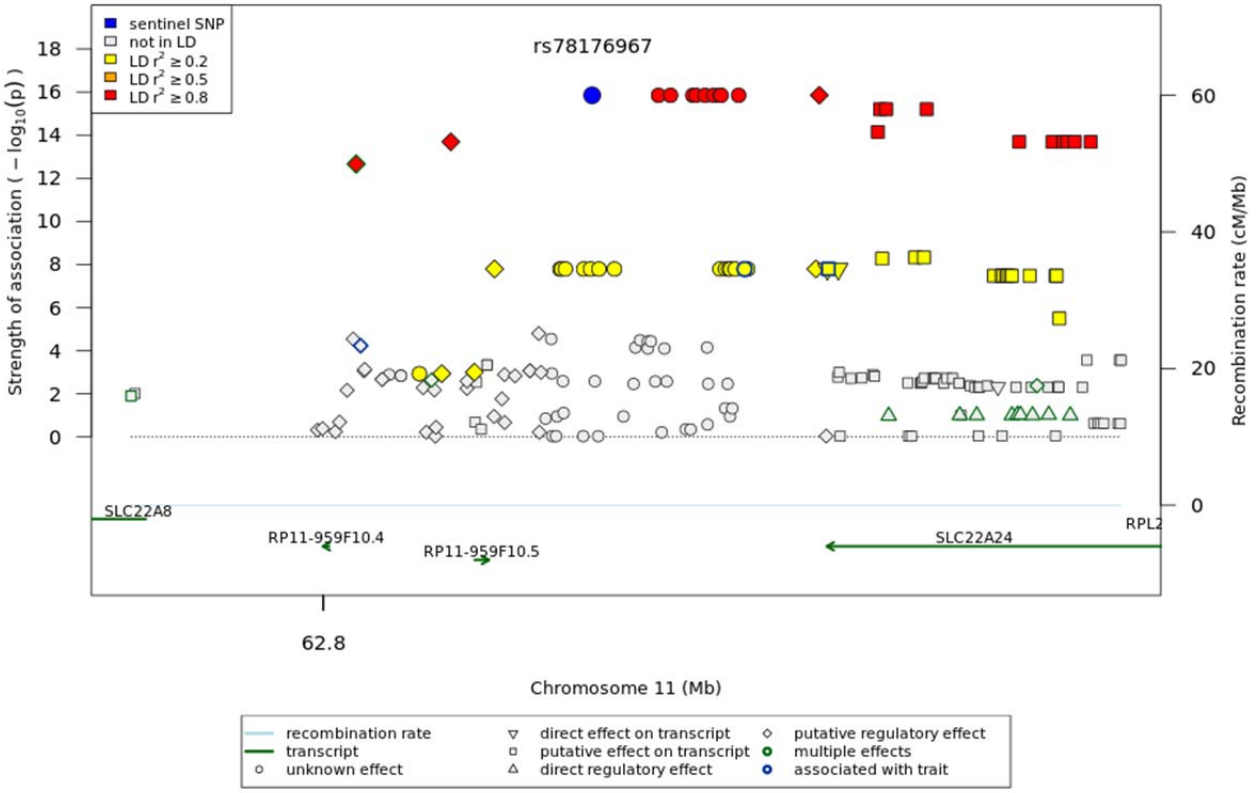

sentinel SNP
not in LD
LD r² ≥ 0.2
LD r² ≥ 0.5
LD r² ≥ 0.8
rs78176967
Strength of association (− log10(p))
Recombination rate (cM/Mb)
SLC22A8
RP11-959F10.4
RP11-959F10.5
SLC22A24
RPL2
62.8
Chromosome 11 (Mb)
recombination rate
transcript
unknown effect
direct effect on transcript
putative effect on transcript
direct regulatory effect
putative regulatory effect
multiple effects
associated with trait

SLC22A24

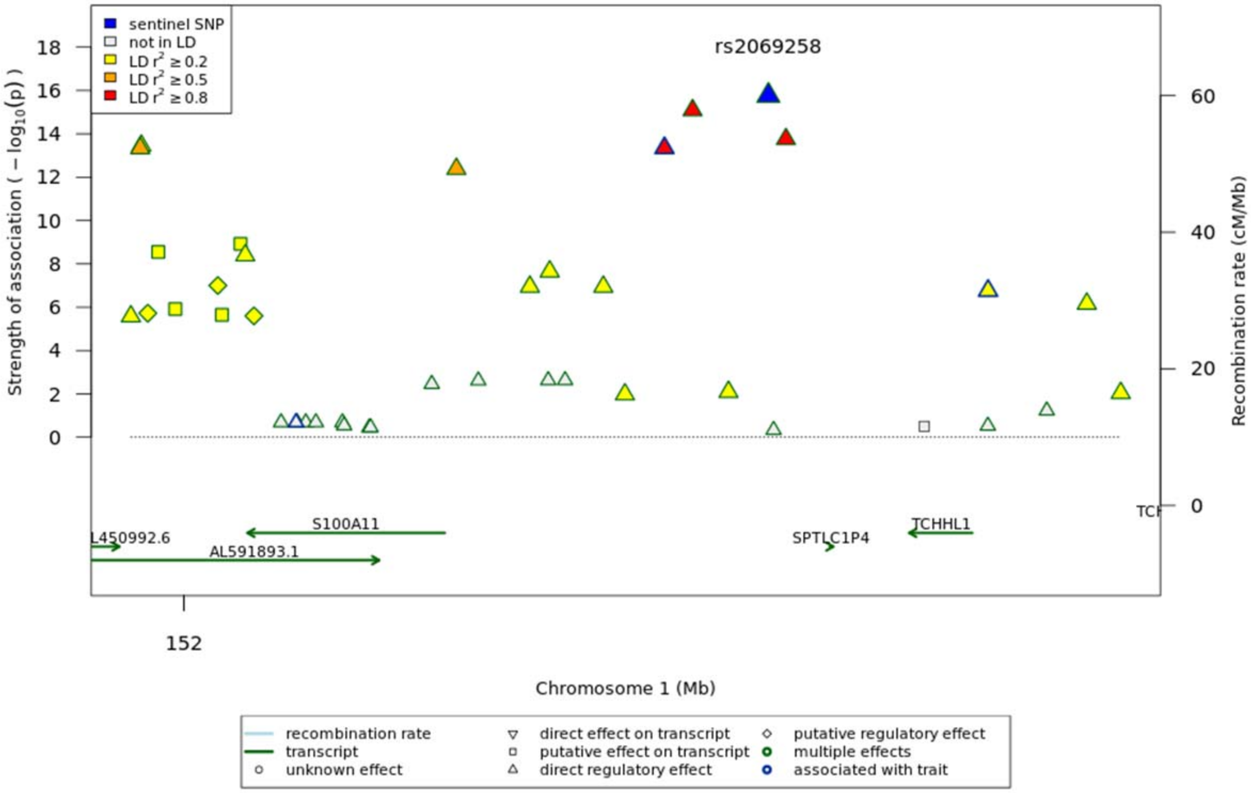

SPTLC1P4/AL591893.1

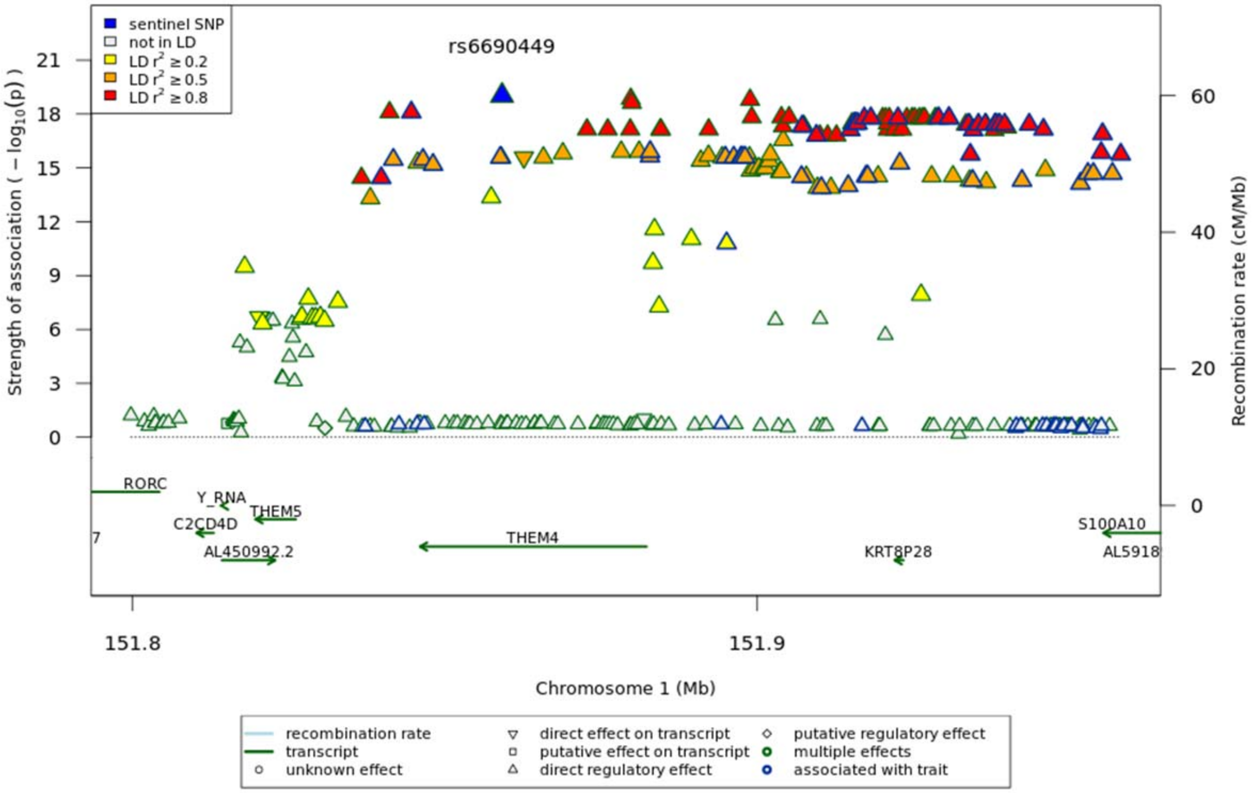

rs6690449
sentinel SNP
not in LD
LD r² ≥ 0.2
LD r² ≥ 0.5
LD r² ≥ 0.8
Strength of association (− log10(p))
Recombination rate (cM/Mb)
RORC
Y_RNA
THEM5
C2CD4D
AL450992.2
THEM4
KRT8P28
S100A10
AL5918
151.8
151.9
Chromosome 1 (Mb)
recombination rate
transcript
unknown effect
direct effect on transcript
putative effect on transcript
direct regulatory effect
putative regulatory effect
multiple effects
associated with trait

THEM4

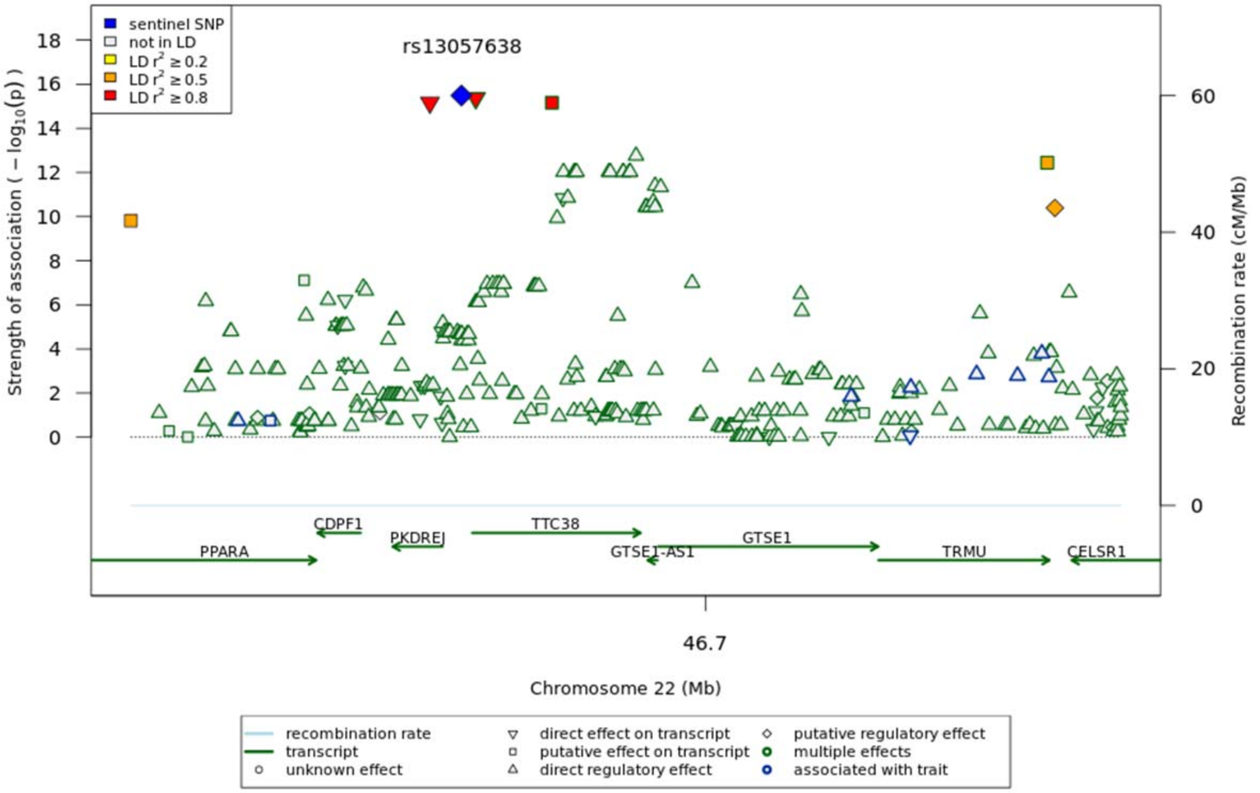

TTC38/PKDREJ

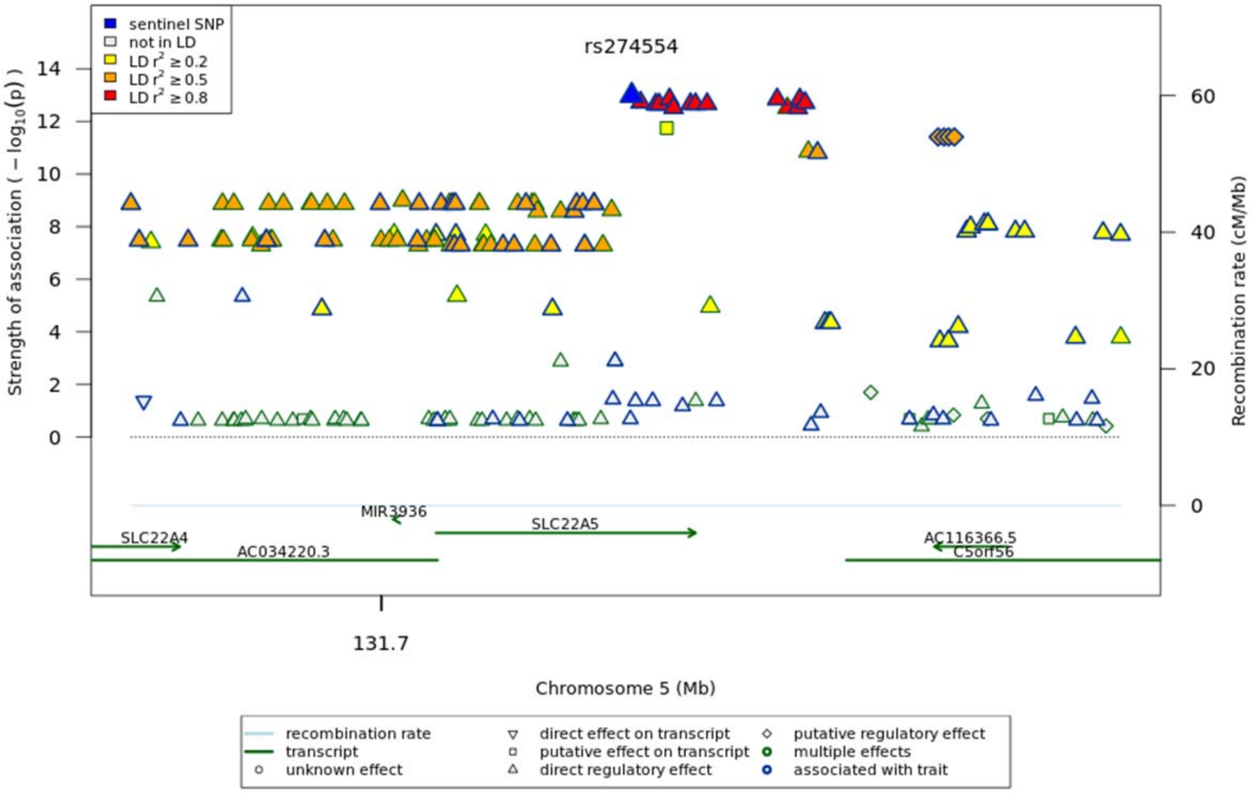

rs274554
sentinel SNP
not in LD
LD r² ≥ 0.2
LD r² ≥ 0.5
LD r² ≥ 0.8
Strength of association (− log10(p))
Recombination rate (cM/Mb)
MIR3936
SLC22A4
SLC22A5
AC034220.3
AC116366.5
C5orf56
131.7
Chromosome 5 (Mb)
recombination rate
transcript
unknown effect
direct effect on transcript
putative effect on transcript
direct regulatory effect
putative regulatory effect
multiple effects
associated with trait

SLC22A5

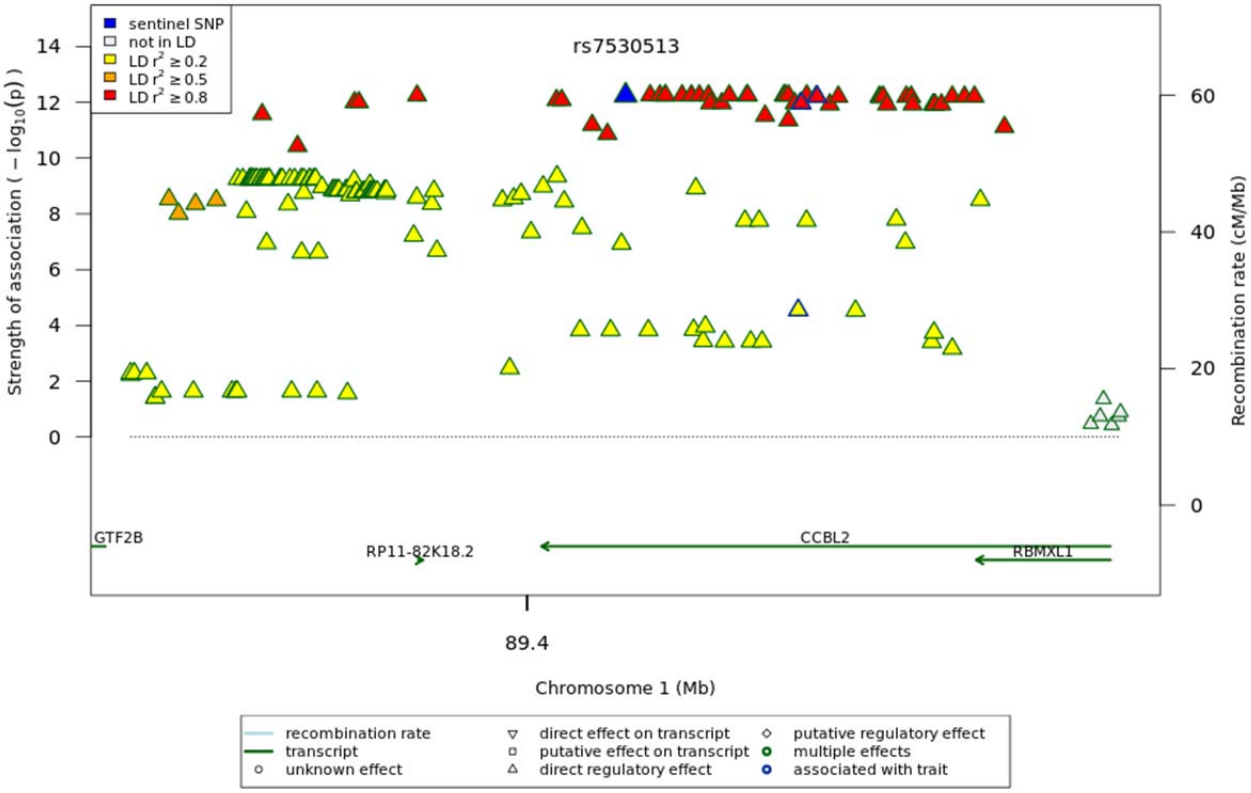
rs7530513
sentinel SNP
not in LD
LD r² ≥ 0.2
LD r² ≥ 0.5
LD r² ≥ 0.8
Strength of association (− log10(p))
Recombination rate (cM/Mb)
GTF2B
RP11-82K18.2
CCBL2
RBMXL1
89.4
Chromosome 1 (Mb)
recombination rate
transcript
unknown effect
direct effect on transcript
putative effect on transcript
direct regulatory effect
putative regulatory effect
multiple effects
associated with trait

CCBL2

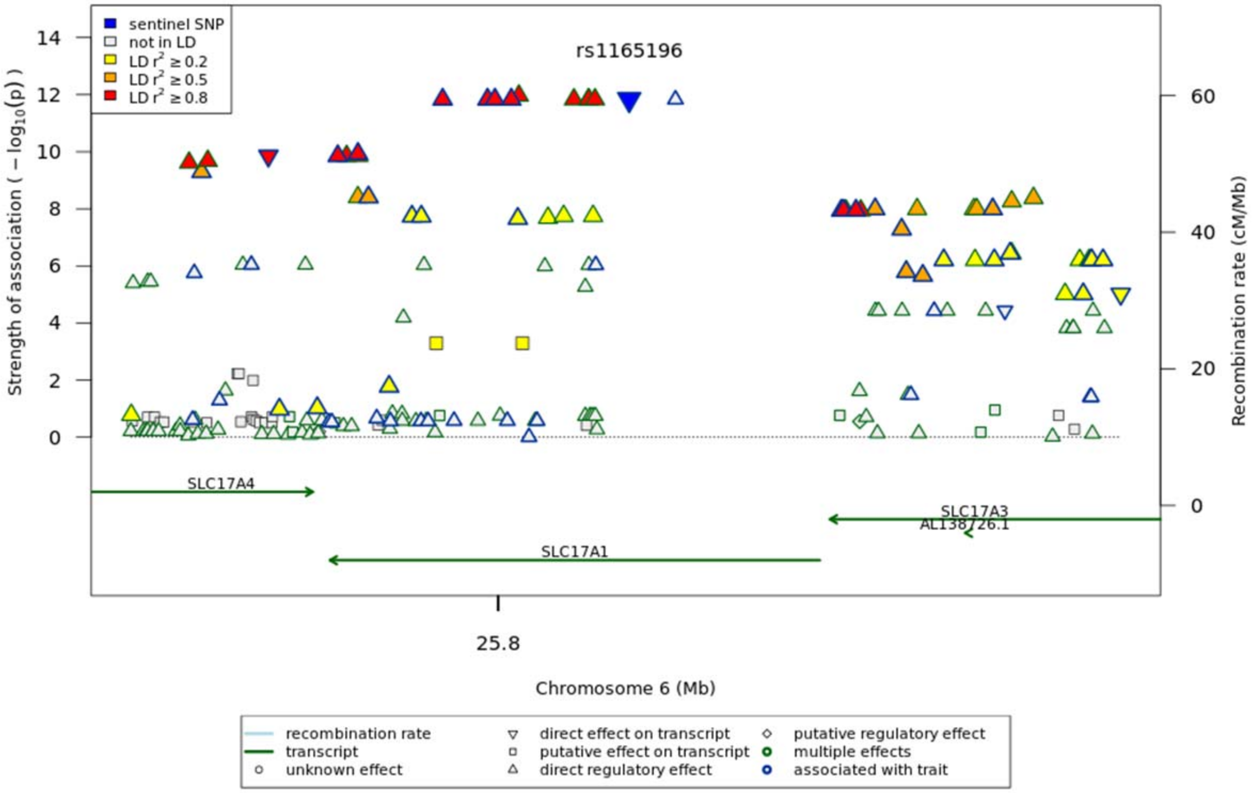
rs1165196
sentinel SNP
not in LD
LD r² ≥ 0.2
LD r² ≥ 0.5
LD r² ≥ 0.8
Strength of association (− log10(p))
Recombination rate (cM/Mb)
SLC17A4
SLC17A1
SLC17A3
AL138726.1
25.8
Chromosome 6 (Mb)
recombination rate
transcript
unknown effect
direct effect on transcript
putative effect on transcript
direct regulatory effect
putative regulatory effect
multiple effects
associated with trait

SLC17A1

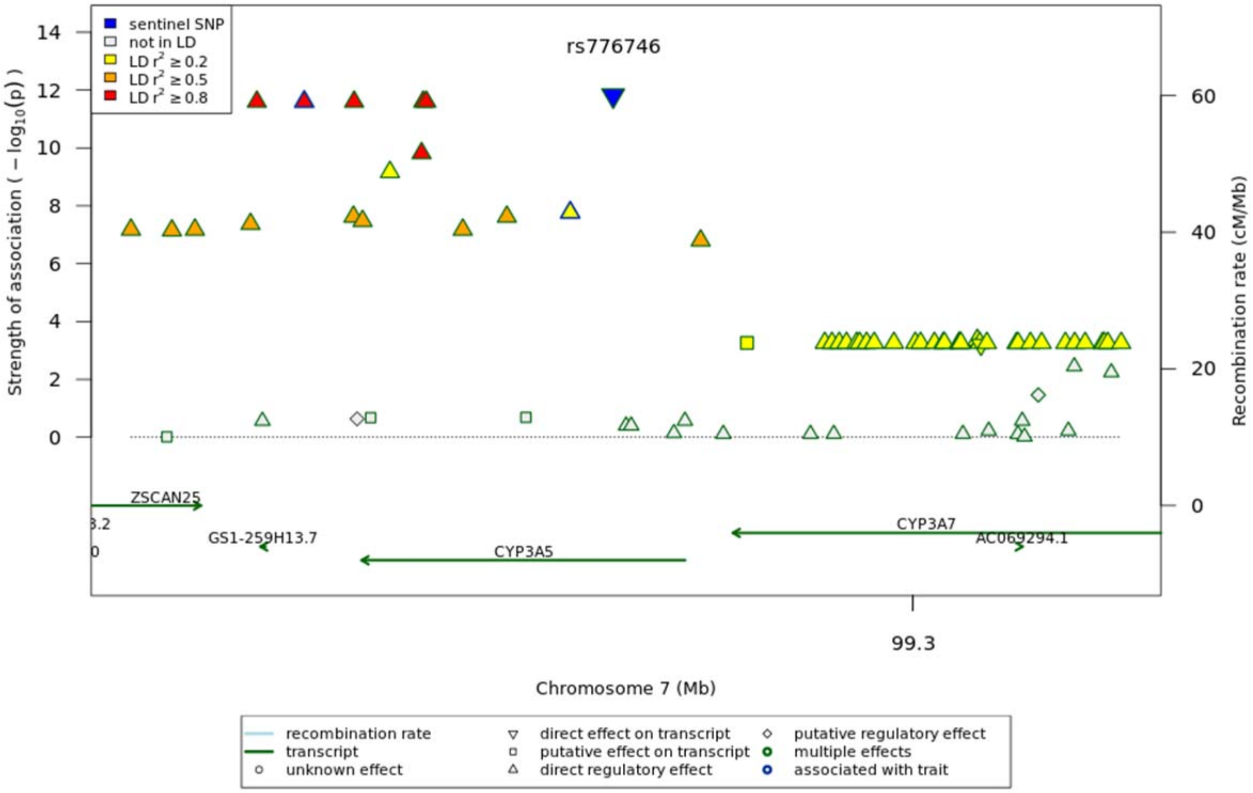

CYP3A5

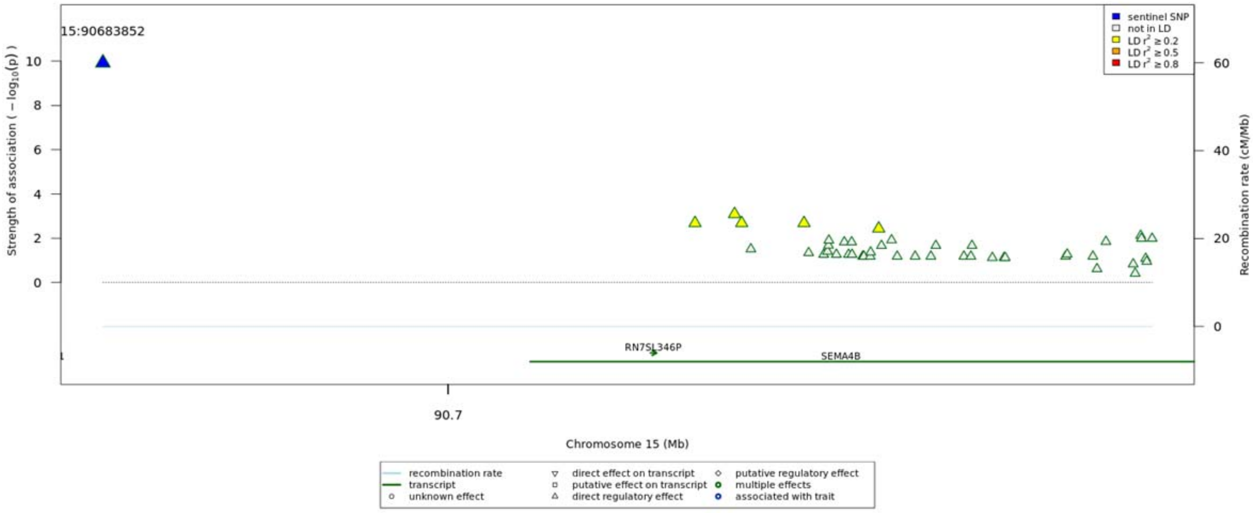

SEMA4B

Supplementary Figure 4: Steroid GGM, NAT2 locus association with GGM related metabolites, and KEGG pathway for caffeine metabolism.

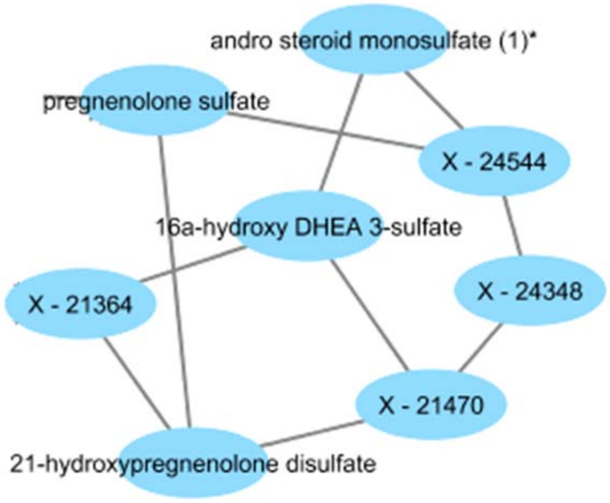

Supplementary Figure 4.a: X-24348 significantly partially correlates to unknown metabolites that correlate with steroids (Graph cut from a bigger GGM network).

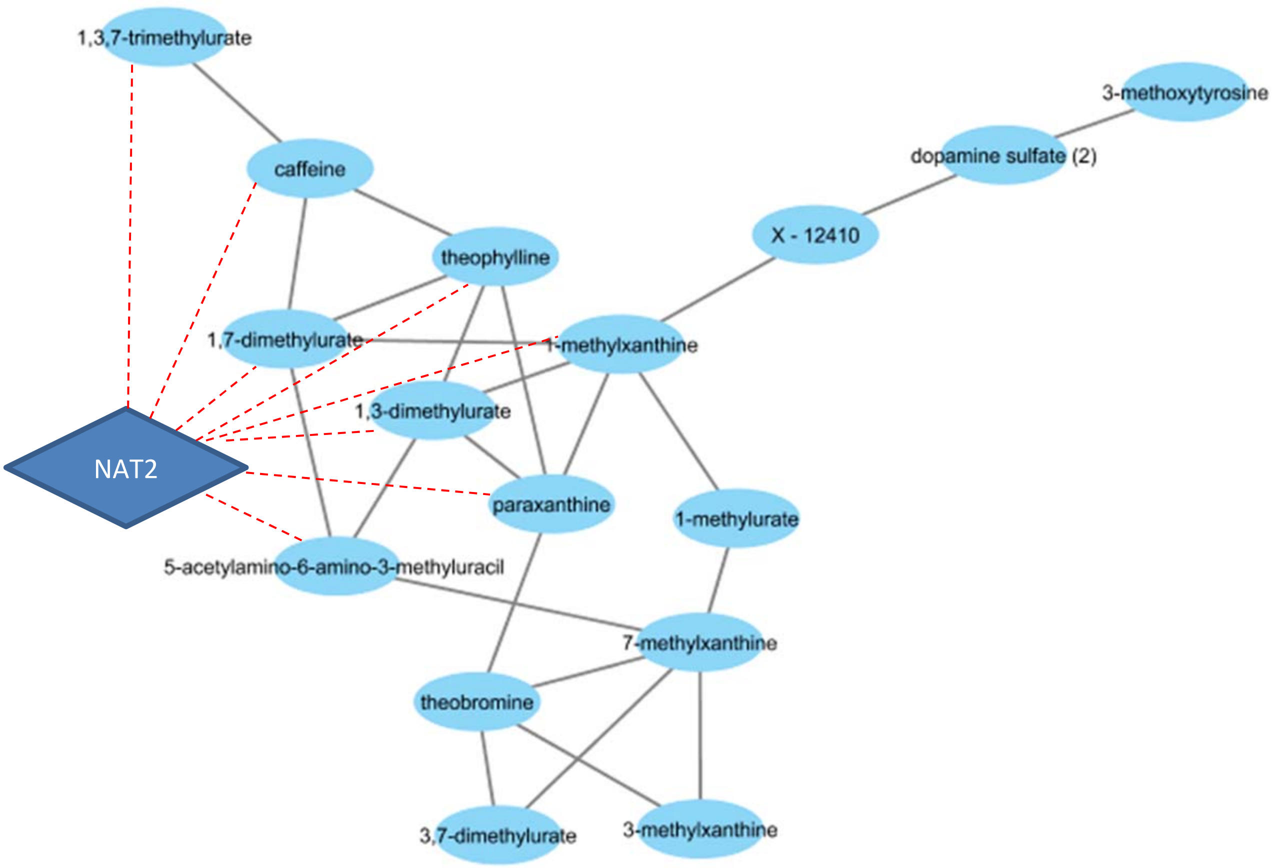

Supplementary Figure 4.b: NAT2 gene association with metabolites in the caffeine metabolism pathway (red dashed lines), which are significantly partially correlated to metabolites in the same pathway and which appear in association with the locus.

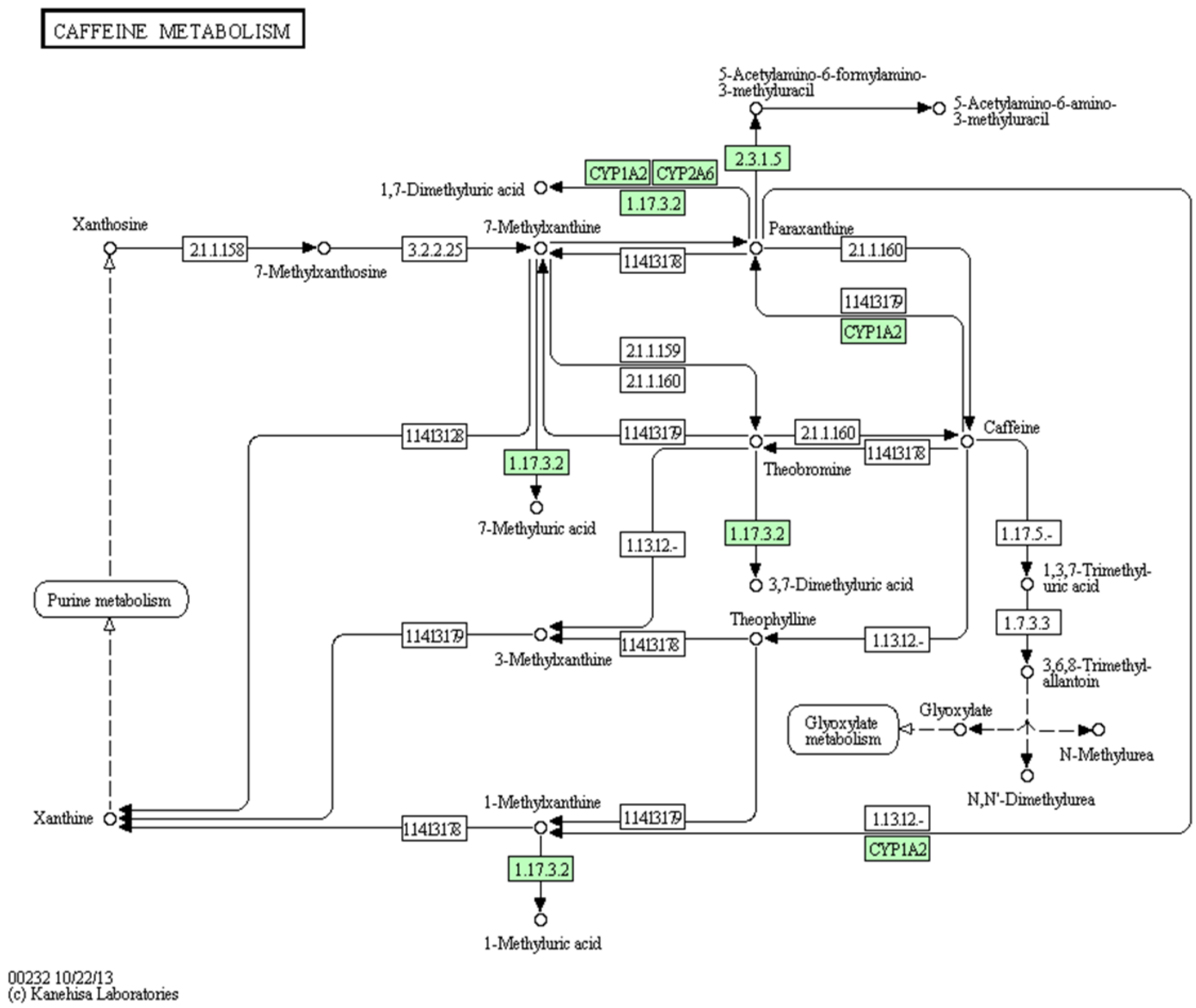

Supplementary Figure 4.c: Caffeine metabolism (KEGG) indicating the enzyme E2.3.1.5 (NAT2) is involved in producing 5-acetylamino-6-amino-3-methyluracil from paraxanthine, where the ratio between the two metabolites is associated with the locus. The pathway also shows the pathway between 1-methylxanthine and paraxanthine. In this locus, the ratio of 5-acetylamino-6-amino-3-methyluracil/1-methylxanthine shows a stronger association to the SNP in NAT2.

Supplementary Figure 5: Boxplots of rare variant loci.

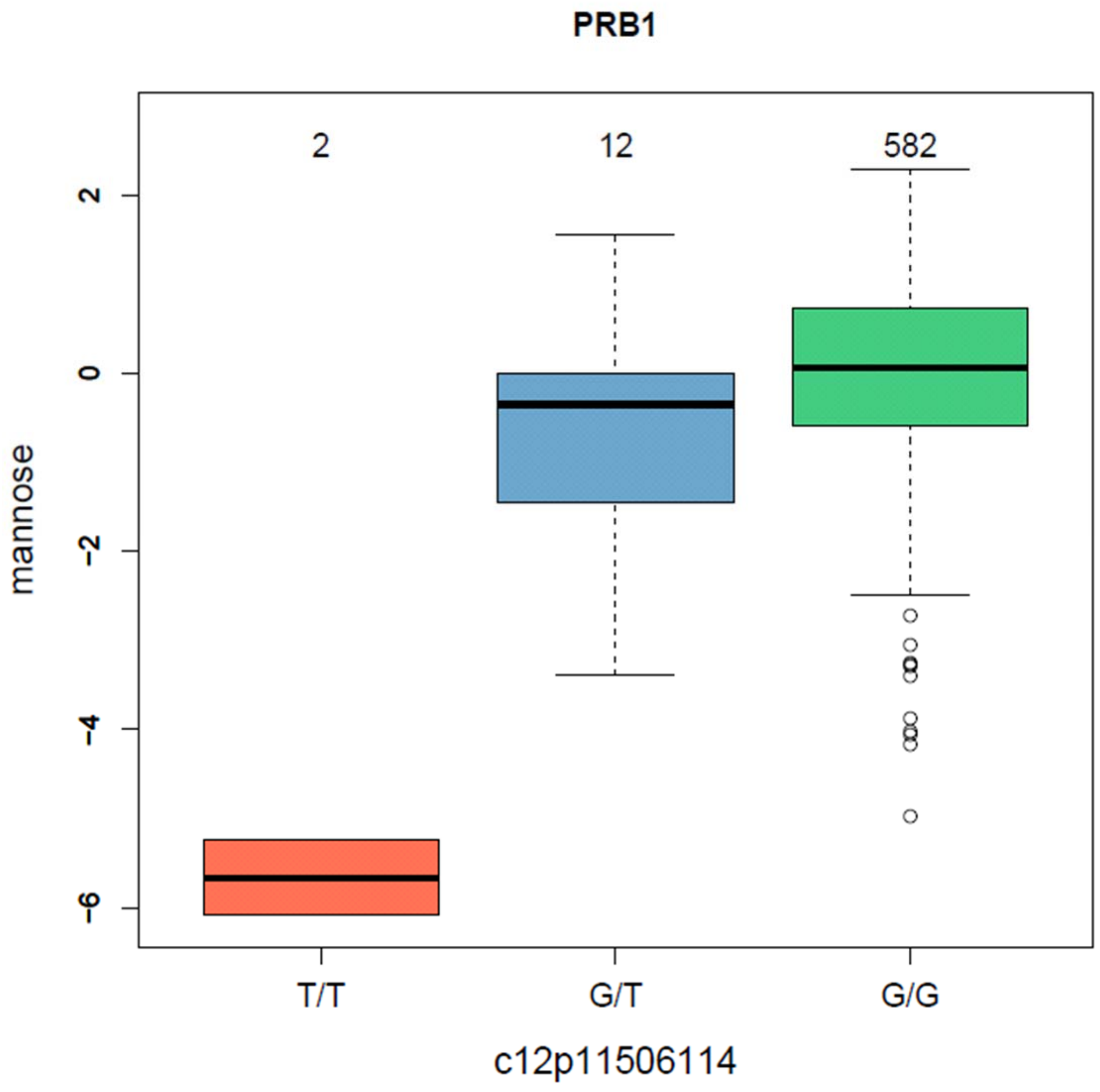

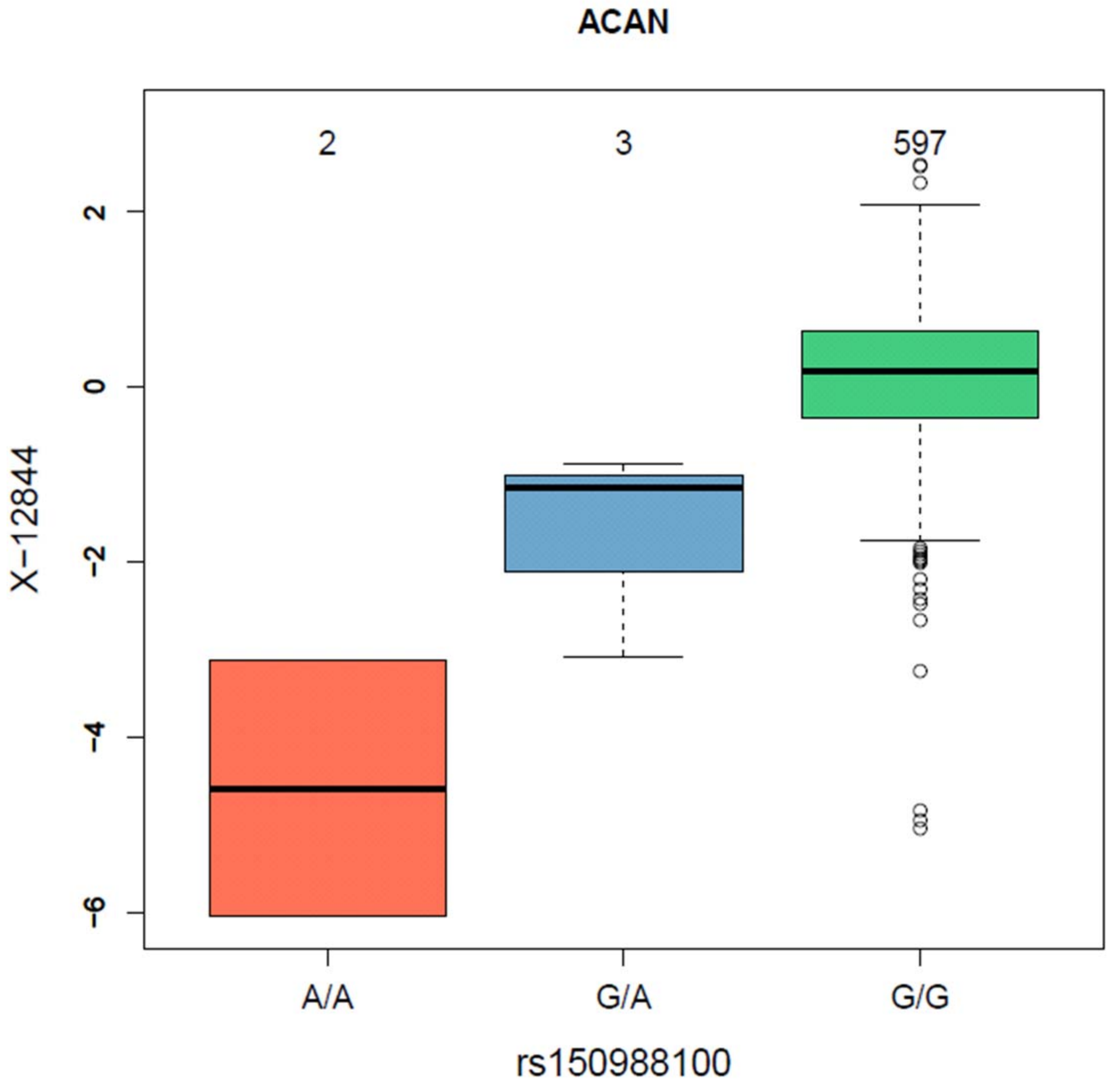
ACAN
2
3
597
2
0
-2
-4
-6
X−12844
A/A
G/A
G/G
rs150988100

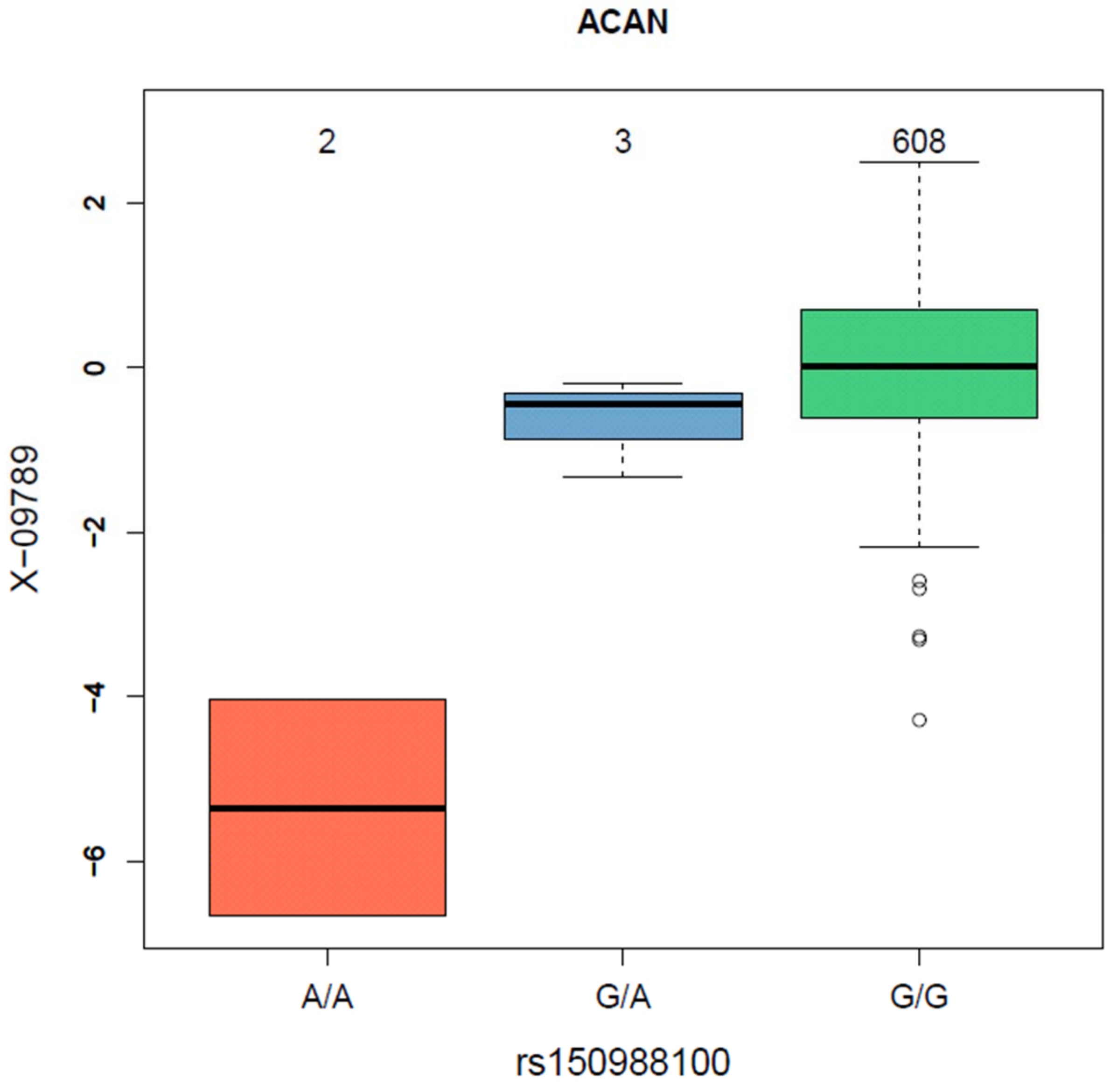

ACAN
2
3
608
2
0
-2
-4
-6
X−09789
A/A
G/A
G/G
rs150988100

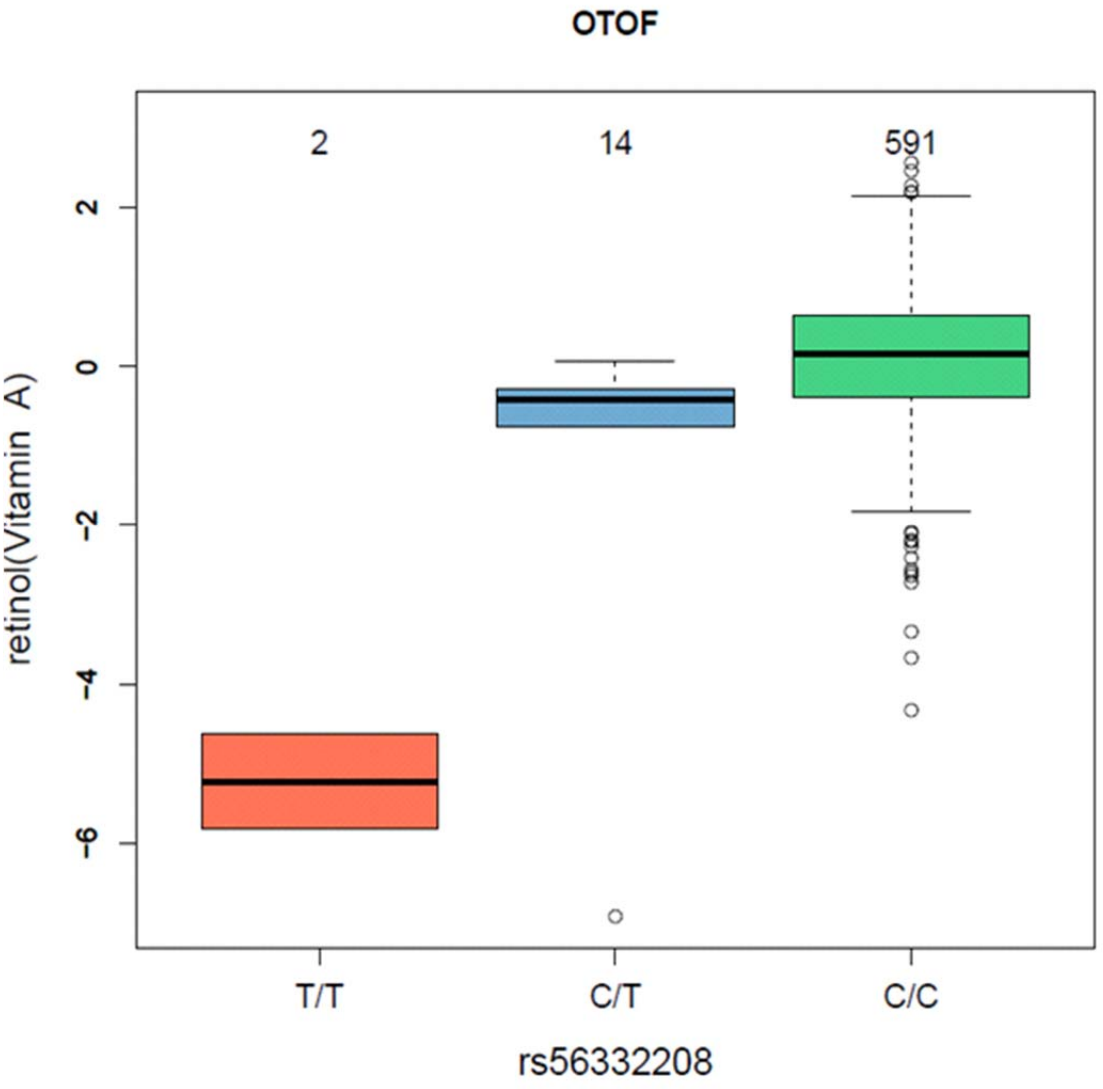
OTOF
2
14
591
2
0
-2
-4
-6
retinol(Vitamin A)
T/T
C/T
C/C
rs56332208

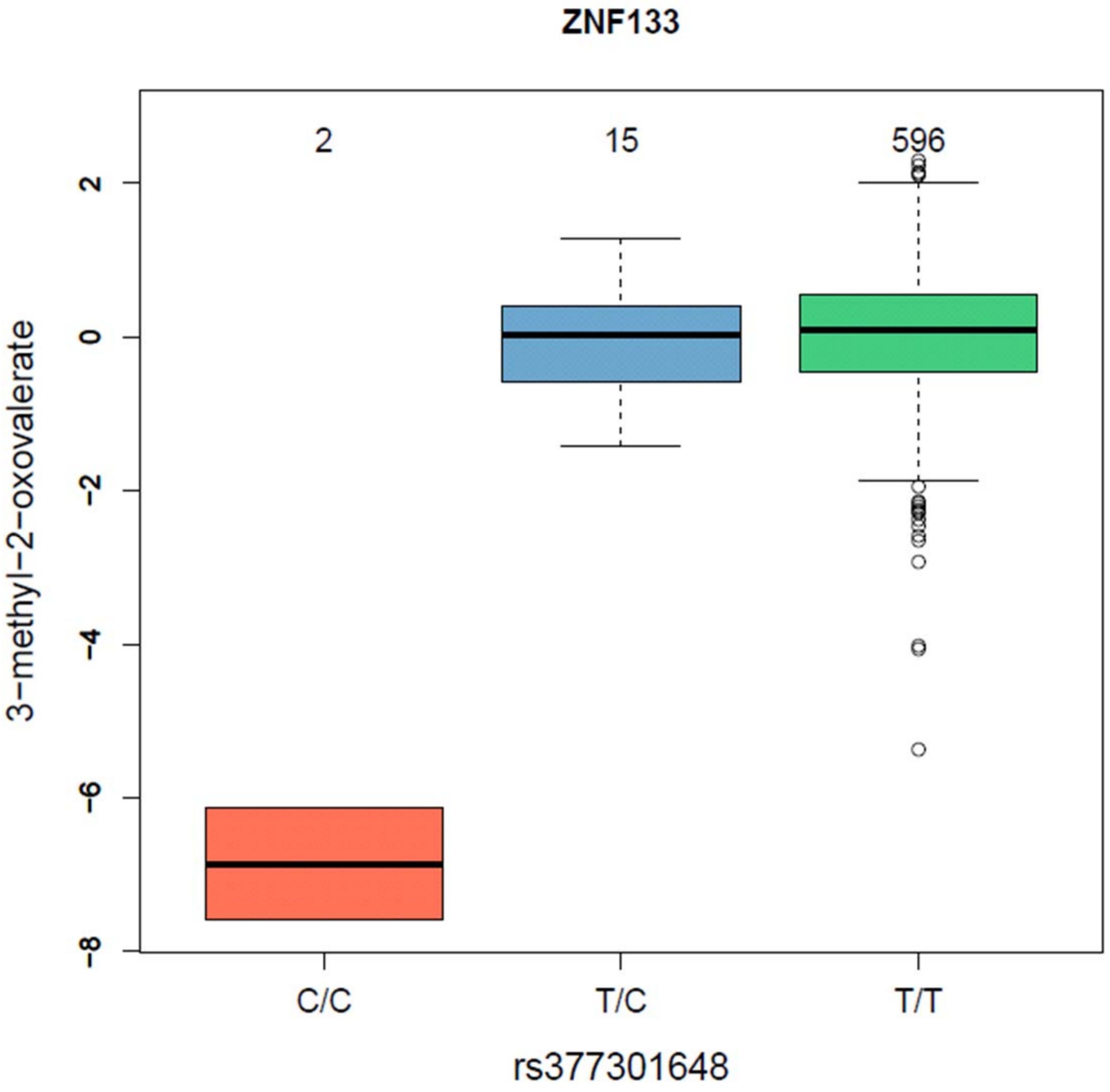
ZNF133
2
15
596
2
0
-2
-4
-6
-8
3−methyl−2−oxovalerate
C/C
T/C
T/T
rs377301648

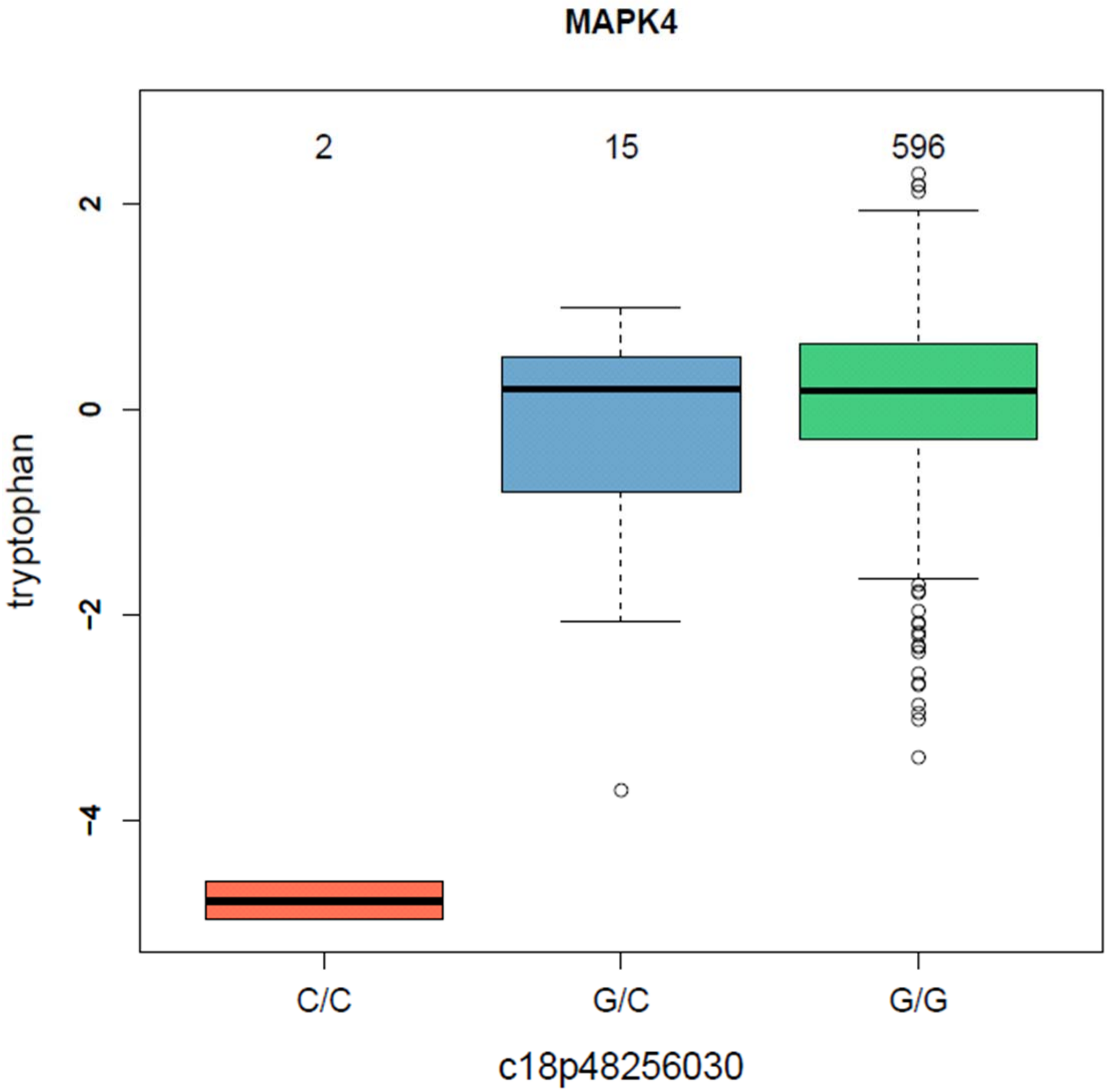
MAPK4
2
15
596
2
0
-2
-4
tryptophan
C/C
G/C
G/G
c18p48256030

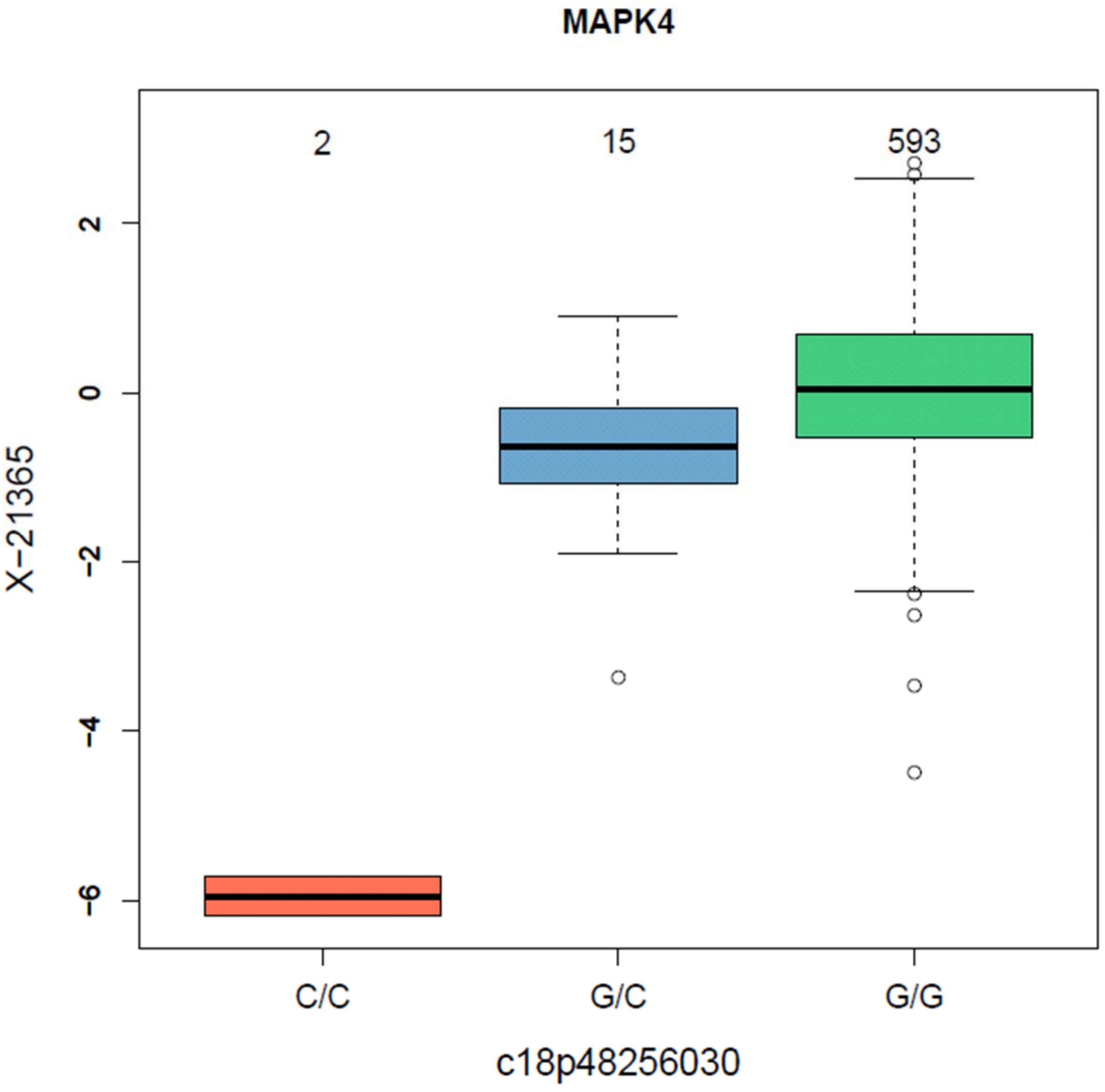
MAPK4
2
15
593
2
0
-2
-4
-6
X−21365
C/C
G/C
G/G
c18p48256030

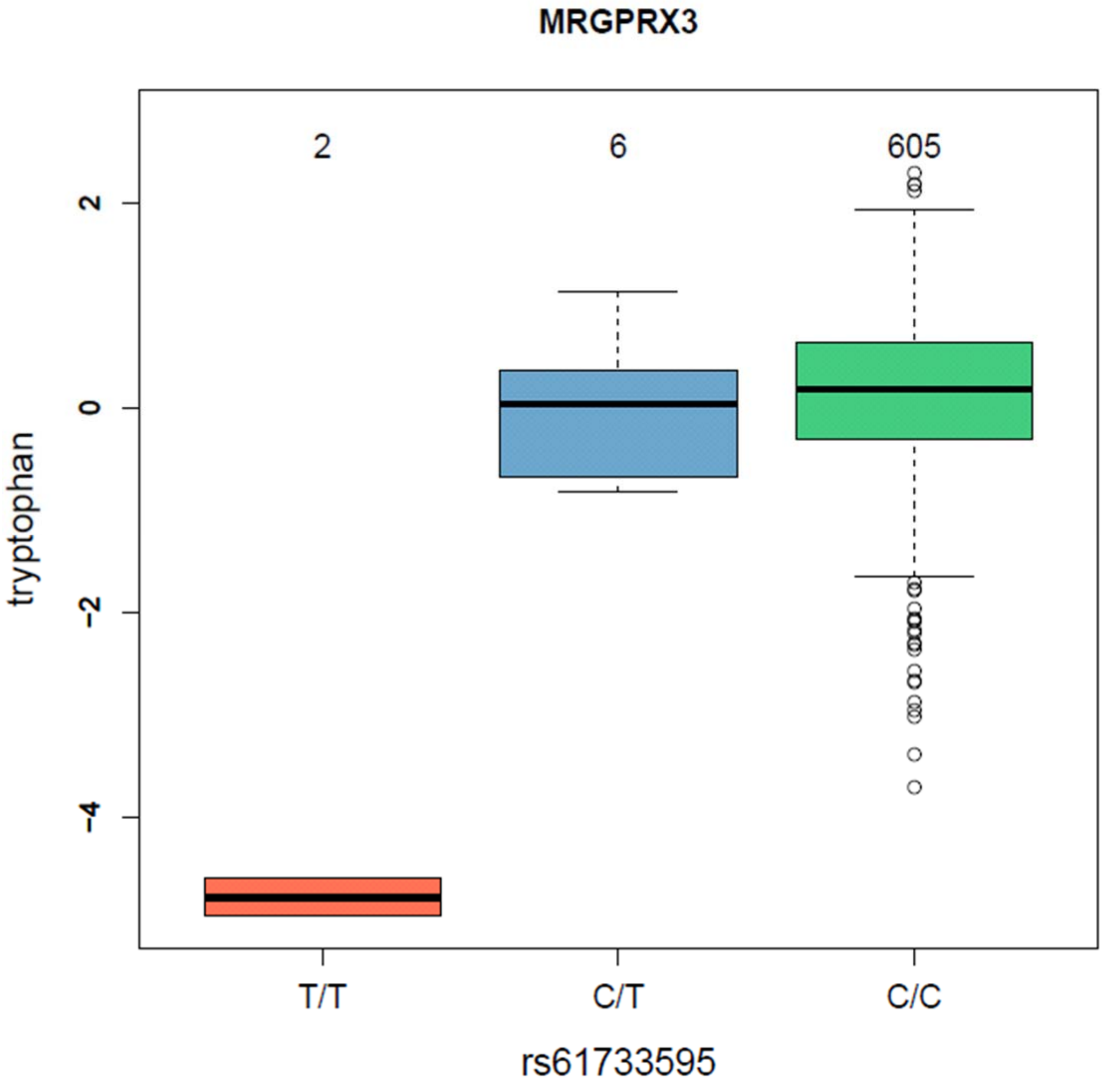
MRGPRX3
2
6
605
2
0
-2
-4
tryptophan
T/T
C/T
C/C
rs61733595

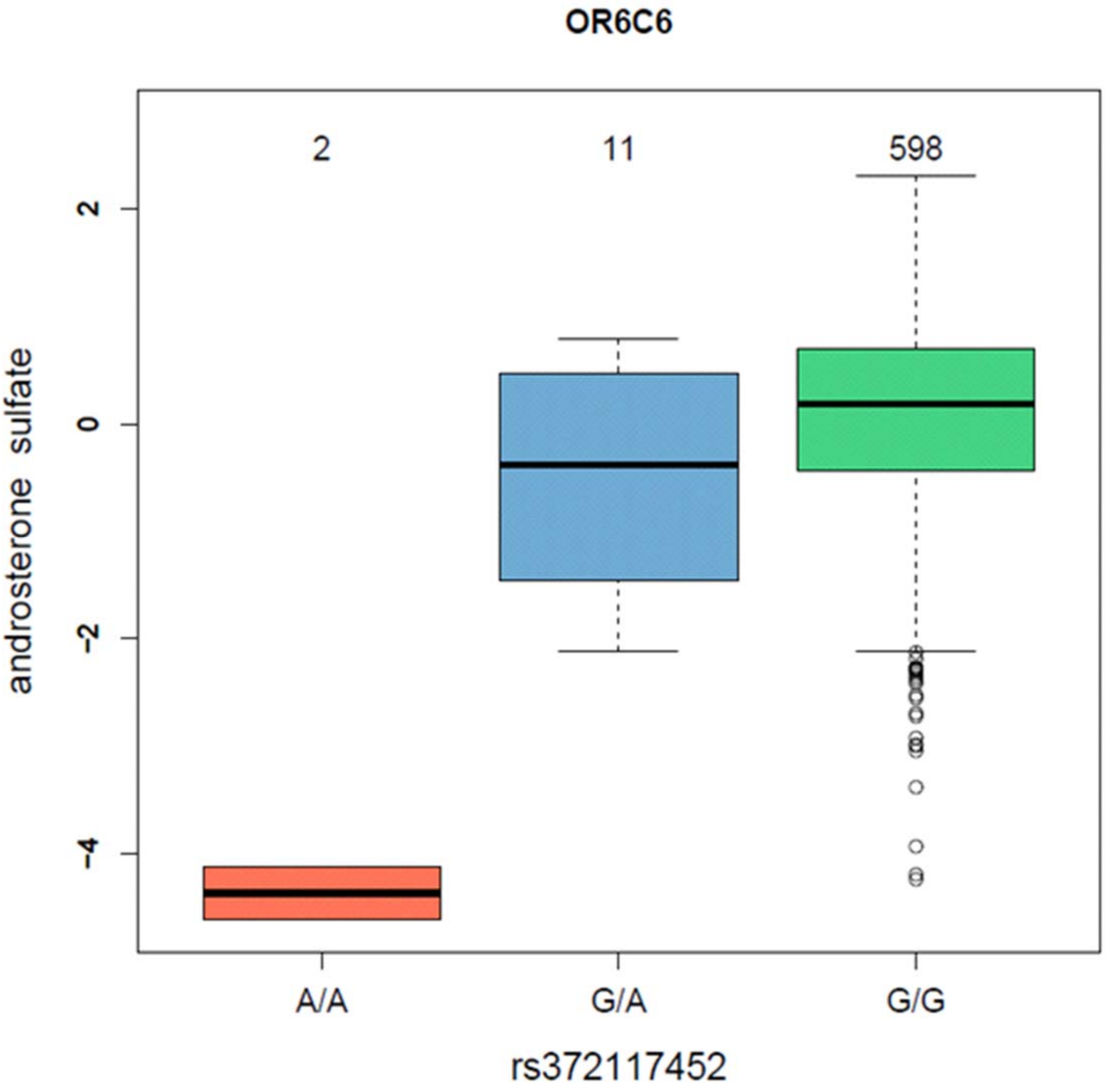
OR6C6
2
11
598
2
0
-2
-4
androsterone sulfate
A/A
G/A
G/G
rs372117452

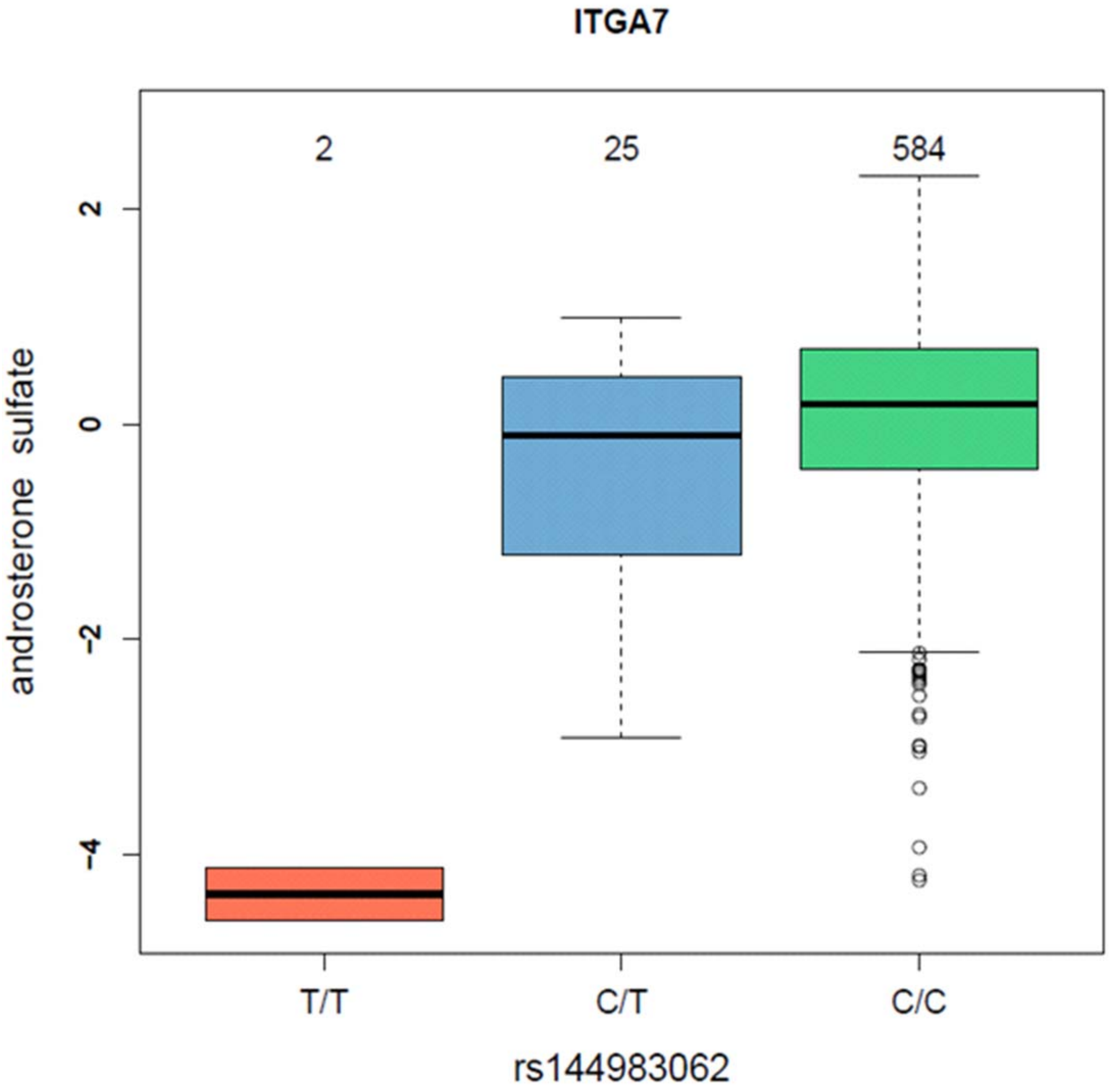

ITGA7
2
25
584
2
0
-2
-4
androsterone sulfate
T/T
C/T
C/C
rs144983062

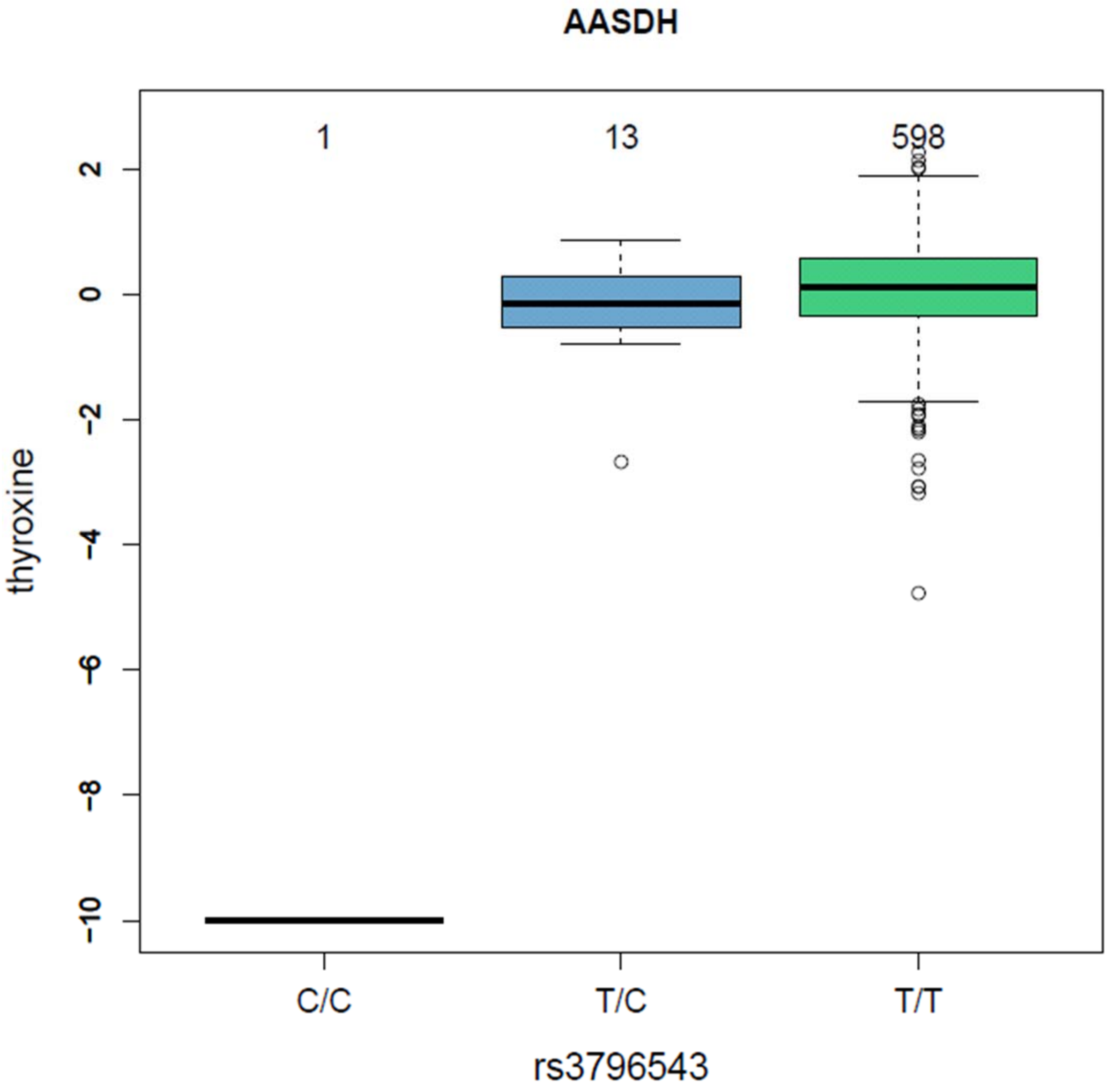
AASDH
1
13
598
2
0
-2
-4
-6
-8
-10
thyroxine
C/C
T/C
T/T
rs3796543

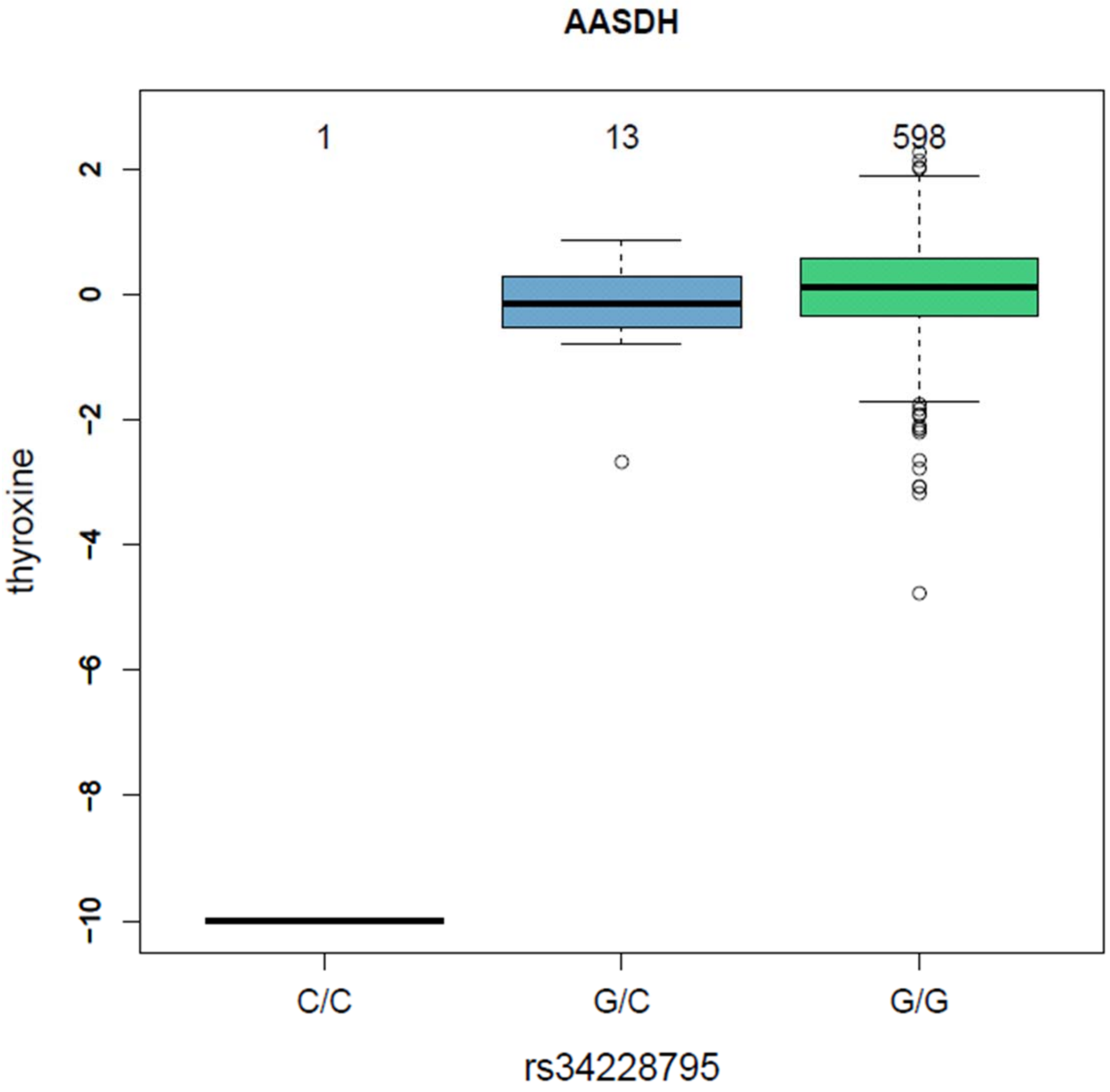
AASDH
1
13
598
2
0
-2
-4
-6
-8
-10
thyroxine
C/C
G/C
G/G
rs34228795

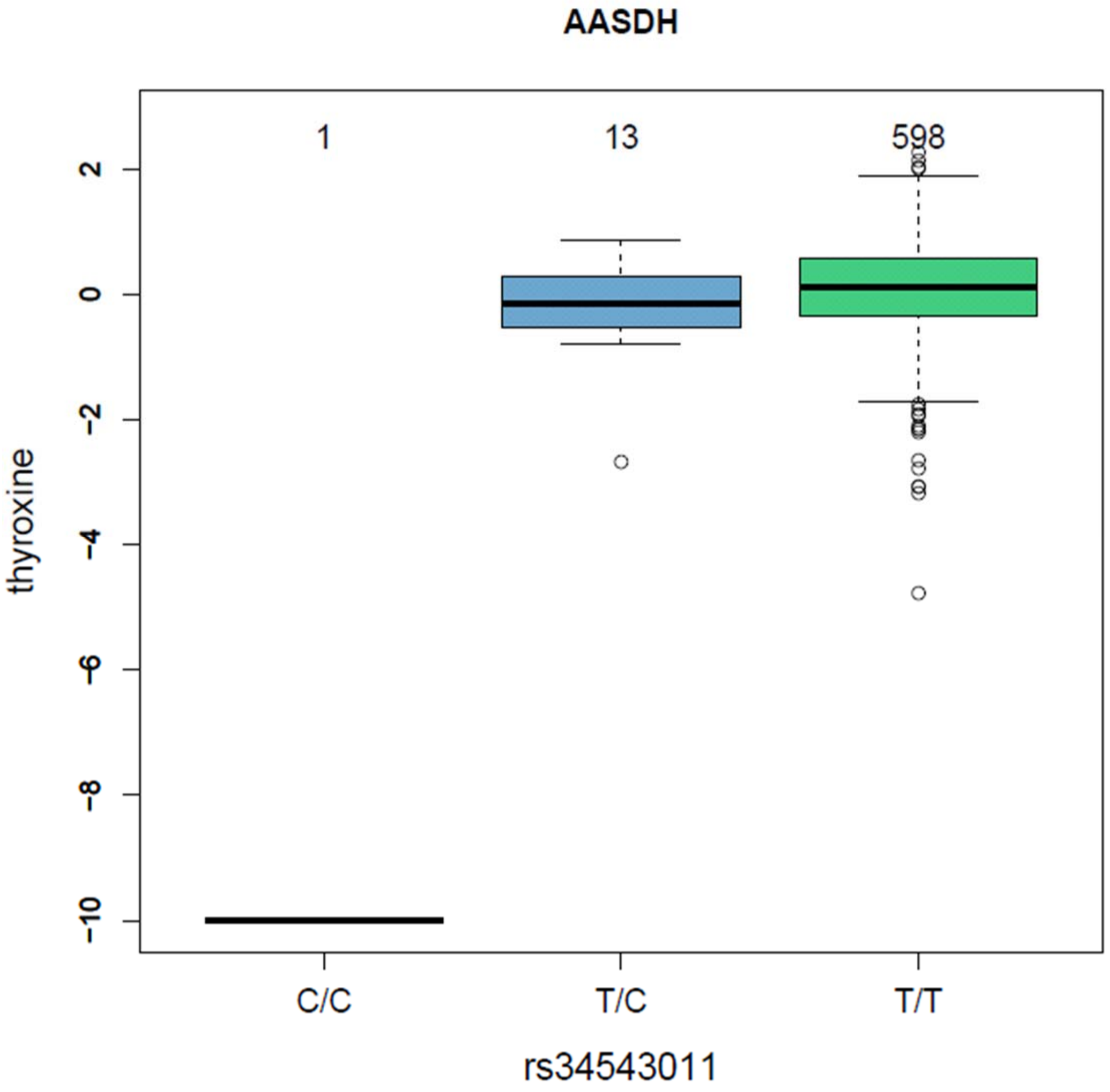

AASDH
1
13
598
2
0
-2
-4
-6
-8
-10
thyroxine
C/C
T/C
T/T
rs34543011

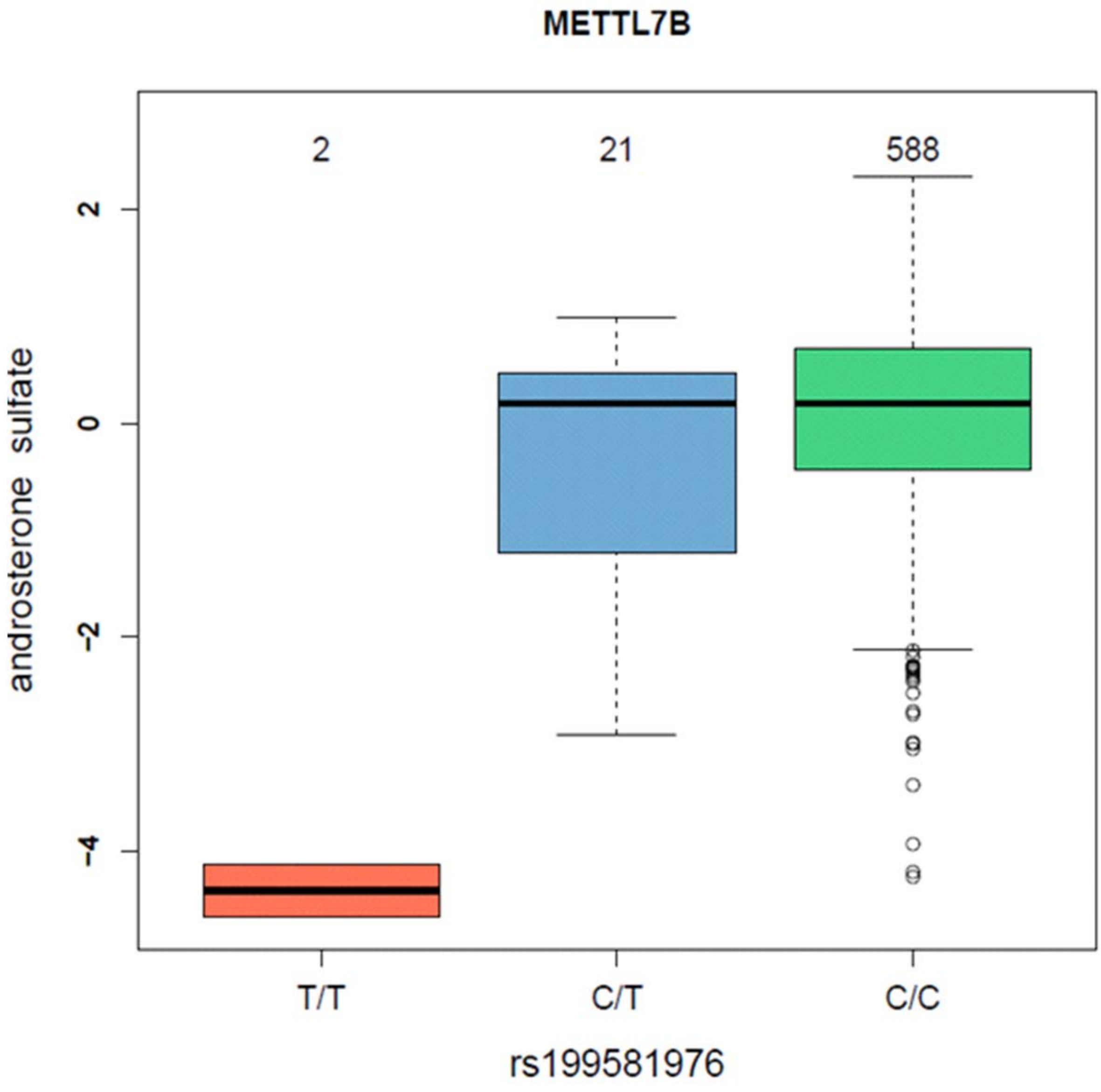
METTL7B
2
21
588
2
0
-2
-4
androsterone sulfate
T/T
C/T
C/C
rs199581976

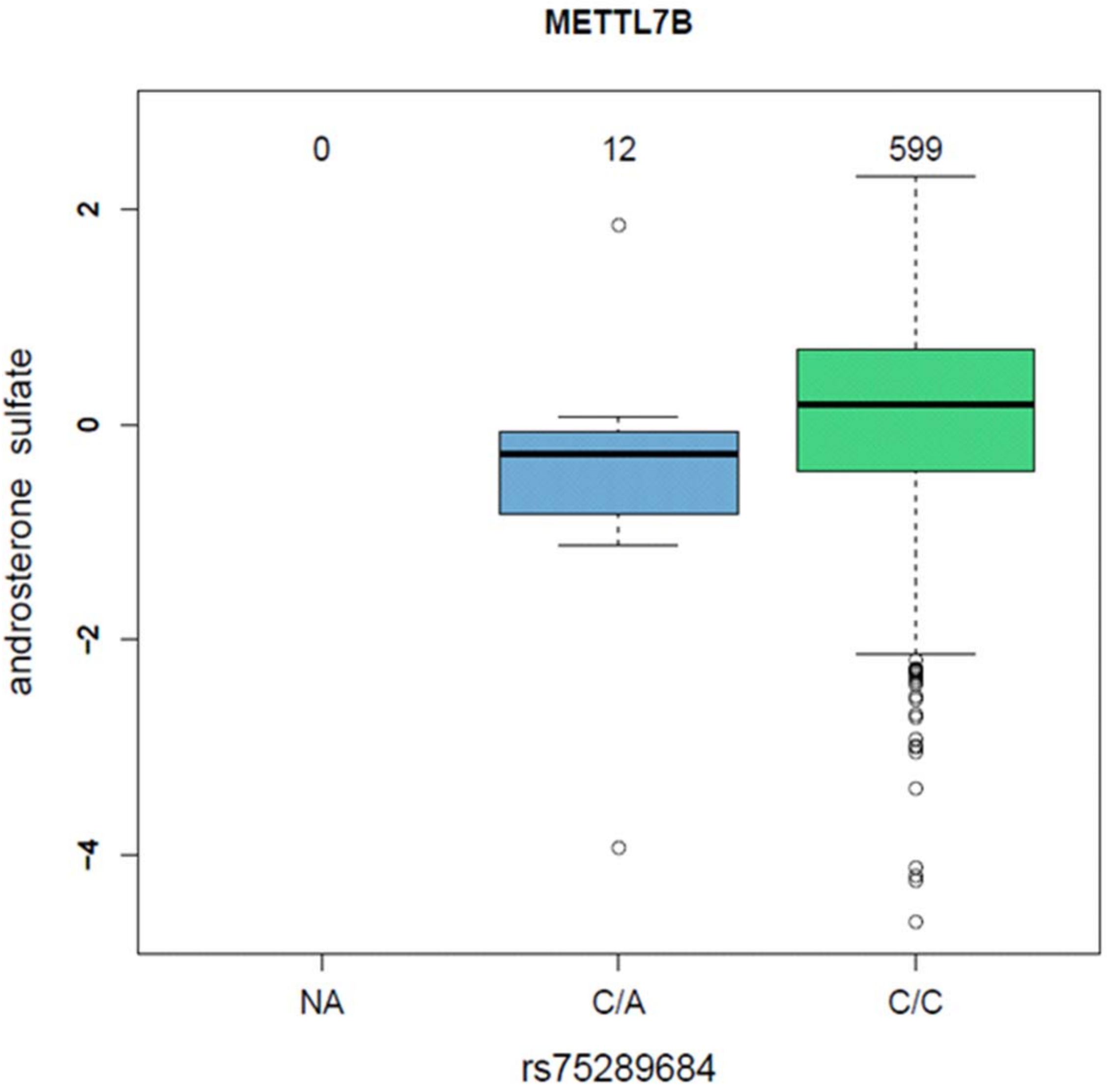
METTL7B
0
12
599
2
0
-2
-4
androsterone sulfate
NA
C/A
C/C
rs75289684

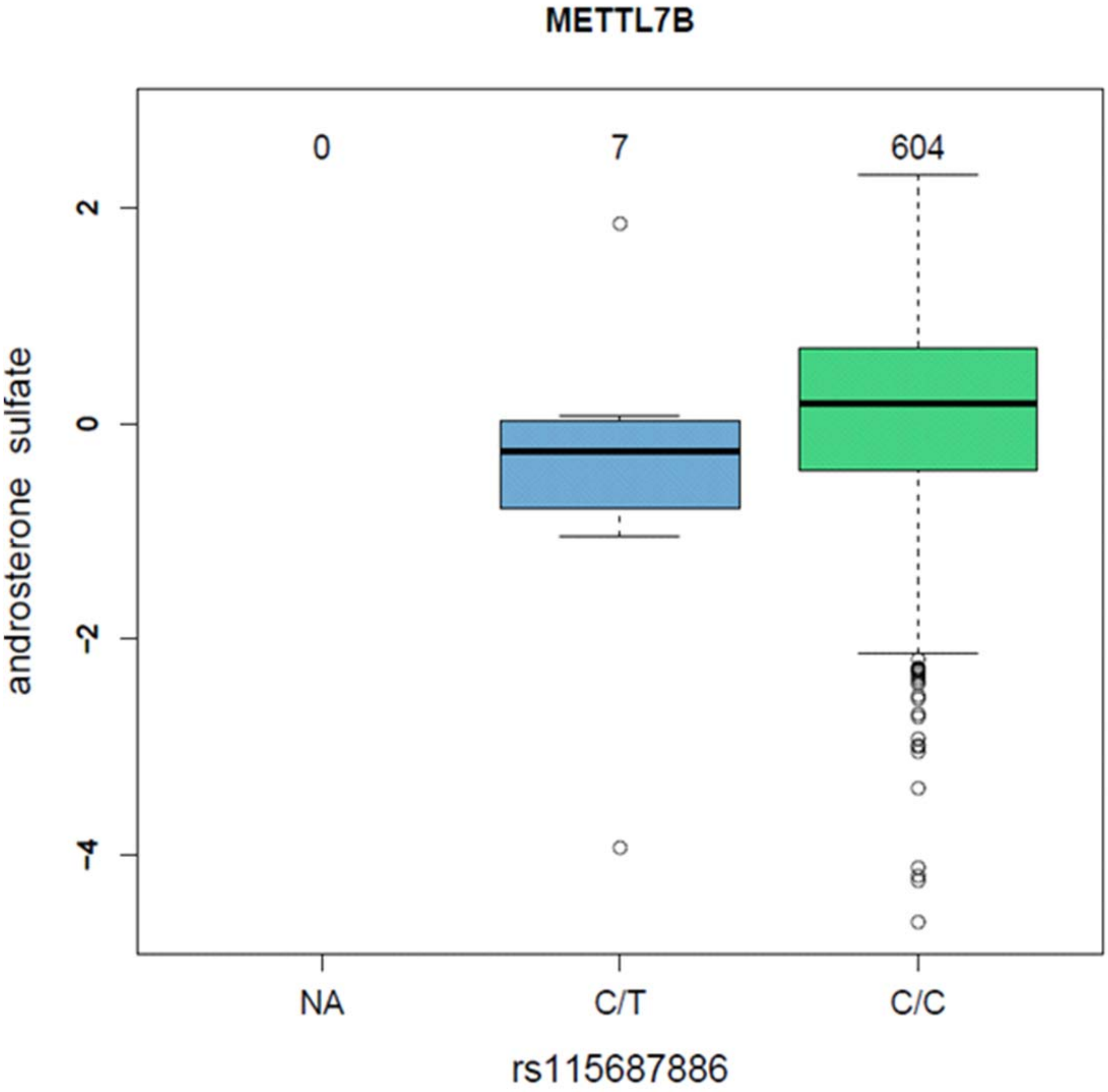
METTL7B
0
7
604
2
0
-2
-4
androsterone sulfate
NA
C/T
C/C
rs115687886

Supplementary Figure 6:

Spectra of candidate molecules and the structurally unknown metabolites. The extracted ion chromatograms show the same retention time for each measurement: the unknown metabolite in a reference plasma matrix, the candidate molecule in a neat solution, and the candidate molecule spiked into a reference plasma matrix (containing the unknown metabolite). The MS/MS fragmentation spectra of the candidate molecule and of the unknown metabolite show the same fragments with equal relative intensities; consequently, the candidate molecule is verified.

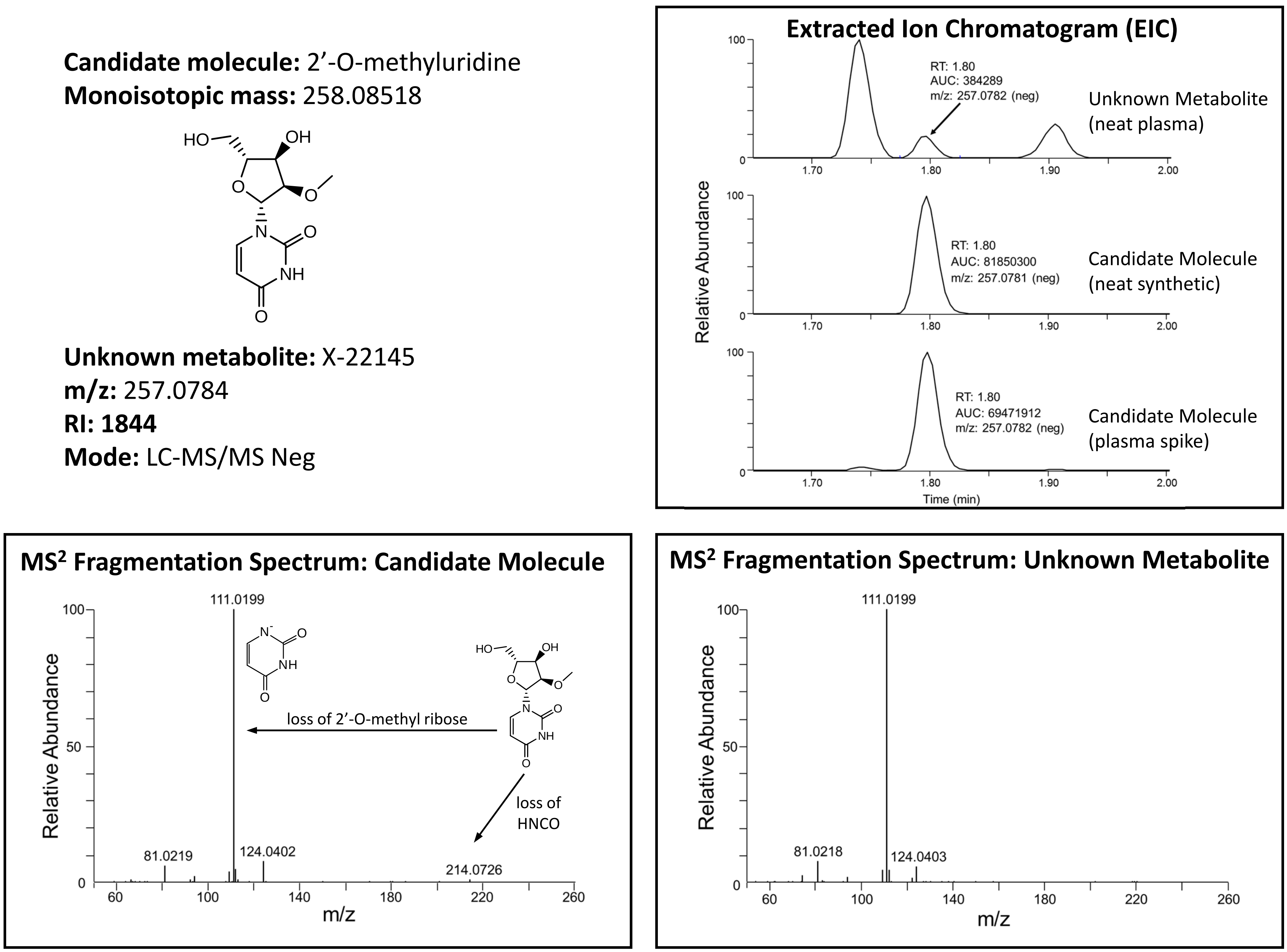
Candidate molecule: 2’-O-methyluridine
Monoisotopic mass: 258.08518
Unknown metabolite: X-22145
m/z: 257.0784
RI: 1844
Mode: LC-MS/MS Neg
Extracted Ion Chromatogram (EIC)
RT: 1.80
AUC: 384289
m/z: 257.0782 (neg)
Unknown Metabolite (neat plasma)
RT: 1.80
AUC: 81850300
m/z: 257.0781 (neg)
Candidate Molecule (neat synthetic)
RT: 1.80
AUC: 69471912
m/z: 257.0782 (neg)
Candidate Molecule (plasma spike)
Relative Abundance
Time (min)
MS² Fragmentation Spectrum: Candidate Molecule
111.0199
81.0219
124.0402
214.0726
loss of 2’-O-methyl ribose
loss of HNCO
Relative Abundance
m/z
MS² Fragmentation Spectrum: Unknown Metabolite
111.0199
81.0218
124.0403
Relative Abundance
m/z

(a)

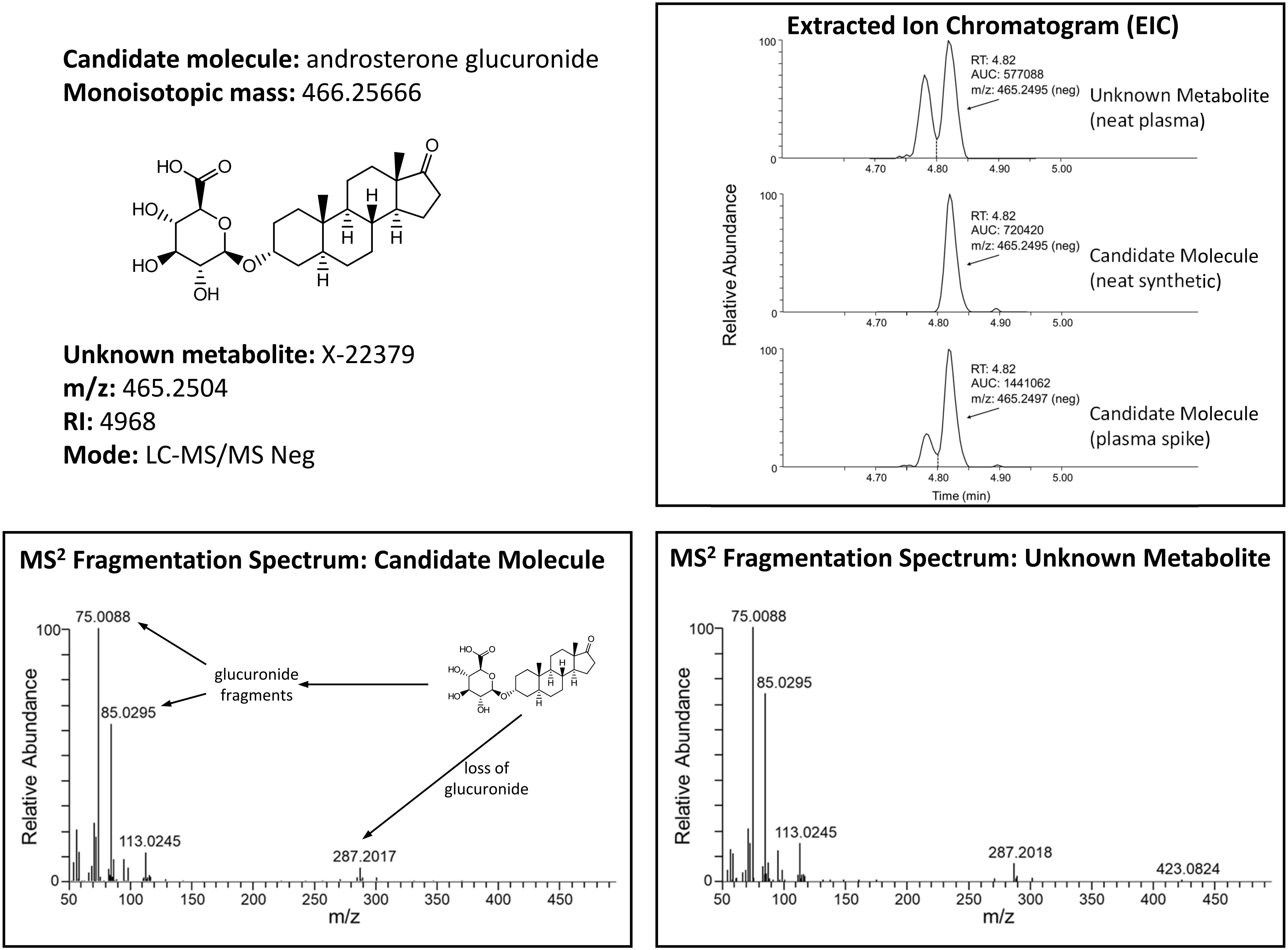

Candidate molecule: androsterone glucuronide
Monoisotopic mass: 466.25666
Unknown metabolite: X-22379
m/z: 465.2504
RI: 4968
Mode: LC-MS/MS Neg
Extracted Ion Chromatogram (EIC)
RT: 4.82
AUC: 577088
m/z: 465.2495 (neg)
Unknown Metabolite (neat plasma)
RT: 4.82
AUC: 720420
m/z: 465.2495 (neg)
Candidate Molecule (neat synthetic)
RT: 4.82
AUC: 1441062
m/z: 465.2497 (neg)
Candidate Molecule (plasma spike)
Relative Abundance
Time (min)
MS² Fragmentation Spectrum: Candidate Molecule
75.0088
85.0295
113.0245
287.2017
glucuronide fragments
loss of glucuronide
Relative Abundance
m/z
MS² Fragmentation Spectrum: Unknown Metabolite
75.0088
85.0295
113.0245
287.2018
423.0824
Relative Abundance
m/z

(b)

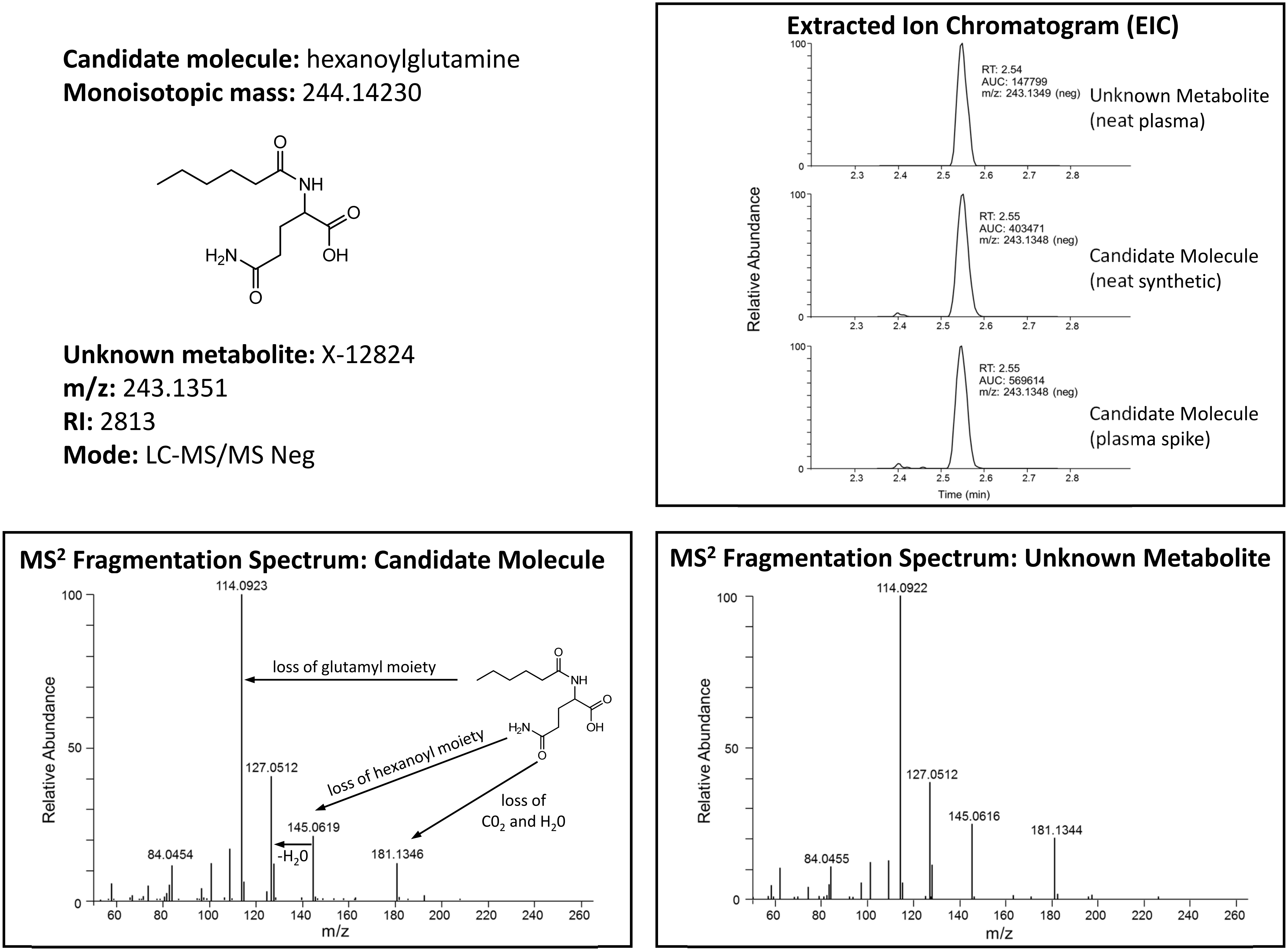

Candidate molecule: hexanoylglutamine
Monoisotopic mass: 244.14230
Unknown metabolite: X-12824
m/z: 243.1351
RI: 2813
Mode: LC-MS/MS Neg
Extracted Ion Chromatogram (EIC)
Relative Abundance
RT: 2.54
AUC: 147799
m/z: 243.1349 (neg)
Unknown Metabolite (neat plasma)
RT: 2.55
AUC: 403471
m/z: 243.1348 (neg)
Candidate Molecule (neat synthetic)
RT: 2.55
AUC: 569614
m/z: 243.1348 (neg)
Candidate Molecule (plasma spike)
Time (min)
MS² Fragmentation Spectrum: Candidate Molecule
Relative Abundance
114.0923
loss of glutamyl moiety
loss of hexanoyl moiety
loss of C0₂ and H₂0
127.0512
145.0619
-H₂0
84.0454
181.1346
m/z
MS² Fragmentation Spectrum: Unknown Metabolite
Relative Abundance
114.0922
127.0512
145.0616
181.1344
84.0455
m/z

(c)

Candidate molecule: N-trimethyl 5-aminovalerate
Monoisotopic mass: 159.12592

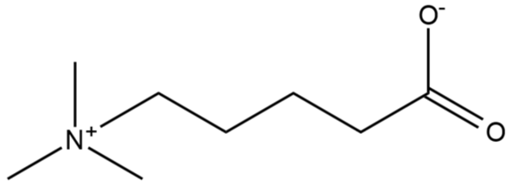

Unknown metabolite: X-21365
m/z: 160.1333
RI: 2195
Mode: LC-MS/MS Pos

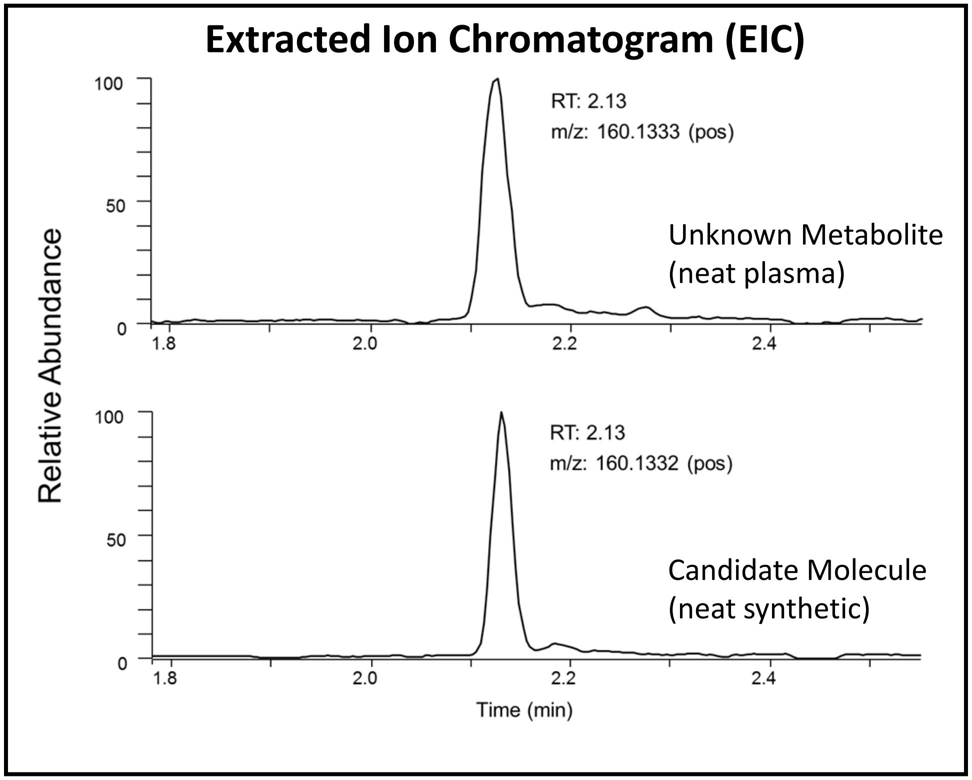

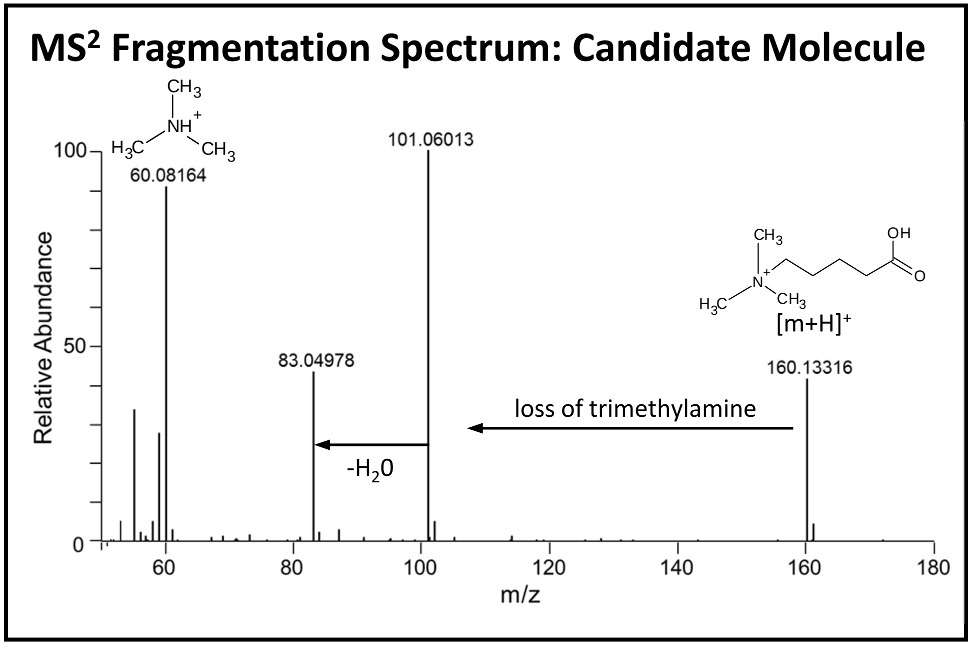

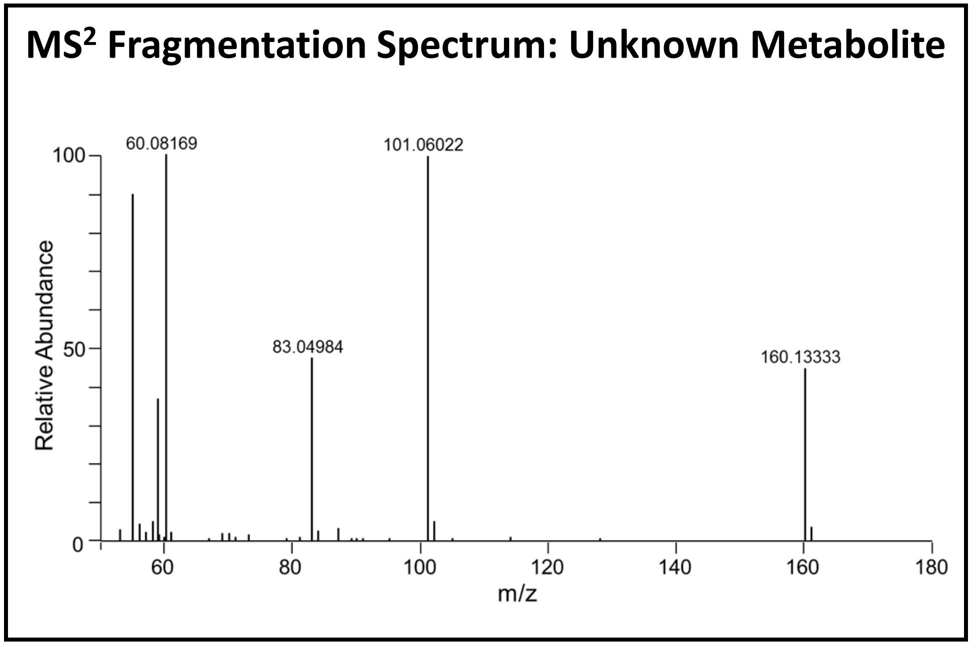

(d)

Supplementary Note 1: Materials and Methods for Global Metabolomics

Sample Preparation for Global Metabolomics

Samples were stored at –80°C until processed. Sample preparation was carried out as described previously [1] at Metabolon, Inc. Briefly, recovery standards were added prior to the first step in the extraction process for quality control purposes. To remove protein, dissociate small molecules bound to protein or trapped in the precipitated protein matrix, and to recover chemically diverse metabolites, proteins were precipitated with methanol under vigorous shaking for 2 min (Glen Mills Genogrinder 2000) followed by centrifugation. The resulting extract was divided into five fractions for analysis by ultra high performance liquid chromatography-tandem mass spectrometry (UPLC-MS/MS): 1) acidic positive ion conditions, chromatographically optimized for more hydrophilic compounds; 2) acidic positive ion conditions, chromatographically optimized for more hydrophobic compounds; 3) basic negative ion optimized conditions using a separate dedicated C18 column; 4) negative ionization following elution from a HILIC column; 5) reserved for backup.

Three types of controls were analyzed in concert with the experimental samples: samples generated from a pool of human plasma extensively characterized by Metabolon, Inc. served as technical replicate throughout the data set; extracted water samples served as process blanks; and a cocktail of standards spiked into every analyzed sample allowed instrument performance monitoring. Instrument variability was determined by calculating the median relative standard deviation (RSD) for the standards that were added to each sample prior to injection into the mass spectrometers (median RSD = 4%; n ≥ 30 standards). Overall process variability was determined by calculating the median RSD for all endogenous metabolites (i.e., non-instrument standards) present in 100% of the pooled human plasma samples (median RSD = 9%; n = several hundred metabolites). Experimental samples and controls were randomized across the platform run.

Mass Spectrometry Analysis

Non-targeted MS analysis was performed at Metabolon, Inc. Extracts were subjected to UPLC-MS/MS [1]. The chromatography was standardized and, once the method was validated no further changes were made. As part of Metabolon's general practice, all columns were purchased from a single manufacturer's lot at the outset of experiments. All solvents were similarly purchased in bulk from a single manufacturer's lot in sufficient quantity to complete all related experiments. For each sample, vacuum-dried samples were dissolved in injection solvent containing eight or more injection standards at fixed concentrations, depending on the platform. The internal standards were used both to assure injection and chromatographic consistency. Instruments were tuned and calibrated for mass resolution and mass accuracy daily.

All methods utilized a Waters ACQUITY UPLC and a Thermo Scientific Q-Exactive high resolution/accurate mass spectrometer interfaced with a heated electrospray ionization (HESI-II) source and Orbitrap mass analyzer operated at 35,000 mass resolution. The sample extract was dried then reconstituted in solvents compatible to each of the four methods. Each reconstitution solvent contained a series of standards at fixed concentrations to ensure injection and chromatographic consistency. One aliquot was analyzed using acidic positive ion conditions, chromatographically optimized for more hydrophilic compounds. In this method, the extract was gradient eluted from a C18 column (Waters UPLC BEH C18-2.1x100 mm, 1.7 µm) using water and methanol, containing 0.05% perfluoropentanoic acid (PFPA) and 0.1% formic acid (FA). Another aliquot was also analyzed using acidic positive ion conditions; however, it was chromatographically optimized for more hydrophobic compounds. In this method, the extract was gradient eluted from the same aforementioned C18

column using methanol, acetonitrile, water, 0.05% PFPA and 0.01% FA and was operated at an overall higher organic content. Another aliquot was analyzed using basic negative ion optimized conditions using a separate dedicated C18 column. The basic extracts were gradient eluted from the column using methanol and water, however with 6.5mM Ammonium Bicarbonate at pH 8. The fourth aliquot was analyzed via negative ionization following elution from a HILIC column (Waters UPLC BEH Amide 2.1x150 mm, 1.7 µm) using a gradient consisting of water and acetonitrile with 10mM Ammonium Formate, pH 10.8. The MS analysis alternated between MS and data-dependent MSn scans using dynamic exclusion. The scan range varied slighted between methods but covered 70-1000 m/z.

Compound Identification, Quantification, and Data Curation

Metabolites were identified by automated comparison of the ion features in the experimental samples to a reference library of chemical standard entries that included retention time, molecular weight (m/z), preferred adducts, and in-source fragments as well as associated MS spectra and curated by visual inspection for quality control using software developed at Metabolon [2, 3]. Identification of known chemical entities is based on comparison to metabolomic library entries of purified standards (The structurally named spectral library currently consists of >4,500 purified standards). Commercially available purified standard compounds have been acquired and registered into LIMS for distribution to the various UPLC-MS/MS platforms for determination of their detectable characteristics. Additional mass spectral entries have been created for structurally unnamed biochemicals, which have been identified by virtue of their recurrent nature (both chromatographic and mass spectral). These compounds have the potential to be identified by future acquisition of a matching purified standard or by classical structural analysis. Peaks were quantified using area-under-the-curve. Raw area counts for each metabolite in each sample were normalized to correct for variation resulting from instrument inter-day tuning differences by the median value for each run-day, therefore, setting the medians to 1.0 for each run. This preserved variation between samples but allowed metabolites of widely different raw peak areas to be compared on a similar graphical scale. Missing values were imputed with the observed minimum after normalization.

Supplementary Note 2: Materials and methods for the identification of unknown metabolites

A number of structurally unknown molecules have been identified based on MS/MS matches to standards. Molecules with an asterisk next to the biochemical name (*) do not have a commercial standard available but have been identified based on MS/MS fragmentation patterns and elution characteristics. The identification is followed by classification of the molecule into metabolite Super Pathway/Sub Pathway. **Supplementary Figure 6** shows the details of comparison of MS/MS fragmentation and Extracted Ion Chromatogram for the four identified unknowns X-22145 (Supplementary Figure 6.a), X-22379 (Supplementary Figure 6.b), X-12824 (Supplementary Figure 6.c) and X-21365 (Supplementary Figure 6.d).

X-22145: Identified as 2'-O-methyluridine based on a standard from AK Scientific; Nucleotide / Pyrimidine Metabolism; Uracil containing.
X-22379: Identified as androsterone glucuronide based on chemical synthesis; Lipid / Androgenic Steroids.
X-12824: Identified as hexanoylglutamine based on chemical synthesis; Lipid / Fatty Acid Metabolism (Acyl Glutamine).
X-21365: Identified as N-trimethyl 5-aminovalerate based on chemical synthesis; Amino Acid / Lysine Metabolism.

X-24438: Identified as PC(P-16:0/20:3)* - aka, 1-(1-enyl-palmitoyl)-2-dihomo-linolenoyl-sn-glycero-3-phosphocholine; Phosphatidylcholine / PC Plasmalogen
X-24439: Identified as PE(P-16:0/20:3)* - aka, 1-(1-enyl-palmitoyl)-2-dihomo-linolenoyl-sn-glycero-3-phosphoethanolamine; Phosphatidylethanolamine / PE Plasmalogen
X-12511: Identified as N-acetyl-2-aminooctanoate* - chemical standard is not available commercially; Lipid / Fatty Acid, Amino

For the following molecules, tentative details have been assigned primarily based on high-resolution accurate mass MS/MS fragmentation:
X-12844: tentatively identified as glucuronidated steroid.
X-11491: tentatively identified as deoxycholic acid glucuronide or isomer.

Synthesis of standards for confirmation of structurally unnamed molecules

For the structurally unnamed molecules X-22379, X-12824 and X-21365, the following methods outline the routes of synthesis of standards used by Metabolon for confirmation of these unknowns. Identification of X-22145 is based on a comparison to a standard from AK Scientific.

androsterone glucuronide (for confirming the identity of X-22379)
This compound was prepared according to the Koenigs-Knorr procedure, a commonly used method to synthesize glucuronides [4]. A suspension of androsterone (1.0 mg, 0.0034 mmol, 1.0 eq), acetobromo-α-D-glucuronic acid methyl ester (1.2 mg, 0.0030. 0.9 eq), and Ag_2CO_3 (1.2 mg, 0.0044, 1.3 eq) was stirred overnight at 65 °C. The reaction mixture was judged for completeness (i.e. conversion to the title compound) by LC/MS by diluting a small sample of the reaction mixture (~5 µL) into a 2 mL LC vial with 7:1 (MeOH:1N NaOH). When complete, the reaction mixture was filtered, diluted with about 2 mL of 7:1 (MeOH:1N NaOH), and concentrated to dryness. This material was further analyzed without any additional purification.

hexanoylglutamine (for confirming the identity of X-12824)
This compound was prepared according to a procedure used to form glutamine conjugates of long-chain fatty acids [5]. Pivaloyl chloride (46 µL, 0.374, 1.1 eq) was added to a stirring solution of hexanoic acid (40.0 mg, 0.341 mmol, 1.0 eq) and triethylamine (57 µL, 0.409 mmol, 1.2 eq) in tetrahydrofuran (THF; 2.4 mL) at 0 °C. Separately, a solution of glutamine (99.0 mg, 0.680 mmol, 2.0 eq) in water (0.80 mL) was prepared. After stirring for 2 hr, the mixed ester solution was filtered, washed with THF (2.5 mL), and diluted with 2.2 mL of 1,4-dioxane. This solution was added to the aqueous glutamine solution. After stirring for 20 min, triethylamine (48 µL, 0.344 mmol, 1.0 eq) was added, and the reaction mixture was stirred for several hours. When the reaction was judged complete by LC/MS, the pH was adjusted to about 3 with 10% HCl. The mixture was diluted with water and extracted twice with dichloromethane. The combined organic extracts were dried over Na_2SO_4, filtered, and concentrated to provide the crude product (28 mg) as an oil. This material was further analyzed without any additional purification.

N-trimethyl 5-aminovalerate (for confirming the identity of X-21365)
This compound was prepared using a standard amine alkylation procedure [6]. Iodomethane (20 µL, 0.322 mmol, 38 eq) was added to a stirring solution of 5-aminovaleric acid (1.0 mg, 0.0085 mmol, 1.0 eq) in 1:1 water/MeOH (~ 0.4 mL). After stirring for several hours, the reaction was judged complete by LC/MS and concentrated to dryness. This material was further analyzed without any additional purification.

While NMR is an important step for confirming the structure of the synthesized standards, NMR data was not obtained for the synthesized standards of androsterone glucuronide, hexanoylglutamine, and N-trimethyl 5-aminovalerate. As outlined, their syntheses were performed using a single-step method in which only a single reaction product was expected. Indeed, when the product of each synthesis was analyzed by high-resolution accurate mass spectrometry, a single peak of the correct mass was detected. Moreover, the expected accurate mass fragmentations for the molecular ions were observed, which further supported the formation of the expected products. Thus, for these simple chemical syntheses, structural confirmation by NMR was unlikely to yield additional information beyond what high-resolution accurate mass spectrometry provided, and NMR was not performed.

Supplementary References:

1. Evans AM, Bridgewater BR, Liu Q, Mitchell MW, Robinson RJ, Dai H, Stewart SJ, DeHaven CD, and Miller LAD (2014) High Resolution Mass Spectrometry Improves Data Quantity and Quality as Compared to Unit Mass Resolution Mass Spectrometry in High-Throughput Profiling Metabolomics. Metabolomics 4:132. doi: 10.4172/2153-0769.1000132
2. DeHaven CD, Barrett T, Mitchell M, Milgram E (2009) Integrated, nontargeted ultrahigh performance liquid chromatography/electrospray ionization tandem mass spectrometry platform for the identification and relative quantification of the small-molecule complement of biological systems. Anal Chem 81: 6656–6667.
3. DeHaven C, Evans A, Dai H, Lawton K (2010) Organization of GC/MS and LC/MS metabolomics data into chemical libraries. Journal of Cheminformatics 2: 9.
4. Stachulski, A.; Jenkins, G. The synthesis of O-glucuronides. Natural Product Reports. 1998, 173-186.
5. Itoh, S.; Kuwahara, S.; Hasegawa, M.; Kodama, O. Synthesis of the (17R)- and (17S)-Isomers of Volicitin, an Elicitor of Plant Volatiles Contained in the Oral Secretion of the Beet Armyworm. Biosci. Biotechnol. Biochem. 2002, 66 (7), 1591–1596.
6. Iyengar, R. R. and Bhattacharya, P. K. Thermodynamic view of hydrophobic association of side chains of aromatic amino acids. Indian Journal of Chemistry, Section A: Inorganic, Physical, Theoretical & Analytical. 1989, 28A(6), 445-51.
