# Supplementary material for: Whole-exome sequencing identifies common and rare variant metabolic QTLs in a Middle Eastern population
Source: Nat Commun. 2018 Jan 23;9:333. doi: 10.1038/s41467-017-01972-9 (PMC5780481; doi:10.1038/s41467-017-01972-9)
Supplement: Supplementary file 2 — Description of Additional Supplementary Files [file 41467_2017_1972_MOESM2_ESM.pdf]

Description of Additional Supplementary Files

File Name: Supplementary Dataset 1
Description: Metabolite information

File Name: Supplementary Dataset 2
Description: 145 loci replication results.

File Name: Supplementary Dataset 3
Description: Details of 21 discovered loci with sentinel metabolite/ratio and non-replicated loci.

File Name: Supplementary Dataset 4
Description: Array replication p-values for the discovered loci.

File Name: Supplementary Dataset 5
Description: All significant associations found in the discovered loci.

File Name: Supplementary Dataset 6
Description: Information on Metabolites, Metabolite-loci relation, relation to diseases, drugs, pharmacology, eQTL information, etc.

File Name: Supplementary Dataset 7
Description: All single metabolite associations identified at $p<=1.4 \times 10^{-7}$.
